# B7-1 mediates podocyte injury and glomerulosclerosis through communication with Hsp90ab1-LRP5-β-catenin pathway

**DOI:** 10.1038/s41418-022-01026-8

**Published:** 2022-06-16

**Authors:** Jiemei Li, Jing Niu, Wenjian Min, Jun Ai, Xu Lin, Jinhua Miao, Shan Zhou, Ye Liang, Shuangqin Chen, Qian Ren, Kunyu Shen, Qinyu Wu, Xiaolong Li, Weiwei Shen, Fan Fan Hou, Youhua Liu, Peng Yang, Lili Zhou

**Affiliations:** 1 Division of Nephrology, Nanfang Hospital, Southern Medical University; National Clinical Research Center for Kidney Disease; State Key Laboratory of Organ Failure Research; Guangdong Provincial Institute of Nephrology; Guangdong Provincial Key Laboratory of Renal Failure Research, Guangzhou, 510515 China; 2 Division of Nephrology, Jiangsu Province Hospital of Chinese Medicine, Affiliated Hospital of Nanjing University of Chinese Medicine, Nanjing, China; 3 State Key Laboratory of Natural Medicines and Jiang Su Key Laboratory of Drug Design and Optimization, China Pharmaceutical University, Nanjing, China; 4 Department of Nephrology, The Affiliated Hospital of Youjiang Medical University for Nationalities, Baise, Guangxi China; 5 Department of Pathology, University of Pittsburgh School of Medicine, Pittsburgh, PA USA

**Keywords:** Kidney diseases, Cell biology

## Abstract

Podocyte injury is a hallmark of glomerular diseases; however, the underlying mechanisms remain unclear. B7-1 is increased in injured podocytes, but its intrinsic role is controversial. The clinical data here revealed the intimate correlation of urinary B7-1 with severity of glomerular injury. Through transcriptomic and biological assays in B7-1 transgenic and adriamycin nephropathy models, we identified B7-1 is a key mediator in podocyte injury and glomerulosclerosis through a series of signal transmission to β-catenin. Using LC-MS/MS, Hsp90ab1, a conserved molecular chaperone, was distinguished to be an anchor for transmitting signals from B7-1 to β-catenin. Molecular docking and subsequent mutant analysis further identified the residue K69 in the N terminal domain of Hsp90ab1 was the key binding site for B7-1 to activate LRP5/β-catenin pathway. The interaction and biological functions of B7-1-Hsp90ab1-LRP5 complex were further demonstrated in vitro and in vivo. We also found B7-1 is a novel downstream target of β-catenin. Our results indicate an intercrossed network of B7-1, which collectively induces podocyte injury and glomerulosclerosis. Our study provides an important clue to improve the therapeutic strategies to target B7-1.

## Introduction

Glomerular disease is the major cause of chronic kidney disease (CKD) and end-stage renal disease (ESRD) [1]. Podocyte is the main component of the glomerular filter barrier. With high energy consumption and the inability to proliferate, podocytes are vulnerable to stimuli such as oxidative stress and immune attack [2–5]. Injured podocyte can go through dedifferentiation, apoptosis, and also senescence [4, 6, 7], which play key roles in the pathogenesis of diabetic nephropathy (DN), lupus nephritis (LN), IgA nephropathy (IgAN), membranous nephropathy (MN), minimal change disease (MCD), and other glomerular diseases [8–12]. However, the underlying mechanisms are not elucidated.

Recent reports found podocyte may also function as a non-hematopoietic antigen-presenting cell (APC) to be involved in glomerular nephritis [5, 13]. Like other APC cells such as dendritic cells or macrophages, podocytes express MHC class II molecules [13, 14] and importantly, the co-stimulatory molecule B7-1 [15, 16], also known as CD80, to initiate T cell activation and inflammation [17]. Commonly, APC cells present antigens to T lymphocytes to trigger inflammation [18–20]. B7-1 plays an important role in this process [21]. Whereas, as non-hematopoietic APC, podocyte expresses B7-1 to possibly result into self-injury [15]. However, the intrinsic role of B7-1 in podocyte injury and glomerular diseases remains largely unknown.

Podocyte B7-1 was firstly identified in 2004 [15], after that it was inconsistently detected in the studies of clinical nephropathy [22–24]. In addition, the therapeutic effects of abatacept, a CTLA4-immunoglobulin fusion protein (CTLA4-Ig) which blocks the interaction between B7-1 (APC) and CD28 (T cell), are still in uncertainty in glomerular diseases [25]. CTLA4-Ig has already been investigated in primary and recurrent focal segmental glomerulosclerosis (FSGS), DN, and MCD [26–29]. Although there is a remarkable remission of proteinuria among some patients during medication [29]; however, relapse occurs after withdrawal of treatment [30]. It suggests the immune response is not the only reason for B7-1-mediated glomerular diseases. Indeed, B7-1 could inactivate integrin α3β1 [31] and disrupts NEPH1 signaling [32] to induce podocyte injury and promote albuminuria. These results suggest that B7-1 could solely mediate podocyte injury independent of immune involvement. However, the underlying mechanisms have not been completely clarified.

Wnt/β-catenin is a developmental signaling, but highly reactivated in CKD [33]. The low-density lipid receptor family members LRP5/6 are the key components of Wnt receptor complexes [34]. The phosphorylation of LRP5/6 leads to Wnt signal transmission to β-catenin, resulting into β-catenin’s translocation into nuclei to activate targeted genes such as RAS systems [35]. Our previous report found β-catenin crucially contributes to podocyte injury through ubiquitinated degradation of WT1 [36], a key transcription factor for podocyte differentiation [33, 36]. Hence, B7-1 and β-catenin, could possibly have an intimate correlation in podocyte injury. But this should be determined in detail.

In this study, we identified β-catenin is an important mediator in B7-1 signaling. Furthermore, Hsp90ab1 mediates the communication between B7-1 and LRP5/β-catenin signaling in podocyte injury. Our study provides the important mechanisms of podocyte injury, and improves the understanding of strategies targeting B7-1.

## Results

### Podocyte B7-1 is upregulated in a variety of glomerular diseases and accompanied by β-catenin activation

**Fig. 1:**
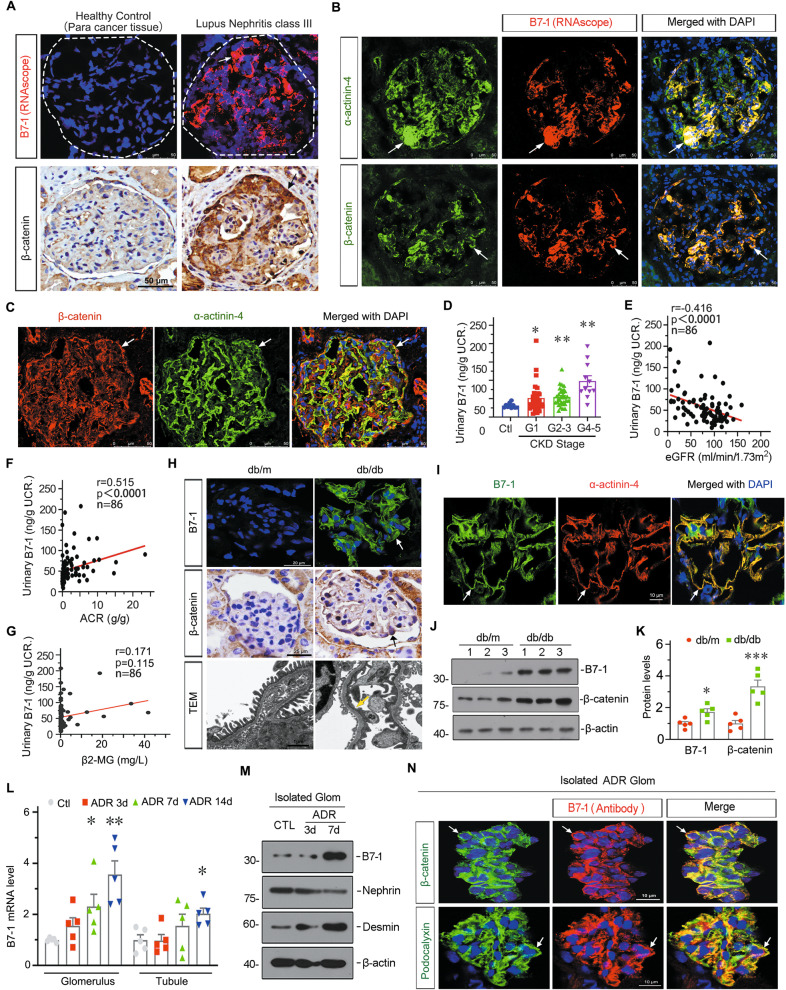
Podocyte B7-1 is upregulated in a variety of glomerular diseases and associates with β-catenin. **A** Representative micrographs of B7-1 RNAscope staining and β-catenin immunochemistry staining in human kidney cortical tissue from CKD patients with lupus nephritis class III (LN), and the healthy control was from paracancerous tissue. Arrows indicate positive staining. Bar = 50 μm. **B**, **C** Representative micrographs showing the colocalization of B7-1 and α-actinin-4, B7-1 and β-catenin, β-catenin and α-actinin-4 in human kidney biopsy from a patient with LN. Arrows indicate positive staining. Bar = 50 μm. **D** Urinary B7-1 levels were increased following the disease progression in CKD patients. Patients were grouped according to their estimated glomerular filtration rate (eGFR) (*ml/min* per 1.73 m^2^) as indicated. *P < 0.05, **P < 0.01 versus healthy control group. Healthy control (Ctl): *n* = 10; CKD1: *n* = 33; CKD2-3: *n* = 32; CKD4-5: *n* = 11. **E**–**G** Linear regressions showing the correlation between urinary B7-1 and eGFR, urinary albumin-to-creatinine ratio (ACR) or urinary β-2 microglobulin (β2-MG) content in all subjects (*n* = 86). The spearman correlation coefficient (r) and *P* value are shown. **H** Representative images showing B7-1 IF staining (Bar = 20 μm), β-catenin IHC staining (Bar = 25 μm), and TEM micrographs (Bar = 1 μm) in diabetic db/db *m*ice and their genetic control (db/m) mice. White and black Arrows indicate positive staining. Yellow arrow indicates foot process effacement. **I** Representative micrographs showing the colocalization of B7-1 and α-actinin-4 in db/db mice, Bar = 10 μm. **J**, **K** Western blot and quantitative data showing the relative protein levels of B7-1 and β-catenin in kidneys from db/db mice and db/m mice. *P < 0.05, ***P < 0.001 versus db/m mice. **L** The relative mRNA level of B7-1 in isolated glomerulus and tubule from mice after treatment with adriamycin (ADR) at indicated time points (3, 7 or 14 days). *P < 0.05, **P < 0.01 versus control group. *n* = 5. **M** Representative Western blot showing the protein levels of B7-1, Nephrin and Desmin in isolated glomeruli at different time points after ADR injection. Each group contains five biological independent samples from mice. **N** Representative micrographs showing the co-expression of B7-1 and β-catenin, or B7-1 and Podocalyxin in isolated glomeruli from ADR mice. Arrows indicate the colocalization of B7-1 and β-catenin or podocalyxin. Bar = 10 μm. The full length original western blots for all results are provided in Supplementary File 1.

We first performed in situ RNA hybridization for B7-1 using RNAscope technology, a more specific and sensitive method compared with immunological assessment [37–39]. Compared to the negative signals in healthy control, B7-1 RNA was increased in patients with class III active LN, accompanied by β-catenin increasing (Fig. 1A). B7-1 was also upregulated in MCD, IgAN, FSGS, DN, and MN (Supplementary Fig. S1). B7-1 was highly colocalized with α-actinin-4, a podocyte marker, and β-catenin (Fig. 1B, Supplementary Fig. S1). We also observed that β-catenin was largely colocalized with α-actinin-4 (Fig. 1C, Supplementary Fig. S1). These suggest the intimate correlation between B7-1 and β-catenin in podocyte injury. The specificity of B7-1 RNAscope was testified by a negative probe control (Supplementary Fig. S1).

We next analyzed the correlation of urinary B7-1 with estimated glomerular filtration rate (eGFR), albumin/urine creatinine ratio (ACR), a marker of glomerular proteinuria, and β-2-microglobulin (β2-MG), a marker of tubular proteinuria, in clinical cohort of patients with glomerular diseases (Supplementary Table S1). As shown in Fig. 1D, urinary B7-1 levels gradually increased following the progression of CKD. Urinary B7-1 was negatively associated with eGFR, and positively associated with ACR (Fig. 1E, F), but showed no correlation with urinary β2-MG (Fig. 1G).

We further assessed B7-1 and β-catenin in db/db mice, a model of type 2 diabetes mellitus with hyperactive expression of β-catenin in podocytes [40]. As shown, B7-1 was strongly upregulated in glomerulus in db/db mice, along with β-catenin increasing in podocytes and podocyte foot process fusion (Fig. 1H). The costaining showed B7-1 was primarily upregulated in podocytes (Fig. 1I). Western blotting analysis also revealed that B7-1 upregulation was concomitant with β-catenin activation (Fig. 1J, K). The specificity of antibodies was testified by isotype IgG antibody (Supplementary Fig. S1).

To further identify the important role of B7-1 in podocyte injury, we then isolated glomeruli and tubules from mice with adriamycin (ADR) nephropathy, a FSGS model. Interestingly, we found B7-1 mRNA levels were upregulated in glomeruli in a time-dependent manner (Fig. 1L). Notably, B7-1 was significantly increased in glomeruli at 7 days after ADR treatment, a critical time point for podocyte injury [40, 41]. Whereas, B7-1 was only slightly increased in tubules at the late stage (Fig. 1L). B7-1 upregulation was concomitant with the decrease in Nephrin, a podocyte slit diagram marker, and increase in Desmin, a podocyte injury marker (Fig. 1M). Furthermore, compared to extremely weak expression in isolated control glomeruli (Supplementary Fig. S1), B7-1 was upregulated in ADR-treated glomeruli, and perfectly colocalized with β-catenin and Podocalyxin, another podocyte marker (Fig. 1N). We also performed costaining of B7-1 and Endomucin (EMCN), an endothelial cell marker, in the kidney section from ADR mice. We found that there was a very small part of colocalizaton of B7-1 with EMCN (Supplementary Fig. S1). All these results suggest B7-1 is highly involved in podocyte injury, and this possibly was mediated by β-catenin signaling.

### Podocyte-specific transgene of B7-1 in mice sufficiently induces podocyte injury and is associated with β-catenin

**Fig. 2:**
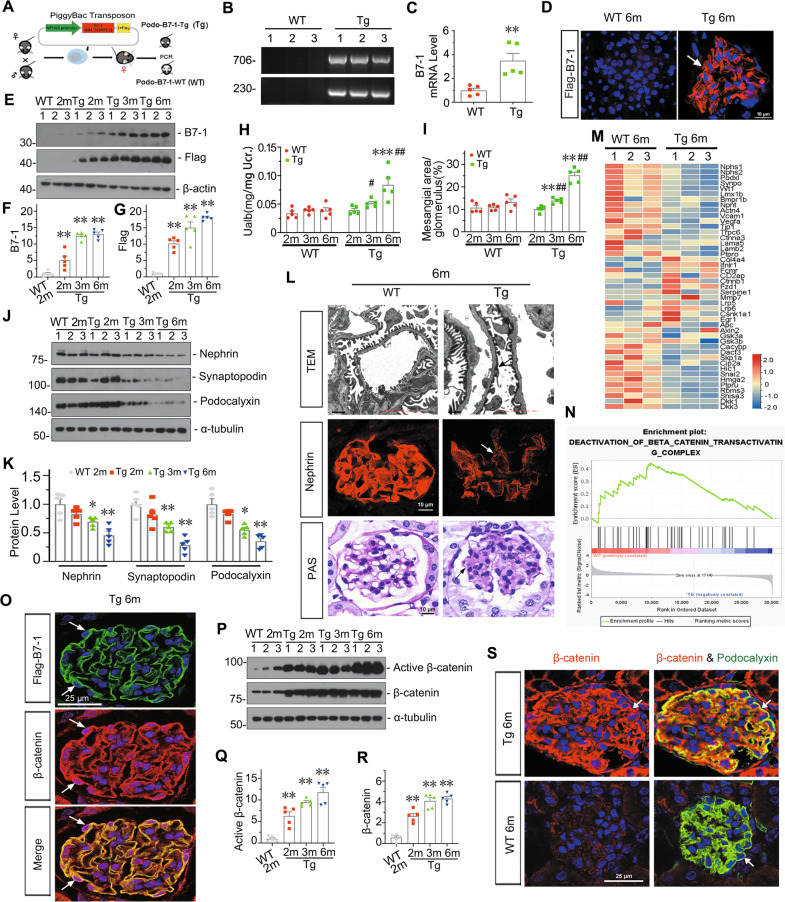
Podocyte-specific transgene of B7-1 in mice sufficiently induces podocyte injury and is associated with β-catenin. **A**, **B** Establishment of podocyte-specific B7-1 transgenic (Tg) mice. Genotyping was confirmed by PCR analysis. **C** Relative mRNA level of B7-1 in cortical kidney tissue from Tg mice and wildtype (WT) mice at 2-month-old age. **P < 0.01 versus WT mice. *n* = 5. **D** Representative micrographs confirming the specific expression of B7-1 in podocytes through IF staining with anti-Flag antibody. Arrow indicates positive staining. Bar = 10 μm. **E**–**G** Western blot and quantification of B7-1 and Flag at different age (2-, 3- and 6-months-old). 2-month-old WT mice serve as control group. **P < 0.01 versus controls. *n* = 5. **H** Graphic presentation showing the comparison of Ualb in Tg and WT mice. ***P < 0.001 versus 2-month-old Tg mice; ^#^*P* < 0.05, ^##^*P* < 0.01 versus WT mice at the same age. *n* = 5. **I** Quantitative analysis of the fraction of mesangial area by periodic acid-Schiff (PAS) staining in Tg and WT group at different age. **P < 0.01 versus 2-month-old Tg mice; ^##^*P* < 0.01 versus WT mice at the same age. *n* = 5. **J**, **K** Western blot and quantification of Nephrin, Podocalyxin and Synaptopodin in indicated groups. 2-month-old WT mice serve as control group. *P < 0.05, **P < 0.01 versus controls. *n* = 5. **L** Representative images showing the podocyte ultrastructure and morphology of glomerular basement membrane by TEM micrograph (Arrow indicates foot processes fusion, Bar = 1 μm), Nephrin staining (Arrow indicates the weak signal, Bar = 10 μm), or PAS staining (Arrow indicates mesangial expansion, Bar = 10 μm) in Tg and WT mice at 6-month of age. **M** Quantitative transcriptome analysis of kidney from WT and Tg mice at six months of age. Podocyte**-**expressed genes and the genes involved in β-catenin destruction cascade were shown. **N** Gene set enrichment analysis (GSEA) showing the deactivation of β-catenin transactivating complex signaling were downregulated in 6-month-old Tg mice. **O** Representative micrographs showing the co-expression of β-catenin and Flag-tagged B7-1 in podocytes. Arrows indicate the nuclear expression of β-catenin in podocyte. Bar = 25 μm. **P**–**R** Western blot and quantification of active β-catenin and β-catenin in indicated groups. 2*-*month**-**old WT mice serve as control group. **P < 0.01 versus controls. *n* = 5. **S** Representative micrographs showing the co-staining of β-catenin and Podocalyxin in Tg mice. Arrows i*n*dicate the positive staining. Bar = 25 μm. The full length original western blots for all results are provided in Supplementary File 1.

We generated podocyte-specific B7-1 transgenic (Tg) mice using PiggyBac-induced DNA (a plasmid with NPHS2 promoter-triggered B7-1-3xFlag) microinjection (Fig. 2A). Successful construction of Tg mice or wild type (WT) was identified by PCR (Fig. 2B, C) and Flag staining (Fig. 2D). We analyzed the expression of B7-1 and Flag-tag in Tg mice at different ages. As shown, B7-1 and Flag were both gradually increased with age (Fig. 2E–G, Supplementary Fig. S2). There was no difference in body weight and blood urea nitrogen between WT and Tg mice at the same age, while there was a significant increase in serum creatinine in Tg mice at 6 months of age compared to WT controls (Supplementary Fig. S2).

Albuminuria excretion and glomerular injury of mesangial expansion were then assessed. As shown in Fig. 2H, I, compared to extremely little changes in WT mice at different ages, Tg mice exhibited significantly increased albuminuria and glomerular injury at 3 months old, and those further elevated at six months old. In addition, the expression of Nephrin, Synaptopodin, and Podocalyxin, the epithelial markers of podocyte, showed the decreasing trend with age in Tg mice (Fig. 2J, K). Similarly, as shown in Fig. 2L and Supplementary Fig. S2, podocyte foot processes were irregularly shaped and showed a mild fusion in 3-month-old Tg mice, while there was more disarrangement and partial fusion of podocyte foot processes in 6-month-old Tg mice. Furthermore, Nephrin decreased at 3 months of age in Tg mice, and it was evident at 6 month’s. Periodic acid-Schiff (PAS) staining also showed a slight increase in mesangial expansion and matrix deposition in 3-month-old Tg mice when compared to the 2-month-old Tg mice, and a moderate expansion of mesangium in Tg mice at 6-month old. Whereas, the podocyte epithelial markers, B7-1, active β-catenin, β-catenin, and its ultrastructure remained unchanged in WT mice at 2 to 6 months of age (Supplementary Fig. S2). All these results indicated there would be an amplified transmission process in B7-1 signaling, which mediates the development of podocyte injury.

To explore the underlying mechanisms, we performed transcriptomic analysis. Heatmap and gene ontology (GO) enrichment analysis of the differentially expressed transcripts showed cell adhesion and differentiation, actin cytoskeleton organization, and GBM development were downregulated in Tg mice; whereas the activation of T cell immune response were upregulated (Fig. 2M, Supplementary Fig. S2). As WT1 is a podocyte-sepcific transcription factor, which plays a crucial role in glomerular differentiation and podocyte function, we observed its expression. As shown in Fig. 2M and Supplementary Fig. S2, WT1 was downregulated in Tg mice, accompanied by decrease in a series of podocyte epithelial markers such as Nephrin, Podocin and Synaptopodin. We next performed Gene set enrichment analysis (GSEA). As shown in Fig. 2N, β-catenin pathway, a key player in podocyte injury and glomerular sclerosis [33, 40], was enriched to be activated in Tg mice when compared to WT mice at 6-month-old age.

We then performed co-staining of Flag-B7-1 and β-catenin. Compared to WT mice (Supplementary Fig. S2), they were highly co-expressed in Tg mice (Fig. 2O). We also observed the nuclear expression of β-catenin in Tg mice (Fig. 2O), suggesting its activation. Western blotting analysis of active β-catenin and β-catenin further demonstrated their upregulation in Tg mice (Fig. 2P–R). We then performed the costaining of β-catenin and Podocalyxin, and found they were largely colocalized in Tg mice (Fig. 2S). These data suggest that B7-1 plays a key role in podocyte injury via β-catenin activation.

### Knockdown of B7-1 protects against podocyte injury and glomerular damage through inhibiting β-catenin

**Fig. 3:**
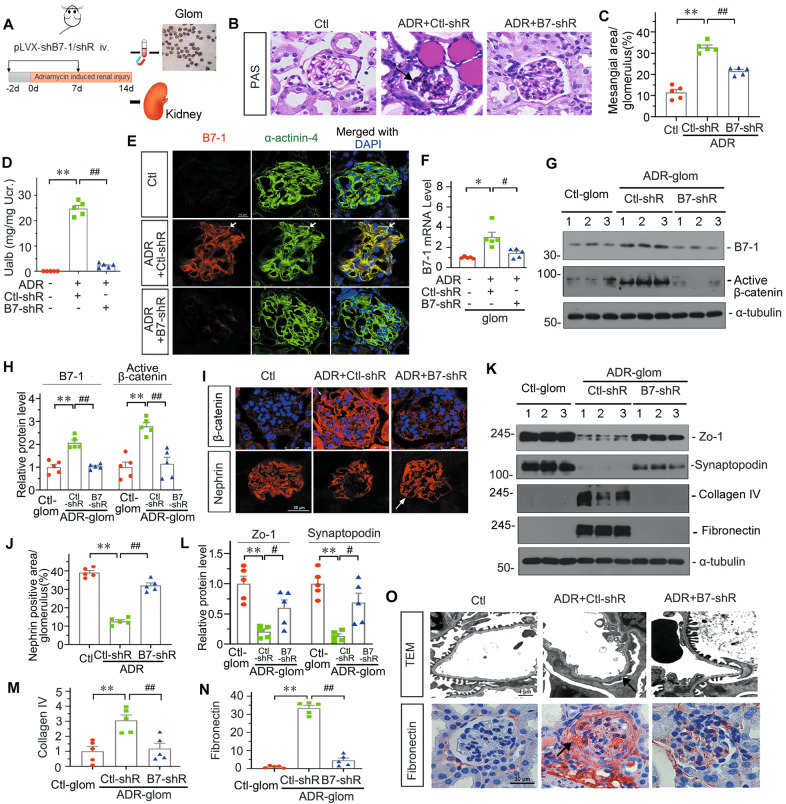
Knockdown of B7-1 protects against podocyte injury and glomerular damage through inhibiting β-catenin. **A** Experimental design. Arrows indicate injections of shRNA vectors carrying B7-1 interference sequence (pLVX-shB7-1) or negative control at indicated time points. Mice were intravenously injected with Adriamycin, and sacrificed 14 days after ADR injection for isolation of glomeruli or harvesting kidney tissue. **B**, **C** Representative micrographs and quantifications showing the fraction of mesangial area by PAS staining in indicated groups. Bar = 30 μm. **P < 0.01 versus control (Ctl) group. ^##^*P* < 0.01 versus mice treated with ADR and control shRNA vector (ADR + Ctl-shR). *n* = 5. **D** Graphic presentation showing Ualb in different groups. **P < 0.01 versus Ctl group; ^##^*P* < 0.01 versus ADR + Ctl-shR group^.^ *n* = 5. **E** Representative micrographs showing the colocalization of B7-1 and α-actinin-4 in indicated group. Arrows indicated specific staining in podocyte. Bar = 10 μm. **F** Graphic presentations show the relative mRNA levels of B7-1 in isolated glomeruli from different groups. *P < 0.05 versus Ctl group; ^#^*P* < 0.05 versus ADR+Ctl-shR group. *n* = 5. **G**, **H** Western blot and quantification of B7-1 and active β-catenin in isolated glomerulus from different groups. **P < 0.01 versus Ctl group; ^##^*P* < 0.01 versus ADR + Ctl-shR group. *n* = 5. **I** Representative micrographs showing the expression of β-catenin and Nephrin in indicated groups. Arrow indicates positive staining. Bar = 25 or 30 μm. **J** Quantitative measurement of Nephrin positive area / glomerulus area by IF staining. **P < 0.01 versus Ctl group; ^##^*P* < 0.01 versus ADR + Ctl-shR group. *n* = 5. **K** Representative Western blotting of glomerular Zo-1, Synaptopodin, Collagen IV and Fibronectin in isolated glomerulus from different groups. **L–N** Quantitative data showing the relative protein levels by Western blot. **P < 0.01 versus Ctl group; ^#^*P* < 0.05, ^##^*P* < 0.01 versus ADR + Ctl-shR group. *n* = 5. **O** Representative images showing podocyte ultrastructure by TEM micrographs (Arrow indicates foot process fusion, Bar = 1 μm), or Fibronectin staining (Arrow indicates positive staining, Bar = 30 μm) in indicated groups. The full length original western blots for all results are provided in Supplementary File 1.

To knock down B7-1 in ADR mice, we injected an shRNA vector encoding the interference sequence for B7-1 (pLVX-shB7-1) by a hydrodynamic approach [40]. The interference efficiency of B7-1 was first testified in several organs. It showed that B7-1 was successfully interferred in kidney and liver (Supplementary Fig. S3). PAS staining showed that B7-1 knockdown strongly alleviated glomerular injury (Fig. 3B, C). The leakage of albuminuria was also significantly decreased by B7-1 knockdown (Fig. 3D).

We next performed costaining. As shown in Fig. 3E, B7–1 was upregulated in ADR mice and colocalized with α-actinin-4. We then assessed the relationship of B7-1 and β-catenin in isolated glomeruli from three groups of mice. As shown in Fig. 3F–H, B7-1 mRNA and protein were upregulated in ADR-glom, but inhibited by B7-1 knockdown. Furthermore, active β-catenin and β-catenin expression showed the same trend as B7-1 (Fig. 3G–I), suggesting the intimate correlation between them.

We then assessed podocyte injury and fibrosis. As shown in Fig. 3I–L, the expression of Nephrin, Zo-1, and Synaptopodin were decreased in ADR mice, but largely restored by B7-1 knockdown. TEM analysis also revealed that B7-1 knockdown could effectively maintain the integrity of podocyte foot processes (Fig. 3O). Furthermore, B7-1 knockdown in ADR mice strongly blocked the expression of collagen IV and Fibronectin, the fibrogenesis markers (Fig. 3K, M–O). We also knocked down B7-1 in 5/6 nephrectomy mice, a model of chronic renal failure with podocyte injury [42], and found the similar results (Supplementary Fig. S4). These results further suggest B7-1 plays an important role in podocyte injury through β-catenin signaling.

### B7-1 mediates podocyte injury through activating β-catenin signaling in vitro and in glomerular mini-organ culture

**Fig. 4:**
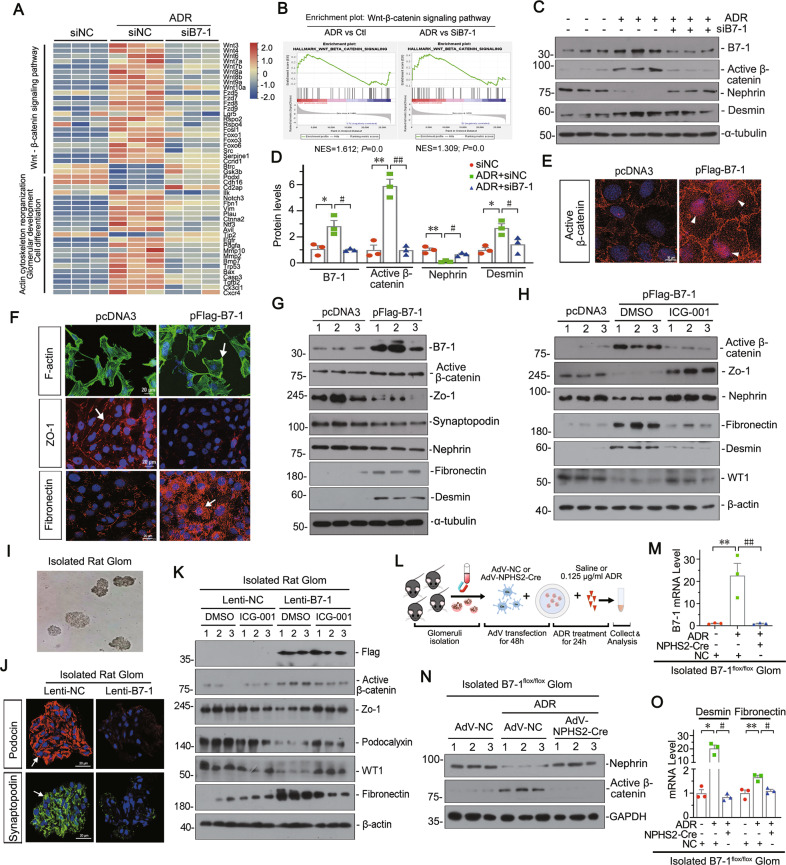
B7-1 mediates podocyte injury through activating β-catenin signaling in vitro and glomerular mini-organ culture. **A** Heatmap plot of transcriptomic analysis showing the changes in the Wnt/β-catenin signaling, cell differentiation, actin cytoskeleton reorganization and glomerular development at different groups. *n* = 3. MPC5 cells were transfected with B7-1 siRNA(siB7-1) or negative control(si-NC) for 24 h, following 0.25 μg/ml of ADR treatment for another 24 hours. **B** GSEA analysis of different comparisons among groups show ADR induced genes enriched in Wnt/β-catenin pathway, and blocked by B7-1 interference. Ctl: siNC group; ADR: ADR + siNC group; SiB7-1: ADR + siB7-1 group. *n* = 3. **C**, **D** Western blot and quantification of B7-1, active β-catenin, Nephrin and Desmin in different groups as indicated. *P < 0.05, **P < 0.01 versus siNC group. ^#^*P* < 0.05, ^##^*P* < 0.01 versus ADR + siNC group. *n* = 3. **E**, **F** Representative images showing the expression of active β-catenin, F-actin, Zo-1 and Fibronectin in MPC5 cells. Cells were transfected with B7-1 expressing plasmid (pFlag-B7-1) or empty vector (pcDNA) for 24 hours. Bar = 10 or 20 μm. **G** Western blot showing the protein expression of B7-1, active β-catenin, Zo-1, Synaptopodin, Nephrin, Fibronectin and Desmin in indicated groups. *n* = 3. **H** Western blot showing the expression of active β-catenin, Zo-1, Nephrin, Fibronectin, Desmin and WT-1. MPC5 cells were pre-treated with ICG-001 (5 μmol/L) for one hour, following transfection with pFlag-B7-1 plasmid (or pcDNA) for another 24 hours. *n* = 3. **I** Representative image showing the isolated rat glomeruli under light microscopy. **J** Representative images showing staining of Podocin and Synaptopodin in isolated rat glomeruli treated with B7-1 lentivirus (Lenti-B7-1) or negative control lentivirus (Lenti-NC). Bar = 20 μm**. K** Western blot showing the expression of Flag tag, active β-catenin, Zo-1, Podocalyxin, WT1, and Fibronectin in isolated rat glomeruli treated with indicated treatment. The glomeruli were first pre-treated with ICG-001 (5 μmol/L) or DMSO, following infection with B7-1 expressing lentivirus or negative control for another 72 hours. *n* = 3. **L** Experimental scheme showing the isolated glomeruli from B7-1^flox/flox^ mice treated with adenovirus expressing NPHS2-cre recombinase to knockdown B7-1 in podocytes and co-treated with ADR. The mouse glomeruli were isolated by magnetic beads and plated on the six-well plates, treated with the indicated adenovirus for 48 hours and followed by ADR (0.125 μg/ml) treatment for another 24 hours. **M** Graph showing the mRNA level of B7-1. **P < 0.01 versus negative control group (AdV-NC). ^##^*P* < 0.01 versus ADR group (AdV-NC + ADR). *n* = 3. **N** Western blot showing the expression of Nephrin and active β-catenin in isolated B7-1^flox/flox^ mouse glomeruli. **O** Graphs showing the mRNA levels of Desmin and Fibronectin in isolated B7-1^flox/flox^ mouse glomeruli. *P < 0.05, **P < 0.01 versus negative AdV-NC group. ^#^*P* < 0.05 versus AdV-NC + ADR group. *n* = 3.ADR Adriamycin, DMSO Dimethyl sulfoxide. The full length original western blots for all results are provided in Supplementary File 1.

We further assessed the role of B7-1 in podocyte injury in cultured mouse podocyte cell line (MPC5) and glomerular mini-organ culture. MPC5 cells were firstly transfected with B7-1 siRNA and co-treated with ADR. Transcriptomic and heatmap analysis showed ADR downregulated epithelial cell differentiation and triggered the activation of Wnt/β-catenin signaling, along with the increase in cell apoptosis (Fig. 4A, Supplementary Fig. S8). GSEA further clarified β-catenin pathway was highly related with B7-1 signaling (Fig. 4B). Western blotting analysis (Fig. 4C, D) showed active β-catenin was upregulated in ADR-treated cells, but greatly inhibited by B7-1 knockdown, which was accompanied by the restoration of Nephrin and decrease in Desmin, a podocyte injury marker.

MPC5 cells were then transfected with B7-1 expressing plasmid. Ectopic B7-1 induced the increase in active β-catenin, and promoted its translocation into the nuclei (Fig. 4E). Concomitantly, B7-1 overexpression triggered the actin skeleton rearrangement, loss of epithelial properties, and epithelial to mesenchymal transition (EMT) in podocytes, as assessed by F-actin, Zo-1 and Fibronectin staining (Fig. 4F), and western blotting analysis of other podocyte epithelial or EMT markers (Fig. 4G, Supplementary Fig. S5). Furthermore, pretreatment with ICG-001, an inhibitor of β-catenin activation, could greatly inhibit B7-induced those effects (Fig. 4H, Supplementary Fig. S5).

We further testify the role of B7-1 in rat glomerular mini-organ culture (Fig. 4I). As shown in Fig. 4J, K, and Supplementary Fig. S5, transduction of lentivirus expressing B7-1 gene triggered the decrease in Podocin, Synaptopodin, Zo-1, Podocalyxin, and WT1, but increased the expression of active β-catenin and Fibronectin. However, ICG-001 could greatly inhibit these effects.

To deeply identify the function of B7-1 in podocyte and glomerular injury, we established B7-1^flox/flox^ mice (Supplementary Fig. S5). To specifically knockout B7-1 in podocytes, we isolated glomeruli from B7-1^flox/flox^ mice and transduced them with adenovirus encoding NPHS2-driving Cre recombinase (Fig. 4L, M). After that, the glomerular mini-organ culture was treated with ADR. As shown in Fig. 4M–O, and Supplementary Fig. S5, podocyte specific knockout of B7-1 could significantly restore the expression of Nephrin, decreased the expression of active β-catenin, Desmin, and Fibronectin. These data further suggest B7-1 plays a key role in podocyte injury via β-catenin signaling.

### Hsp90ab1 plays a key role in signal transmission from B7-1 to the LRP5/β-catenin pathway

**Fig. 5:**
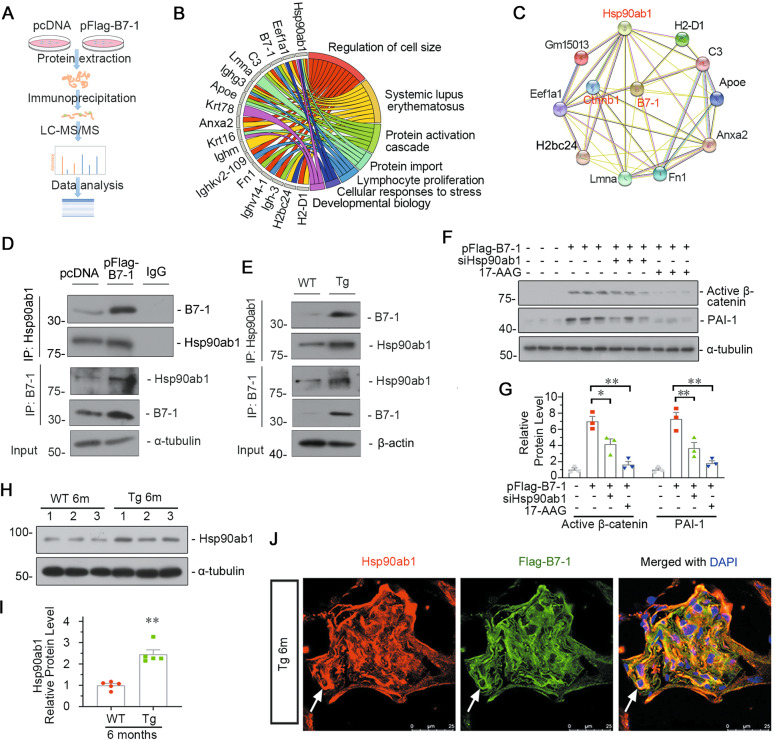
Hsp90ab1 plays a key role in B7-induced podocyte injury. **A** Diagram showing the procedure using the IP-MS/MS proteomics to identify the specific proteins interacted with B7-1. **B** Chord plot showing GO enrichment based on B7-1-interacted genes detected in B7-1-overexpressed podocytes. **C** STRING analysis showing the protein-protein interaction network formed by B7-1-interacted proteins and β-catenin (CTNNB1). **D**, **E** Representative graphs showing the binding of B7-1 with Hsp90ab1 in vitro and in vivo. MPC5 cells were transfected with B7-1 expression plasmid for 24 h. Kidney tissues samples were harvested from 6-month-old WT or Tg mice. The protein lysates were immunoprecipitated with an antibody against B7-1, Hsp90ab1 or rabbit IgG, respectively, and blotted with the antibody against B7-1 or Hsp90ab1. Total diluted lysates were used as input. Experiments were repeated three times. **F**, **G** Western blot and quantitative data showing the protein expression of active β-catenin and PAI-1. MPC5 cells were pre-treated with Hsp90ab1 siRNA or 17-AAG (1 μmol/L), following pFlag-B7-1 plasmid (or pcDNA) transfection. *P < 0.05, **P < 0.01 versus B7-1 overexpression group. *n* = 3. **H**, **I** Representative Western blot and quantitative data showing the expression of Hsp90ab1 in 6-month-old WT and Tg mice. **P < 0.01 versus WT mice^.^ **J** Representative micrographs showing the co-expression of Hsp90ab1 and Flag-tag (B7-1) in Tg mice. Arrows indicate the colocalization in podocytes. Bar = 25 μm. The full length original western blots for all results are provided in Supplementary File 1.

We tried to find the protein binding to B7-1. Through immunoprecipitation and liquid chromatography-mass spectrometry (LC-MS), we found nearly 20 proteins binding to B7-1. We then performed GO enrichment analyses using B7-1-specific-binding partners (Fig. 5B). As shown, B7-1 was primarily involved in immune-related responses and cell homeostasis-related pathways. Notably, in B7-1-specific-binding partners, heat shock protein 90 alpha, class B member 1 (Hsp90ab1), a conserved molecular chaperone, contributed to most of these enriched pathways [43]. STRING analysis of the protein-protein interaction network with β-catenin indicated that B7-1 linked to β-catenin through interacting with Hsp90ab1 (Fig. 5C). To verify it, we conducted co-immunoprecipitation (Co-IP) experiments in B7-1-overexpressed cells and podocyte-specific B7-1 Tg mice (Fig. 5D, E). As shown, B7-1 could perfectly bind with Hsp90ab1. MPC5 cells were pretreated with 17-allylaminogeldanammycin (17-AAG, a type of Hsp90 inhibitor targeting the N-terminal domain (NTD)), or transfected with siRNA to Hsp90ab1 NTD domain, an important region involving in modulating chaperone function. As shown in Fig. 5F, G, the interruption of Hsp90ab1 significantly inhibited the activation of β-catenin and the expression of its target PAI-1 in B7-1-overexpressed podocytes. We then assessed the expression of Hsp90ab1 in Tg mice. As shown in Fig. 5H, I, the expression of Hsp90ab1 was significantly induced in B7-1-Tg mice. The co-staining of Hsp90ab1 with Flag (the tag of podocyte B7-1) showed that Hsp90ab1 was increased in Tg mice and largely co-localized with B7-1 (Fig. 5J), compared with the weak expression in WT mice (Supplementary Fig. S2).

**Fig. 6:**
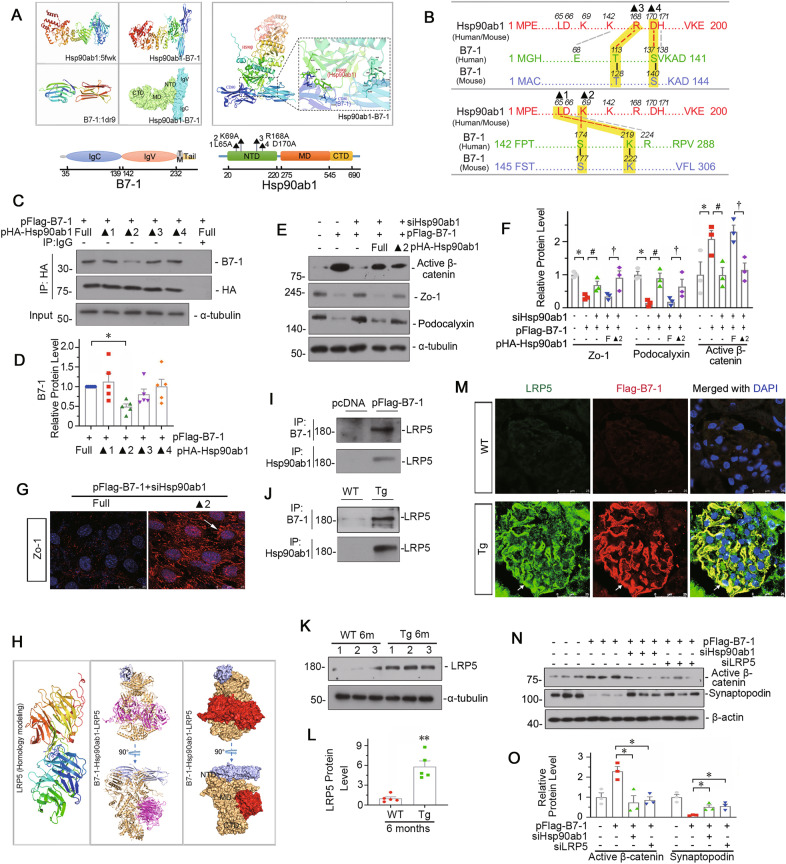
Hsp90ab1 mediates the signal transmission from B7-1 to the LRP5/β-catenin pathway. **A**, **B** Graphic presentation showing the predicted binding sites between B7-1 and Hsp90ab1 residues L65 (▴1), K69 (▴2), R168 (▴3), D170 (▴4). The full structure of B7-1 contains IgC domain, IgV domain, transmembrane domain (TM) and Tail. The full structure of Hsp90ab1 contains N-terminal domain (NTD), middle domain (MD), C-terminal domain (CTD), and marked with ▴ for specific point mutation. **C**, **D** Representative immunoblotting and quantitative data showing the specific binding site in Hsp90ab1 for interaction with B7-1 is ▴2 (K69). 293 T cells were transfected with B7-1 expression plasmid (Flag tagged), and co-transfected with HA tagged Hsp90ab1 wildtype plasmid (Full) or mutation plasmids for 24 hours. Whole cell lysates were immunoprecipitated with a HA-tag antibody and immunoblotted with B7-1 or HA antibody. Total diluted lysates were used as input. Each dot on the bar plot indicated an individual repeated experiment run in duplicate. *P < 0.05 versus the wildtype group. **E**, **F** Representative Western blot and quantitative data of active β-catenin, Zo-1 and Podocalyxin (podx) in indicated group. MPC5 cells were transfected with siHsp90ab1 for 24 h, followed by transfection with pFlag-B7-1 plasmid and Hsp90ab1 wildtype plasmid (Full) or K69A mutated (▴2) plasmid for another 24 hours. *P < 0.05 versus controls, ^#^*P* < 0.05 versus pFlag-B7-1 group, ^†^*P* < 0.05versus cells co-transfected with Hsp90ab1 wild type plasmid and pFlag-B7-1 plasmid. **G** Micrographs showing the expression of Zo-1 in MPC5 cells pretreated with siHsp90ab1, and co-transfected with pFlag-B7-1 and wild type plasmid of Hsp90ab1 (Full) or mutated plasmid (▴2). Arrow indicates the positive staining. Bar = 25 μm. **H** Molecular structure of LRP5 from homology modeling and putative binding model of B7-1, LRP5 and Hsp90ab1 dimer. **I**, **J** Representative immunoblotting show the interaction between LRP5 and B7-1 or Hsp90ab1 in cells or kidney tissue lysates as indicated. The protein lysates were immunoprecipitated with antibodies against B7-1 and Hsp90ab1 respectively, and blotted with anti-LRP5. **K**, **L** Western blot and quantification of LRP5 in 6-month-old WT and Tg mice. **P < 0.01 versus WT group. *n* = 5. **M** Representative micrographs showing the expression of LRP5 and Flag-tag (B7-1) in WT and Tg mice. Arrows indicate the specific colocalization in podocytes. Bar = 25 μm. **N**, **O** Western blot and quantification of active β-catenin and Synaptopodin. MPC5 cells were transfected with pFlag-B7-1 plasmid (or empty vector), and co-transfected with siRNA to Hsp90ab1 (siHsp90ab1) or LRP5 (siLRP5). *P < 0.05, **P < 0.01 versus B7-1 overexpression group. *n* = 3^.^ The full length original western blots for all results are provided in Supplementary File 1.

We then performed molecular docking of the conserved B7-1 protein sequence (containing IgC and IgV domains) and the full-length protein of Hsp90ab1 (highly homologous in evolution) using Discovery Studio. The best-predicted conformations are shown in Fig. 6A. BLAST analysis was performed to determine the conservative binding sites between Hsp90ab1 and B7-1 protein sequences. The results showed that the residues L65, K69, R168, and D170 in NTD domain of Hsp90ab1 could serve as the key sites binding with B7-1 (Fig. 6B). Subsequently, these 4 residues in Hsp90ab1 were mutated to alanine individually to construct mutated plasmids. An immunoprecipitation (IP) assay was performed in 293 T cells. As shown in Fig. 6C, D, compared with the full binding activity of wild-type Hsp90ab1 (Full) with B7-1, the Hsp90ab1 with a K69 residue mutation (▴2) significantly decreased its binding activity with B7-1. The other residues mutations (▴1, 3, 4) showed no effects. These suggested that K69 residue in Hsp90ab1 NTD domain might be the key binding site interacting with B7-1. To verify its role, MPC5 cells were first transfected with siRNA to silence the endogenous Hsp90ab1, and then transfected with B7-1 expressing plasmid and wild-type or K69-mutated Hsp90ab1 expressing plasmid. As shown in Fig. 6E, F, compared to wild-type (Full), a mutation at K69 residue (▴2) in Hsp90ab1 significantly repressed B7-1-induced expression of active β-catenin. Furthermore, the expression of Zo-1 and Podocalyxin was significantly restored by K69 mutation (▴2) in Hsp90ab1. Similar results were observed when Zo-1 was assessed by immunofluorescence (Fig. 6G).

It was reported that Hsp90ab1 could interact with LRP5 [44], an indispensable coreceptor of the Wnt/β-catenin signaling pathway [34]. Therefore, we investigated the role of LRP5 in the B7-1/Hsp90ab1-induced β-catenin pathway. Considering that no LRP5 crystal structures have been published, we built the structure of the LRP5 protein from homologous models according to its conserved sequences. We then conducted docking analysis of B7-1, LRP5 and the functional dimer of Hsp90ab1, and determined the optimal conformation. They showed a perfect binding (Fig. 6H). The binding of B7-1, LRP5, and Hsp90ab1 was then demonstrated in B7-1-overexpressed MPC5 cells and podocyte-specific B7-1 Tg mice (Fig. 6I, J). The upregulation of LRP5 was also testified in Tg mice (Fig. 6K, L), and further identified to be colocalized with B7-1 in podocytes (Fig. 6M). The upregulation of LRP5 was also demonstrated in clinical nephropathy, and verified to be located in podocytes (Supplementary Fig. S7). In MPC5 cells, interference of LRP5 using siRNA transfection significantly blocked B7-1-induced activation of β-catenin and restored the expression of Synaptopodin (Fig. 6N, O). These results suggest that B7-1 could induce the activation of the LRP5/β-catenin pathway, and HspP90ab1, especially the K69 residue, plays a key role in signal transmission.

Consistent with the previous report [31], we also observed integrin β1 signaling was disrupted by B7-1 overexpression (Supplementary Fig. S8). Interestingly, our results further demonstrated Hsp90ab1 could also mediate the interaction between integrin β1 and B7-1, suggesting that Hsp90ab1 might also contribute to integrin activation and actin cytoskeleton reorganization (Supplementary Fig. S8).

### Hsp90ab1 contributes to B7-1-induced podocyte injury and glomerular damage

**Fig. 7:**
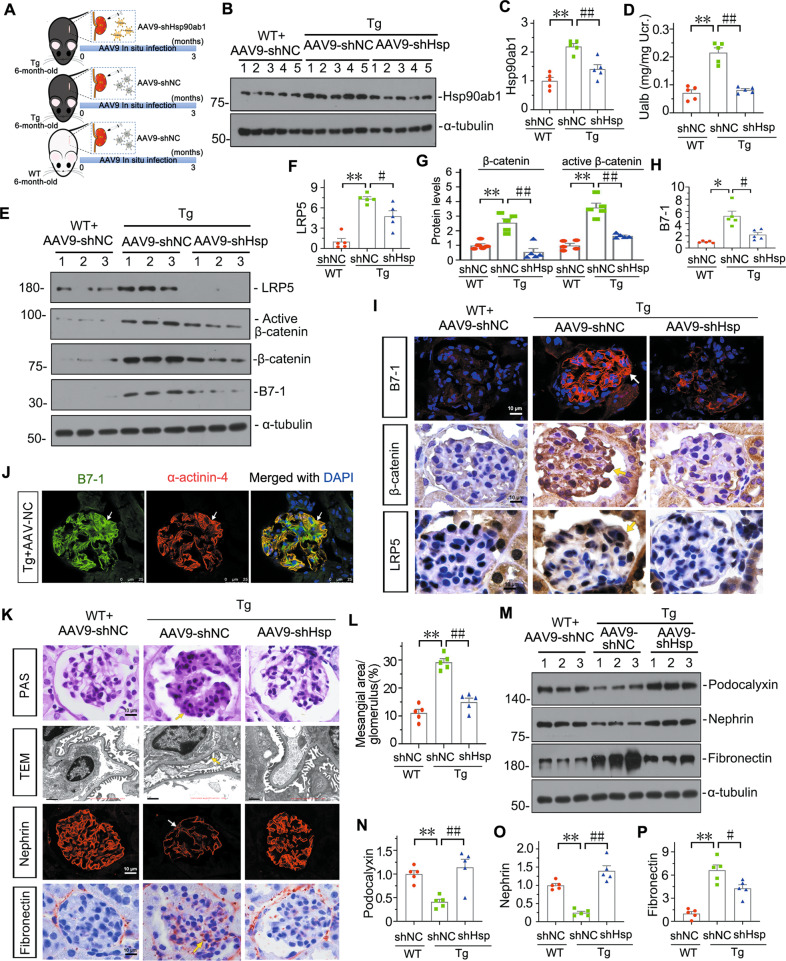
Hsp90ab1 contributes to B7-1-induced podocyte injury and glomerular damage. **A** A schematic diagram showing the procedure of Hsp90ab1 knockdown in Tg mice by in situ infection with adeno-associated virus (AAV9-shHsp90ab1 or negative control (AAV9-shNC)). **B**, **C** Western blot and quantification of Hsp90ab1 in indicated groups. **P < 0.01 versus WT + AAV9-shNC group; ^##^*P* < 0.01 versus Tg+AAV-shNC group. *n* = 5. **D** Graphic prese*n*tation showing Ualb in different groups. **P < 0.01 versus WT + AAV9-shNC group; ^##^*P* < 0.01 versus Tg+AAV-shNC group^.^ *n* = 5. (**E**–**H**) Western blot and quantification of LRP5, active β-catenin, β-catenin and B7-1 in indicated groups. ^*^P < 0.05, **P < 0.01 versus WT + AAV9-shNC group^; #^*P* < 0.05, ^##^*P* < 0.01 versus Tg+AAV-shNC group. *n* = 5. **I** Representative micrographs showing the expression of B7-1, β-catenin and LRP5 in different groups. Arrows indicate positive staining. Bar = 10 μm. **J** Representative micrographs showing the co-expression of B7-1 and α-actinin-4. Arrows indicate specific co-localization in podocyte. Bar = 25 μm. **K** Representative images showing the typical PAS staining of glomerular structure (Bar = 10 μm), podocyte ultrastructure by TEM micrograph (Yellow arrow indicates foot process fusion, Bar = 1 μm), Nephrin staining (Arrow indicates the weak signal in podocyte, Bar = 10 μm), and Fibronectin staining (Yellow arrow indicates positive staining, Bar = 10 μm). **L** Graph showing quantitative analysis of the fraction of mesangial area by PAS staining. **P < 0.01 versus WT + AAV9-shNC group. ^##^*P* < 0.01 versus Tg+AAV-shNC group. *n* = 5. **M**–**P** Representative Western blot and quantitative data showing protein expression of Podocalyxin, Nephrin and Fibronectin in indicated groups. **P < 0.01 versus WT + AAV9-shNC group. ^#^*P* < 0.05, ^##^*P* < 0.01 versus Tg+AAV-shNC group. *n* = 5. The full length original western blots for all results are provided in Supplementary File 1.

To further demonstrate the role of Hsp90ab1 in B7-1 signaling, we treated podocyte-specific B7-1 transgenic (Tg) mice with adeno-associated virus (AAV) expressing the interference sequence of Hsp90ab1(AAV9-shHsp90ab1) using in situ injection to kidney (Fig. 7A). The 6-month-old Tg mice were injected with AAV9-shHsp90ab1 or control virus for three months (Fig. 7A). As shown in Fig. 7B, Hsp90ab1was increased in Tg mice at nine months old compared to WT mice, but this was blocked by AAV9-shHsp90ab1 injection. Furthermore, knockdown of Hsp90ab1 in Tg mice also inhibited the excretion of albuminuria (Fig. 7D). Western blotting analysis and immunostaining showed Hsp90ab1 knockdown inhibited the expression of LRP5, active β-catenin and β-catenin, and B7-1 (Fig. 7E–I). The expression of B7-1 in podocytes was also confirmed by costaining with α-actinin-4 (Fig. 7J).

We next analyzed glomerular injury. As shown in Fig. 7K, L, glomerular mesangium expansion was upregulated in Tg mice, but this was inhibited by Hsp90ab1 knockdown. Similarly, TEM analysis, immunostaining, and western blotting further identified podocyte injury and glomerular fibrotic changes were increased in Tg mice, but blocked by Hsp90ab1 knockdown (Fig. 7K–P).

### Hsp90ab1 mediates B7-1-induced podocyte injury and glomerular damage in ADR mice

**Fig. 8:**
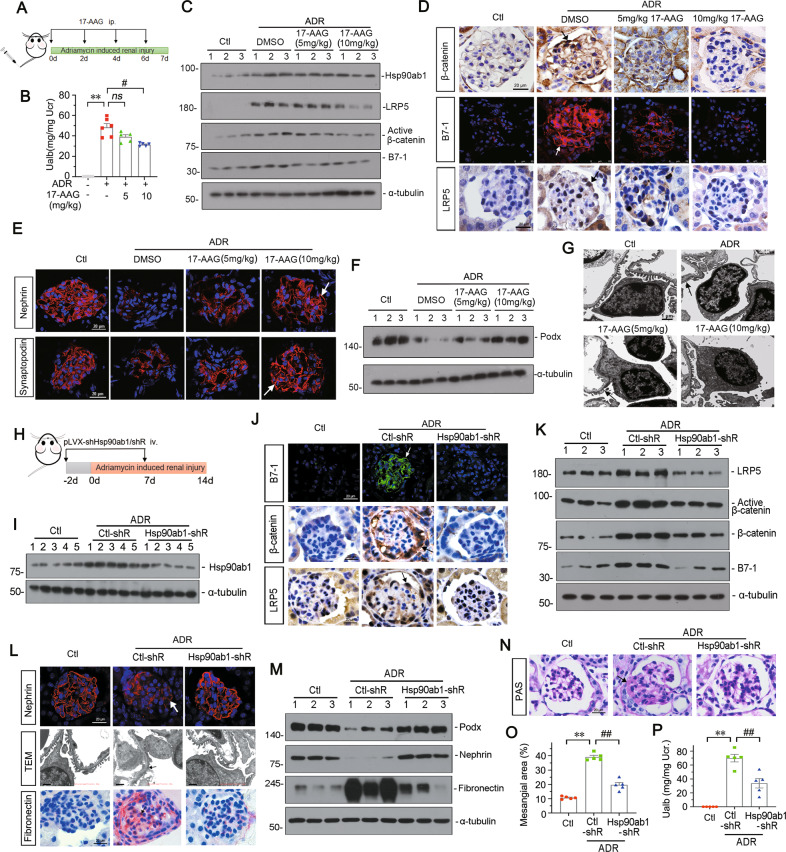
Hsp90ab1 plays an important role in mediating B7-1-induced podocyte and glomerular injury. **A** Experimental design. Arrows indicate intravenous injections of 5 mg/kg or 10 mg/kg of 17-AAG at indicated time points. Mice were sacrificed seven days after ADR injection. **B** Graphic presentation showing Ualb in different groups. **P < 0.01 versus controls. ns=no significance, ^##^*P* < 0.01 versus ADR group. *n* = 5. **C** Representative Western blot showing protein expression of Hsp90ab1, LRP5, active β-catenin and B7-1 in indicated groups. *n* = 5. **D** Representative micrographs showing the expression of β-catenin, B7-1 and LRP5 in different groups. Arrows indicate positive staining. Bar = 20 or 25 μm. **E** Representative micrographs showing the expression of Nephrin and Synaptopodin in different groups. Arrows indicate positive staining. Bar = 20 μm. **(F)** Representative western blot showing protein expression of Podocalyxin in indicated groups. *n* = 5. **G** Representative TEM micrographs showing podocyte ultrastructure. Arrows indicates the fusion of foot processes. Bar = 1 μm. **H** Experimental design. Arrows indicate intravenous injections of Ctl-shR or Hsp90ab1-shR at indicated time points. Mice were sacrificed 14 days after ADR injection. **I** Representative Western blot showing protein expression of Hsp90ab1 in indicated groups. *n* = 5. **J** Representative micrographs showing the expression of B7-1, β-catenin, and LRP5 in different groups. Arrows indicate positive staining. Bar = 20 μm. **K** Representative Western blot showing protein expression of LRP5, active β-catenin, β-catenin and B7-1 in indicated groups. *n* = 5. **L** Representative images showing IF staining of Nephrin (Arrow indicates weak signal in podocyte. Bar = 20 μm), podocyte ultrastructure by TEM micrograph (Arrow indicates the fusion of foot processes. Bar = 1 μm), or IHC staining of Fibronectin (Arrow indicates positive staining. Bar = 20 μm) **M** Representative western blot showing protein expression of Podocalyxin (Podx), Nephrin, and Fibronectin in indicated groups. *n* = 5. **N**, **O** Representative micrographs and quantification showing glomerular structure changes by PAS staining in indicated groups. Bar = 20 μm. **P < 0.01 versus control group. ^##^*P* < 0.01 versus ADR + Ctl-shR group^.^ *n* = 5. **P** Graphic presentation showing Ualb in different groups. **P < 0.01 versus control group. ^##^*P* < 0.01 versus ADR + Ctl-shR group. *n* = 5. The full length original western blots for all results are provided in Supplementary File 1.

To further clarify the role of Hsp90ab1 in B7-1 signaling, ADR-treated mice were intraperitoneally injected with 17-AAG (Fig. 8A). As shown, 17-AAG dose-dependently decreased the excretion of urinary albumin (Fig. 8B), and significantly inhibited the expression of Hsp90ab1, LRP5, active β-catenin, and B7-1 (Fig. 8C, Supplementary Fig. S6). Their expression was also tested by immunostaining (Fig. 8D). The colocalization of B7-1 with α-actinin-4 was also detected in ADR mice (Supplementary Fig. S6). Furthermore, we found 17-AAG could significantly restore the expression of Nephrin, Synaptopodin and Podocalyxin, and protect the ultrastructure of podocyte foot processes (Fig. 8G and Supplementary Fig. S6).

We further checked the effects of Hsp90ab1 interference in the long-term model of ADR mice. Knockdown of Hsp90ab1 was performed by injection of shRNA to Hsp90ab1 (pLVX-shHsp90ab1) using hydrodynamic approach (Fig. 8H, I). As shown in Fig. 8J, K, Hsp90ab1 knockdown could interrupt the upregulation of B7-1, β-catenin, active β-catenin, and LRP5 in ADR mice. Furthermore, Hsp90ab1 knockdown greatly restored the expression of Nephrin and Podocalyxin, and the normal ultrastructure of podocyte foot processes, but decreased the expression of Fibronectin and glomerular mesangium expansion (Fig. 8L–P). Hsp90ab1 knockdown also decreased the leakage of albuminuria (Fig. 8P). These results further suggest Hsp90ab1 mediates B7-1-induced podocyte injury and glomerular damage.

### B7-1 is a downstream target of β-catenin and facilitates a two-way network with β-catenin

**Fig. 9:**
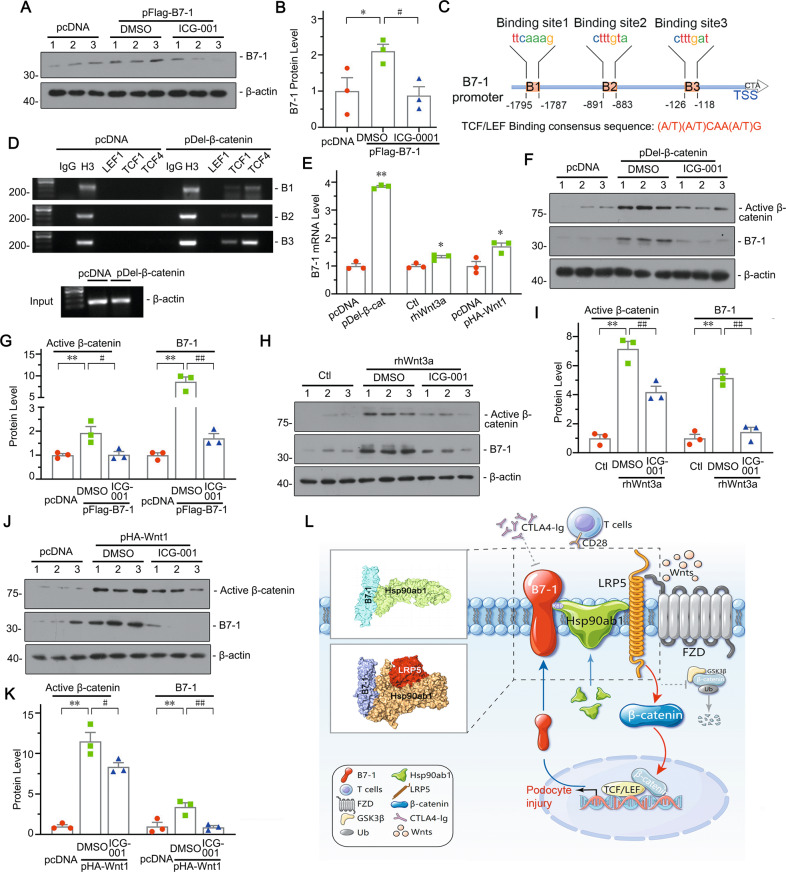
B7-1 is a downstream target of β-catenin and facilitates a two-way network with β-catenin. **A**, **B** Representative western blot and quantification of B7-1 in indicated groups. *P < 0.05 versus pcDNA group. ^#^*P* < 0.05 versus the pFlag-B7-1 group. **C**, **D** Bioinformation analysis and chromatin immunoprecipitation (ChIP) assay reveal the presence of T cell factors/Lymphoid enhancer factor (TCFs/LEF) binding sites in the promoter region of mouse B7-1 gene. The sequences and positions of the putative TCFs/LEF in the B7-1 gene are shown, and the TCFs/LEF consensus sequence is also given. MPC5 cells were transfected with β-catenin expressing plasmid (pDel-β-catenin, Flag tagged) or pcDNA3 for 24 h. Cell DNAs were precipitated with an antibody against LEF1, TCF1, TCF4, histone H3 or nonimmune IgG. PCR assay was performed to detect TCFs/LEF binding consensus sequences in B7-1 gene promoter. Total diluted lysates were used as total genomic input DNA. **E** Graph showing the upregulation of B7-1 mRNA by β-catenin expressing plasmid (pDel-β-catenin) transfection, Wnt3a recombinant protein (rhWnt3a) stimulation, or Wnt1 expressing plasmid (pHA-Wnt1) transfection. *P < 0.05, **P < 0.01 versus controls (pcDNA or medium alone group). **F**, **G** Western blot and quantification of B7-1 and active β-catenin in indicated groups. MPC5 cells were pretreated with ICG-001, and followed by pDel-β-catenin plasmid transfection. **P < 0.01 versus control group. ^#^*P* < 0.05, ^##^*P* < 0.01 versus the pDel-β-catenin group. **H**, **I** Representative western blot and quantification of B7-1 and Active β-catenin in indicated groups. MPC5 cells were pretreated with ICG-001, and followed by rhWnt3a incubation for 24 hours. ^**^P < 0.01 versus control group. ^#^*P* < 0.05, ^##^*P* < 0.01 versus the rhWnt3a group. **J**, **K** Western blot and quantification of B7-1 and active β-catenin in indicated groups. MPC5 cells were pretreated with ICG-001, and followed by pHA-Wnt1 plasmid transfection. ^**^P < 0.01 versus control group. ^#^*P* < 0.05, ^##^*P* < 0.01 versus the pHA-Wnt1 group. **L** Diagram depicting the potential mechanism of B7-1 in podocyte. The full length original western blots for all results are provided in Supplementary File 1.

MPC5 cells were pre-treated with ICG-001 and then transfected with B7-1 expressing plasmid. As shown, ICG-001 reduced B7-1 expression (Fig. 9A, B), suggesting β-catenin plays a key role in B7-1 signaling. Typically, β-catenin forms a complex with members of the T cell factors/lymphoid enhancer binding factor (TCFs/LEF) family, including TCF1/3/4 and LEF1, to regulate downstream gene expression [45]. We found there are several potential binding sites in the B7-1 promoter regions for binding with TCFs/LEF (Fig. 9C). Chromatin immunoprecipitation (ChIP) assay showed ectopic expression of β-catenin promoted TCF1 and TCF4, but not LEF1 to bind to the TCFs/LEF binding consensus sequence regions in B7-1 gene promoter (Fig. 9D) and upregulated B7-1 mRNA levels (Fig. 9E), suggesting direct stimulatory effects of β-catenin on B7-1. Ectopic β-catenin-induced B7-1 was significantly reduced by ICG-001 treatment (Fig. 9F, G). Similarly, overexpression of Wnt1 or exogenous Wnt3a also increased the mRNA and protein expression of B7-1 (Fig. 9E–K), which was inhibited by ICG-001 treatment (Fig. 9H–K). We also cultured HUVEC and RMC cells and transfected them with β-catenin expression plasmid. Although there was a slight increase in B7-1 mRNA in β-catenin-overexpressed HUVEC cells, it was not statistically significant. As for RMC cells, there was no changes (Supplementary Fig. S9). These results further suggest that β-catenin-induced B7-1 could possibly be podocyte-specific.

Collectively, our results suggest that the B7-1/β-catenin pathway appears to create a two-way network through Hsp90ab1, and plays a key role in the pathogenesis of podocyte injury (Fig. 9L). B7-1 recruits Hsp90ab1 to form a complex together with LRP5 at the cell membrane. Hsp90ab1 serves as a chaperon protein for B7-1 and LRP5. The residue K69 in the Hsp90ab1 N terminal domain is the key binding site for B7-1, which facilitates the signal transmission from B7-1 to LRP5/β-catenin signaling activation. β-catenin translocates into nuclei to promote B7-1 expression through TCFs/LEF transcription factors. These form a reciprocal activation feedback loop, and Hsp90ab1, especially residue K69, plays a key role in this circuit.

## Discussion

To find a therapeutic strategy for glomerular diseases [1], a better understanding of its underlying mechanisms is required. Among all etiology factors, podocyte injury and the inflammatory response play critical roles in all kinds of glomerular diseases [46–49].

Podocytes are terminally differentiated epithelial cells that constitute the filtration barrier. Podocytes are divided into cell body, primary process, and foot process, and express slit diaphragm proteins including Nephrin, NEPH1, and FAT to mechanically intercept albumin molecules, and also contribute to the negative charge of the filtration barrier [50]. Podocytes can also secrete matrix components for the formation of GBM and growth factors for the survival of endothelial cells [51]. Interestingly, recent studies have shown podocytes could also serve as non-hematopoietic APCs [5, 13], playing a role in immune responses and inflammation in glomerular diseases [52]. Hence, podocytes play a “gated key lock” role in glomerular diseases. Podocytes could present antigens to activate T lymphocytes and a series of immune responses through MHC II molecules and co-stimulatory protein B7-1 [13, 31]. The immune response contributes to the deterioration in various glomerular diseases such as LN, IgAN and MN [53–55]. Of note, the immunosuppressive therapies have achieved some efficacies, however, many patients are facing frequent relapse, temporary remission with reappearance of symptoms, and resistance to the immunosuppressive drugs [30, 56]. All of these evidences suggest that when podocytes acquire the characteristics of APC cells, this feature maybe trigger self-damage except activating immune response.

B7-1 is a co-stimulatory modulator, which primarily expresses in NK cells and macrophages. B7-1 binds with CD28 in T cells to induce their differentiation and activation [17]. Interestingly, B7-1 is also expressed in podocytes and plays a role in T cell polarization in kidneys [5, 31]. Besides the immune response, B7-1 can also promote cell injury by disrupting integrin and NEPH1 in podocytes [31, 32]. However, the role of B7-1 in podocyte injury remains controversial in the past few years, because of its inconsistent staining results in human kidney biopsies [22–24]. Therefore, an innovative technology should be adopted in clinical samples and a clinical cohort study in glomerular diseases should be conducted. Notably, B7-1 protein could be hardly seen in long-term preserved tissues and maybe just appear in a small part of patients [57]. The other puzzle is the unreliable treatment effects of abatacept [58]. After withdrawal of it, relapse commonly occurs [30]. The reason maybe lies that abatacept could only block the binding of B7-1 with CD28, it could not inhibit B7-1 expression. While the intrinsic role of B7-1 in podocyte’s self-injury should not be neglected. Thus, it is necessary to construct a podocyte-specific B7-1 transgenic model to assess its role in detail. Except that, one more specific and sensitive technology for B7-1 detection in human kidney tissues is also required.

RNAscope technology is an advanced technic for detecting weak expression, compared with immunological assessment [39]. Using RNAscope, B7-1 was found nearly in all types of glomerular diseases. This is inconsistent with previous studies, as they did not find B7-1 antigen protein expression in DN or others [23, 24]. We thought the reason possibly lies that antigens decay quickly, while genes persist. Furthermore, we found B7-1 is primarily expressed in podocytes, although previous literatures showed B7-1 also exists in endothelial and tubular cells [16, 59, 60]. Several proofs proved our findings. First, we found B7-1 was highly colocalized with α-actinin-4, a podocyte marker. We also performed the co-staining of B7-1 with EMCN, an endothelial cell marker, and found that there was only a very small part of co-localization. Second, to compare B7-1 expression in glomerulus and tubules, we isolated them from ADR mice, and found glomerular B7-1 was highly upregulated in a time-dependent manner, while tubular B7-1 only showed slightly increase in the late stage. Third, to testify whether there is a relationship between urinary B7-1 with tubular injury, we tested urinary β2-MG, a marker for tubular cell injury-derived proteinuria. In our clinical cohort, we found urinary B7-1 had no correlation with it. While urinary B7-1 is highly positively correlated with ACR, an indicator for glomerular proteinuria. Therefore, all these proofs suggest that podocytes are the main source for B7-1 production. Of interest, our investigation focuses in newly diagnostic patients, and identify that urinary B7-1 significantly increased from CKD G1 stage. This implicates urinary B7-1 might serve as a diagnostic marker in glomerular diseases. While the exact prognostic significance of urinary B7-1, in the remission or relapse of the disease, requires a prospective study in a large cohort, which is beyond the scope of this study.

Using podocyte-specific B7-1 transgenic mice, isolated glomerular mini-organ culture from B7-1^flox/flox^ mice and rat, CKD mice models, and clinical samples, etc., we proved that B7-1 itself is sufficient to induce podocyte injury. This finding strongly clarifies the role of B7-1 in podocyte injury. We also discovered β-catenin meditates B7-1 pathway. This was proved by multiple methods such as RNA sequencing and pharmacological inhibition. More importantly, we identified Hsp90ab1 is a key mediator for the signal transmission of B7-1 to β-catenin. We have also discerned its active binding site with B7-1. As a chaperone protein, Hsp90ab1 functions as “an accommodation house” for B7-1 and LRP5, and crucially facilitates the signal transmissions. Finally, we identified β-catenin triggers the transcription and translation of B7-1, suggesting B7-1 is an upstream factor for β-catenin through Hsp90 mediation, but also a downstream of β-catenin. Hence, we provided a new theory for podocyte injury, i.e. B7-1 mediates a vicious cycle through Hsp90ab1-mediated communication with the β-catenin pathway in podocytes. To targeted inhibit any node of the signal path would block the whole process. From the sequencing, we also found B7-1 involves in T cell immune response and podocyte cell apoptosis, these results also deserve detailed analysis in the future.

In our study, we found Hsp90ab1/β-catenin signaling is a novel and major downstream effector of B7-1. The previous findings reported the role of integrin signaling in B7-1-induced podocyte injury [31]. We found that Hsp90ab1 is also involved in that. Both β-catenin and integrin signaling pathways may function in parallel or collectively. This could be assessed in the future. Nevertheless, we found that β-catenin plays a key role in B7-1-induced podocyte injury through Hsp90ab1. Our study further enriches the understanding of β-catenin pathway. Except of its classical induction through Wnt(s) [34], β-catenin could be activated by multiple factors such as Hsp90ab1. Consistently, we found that β-catenin could be activated by CB2/Src recently [61].

Our study identified Hsp90ab1 plays a key role in the signal transmission of B7-1 to β-catenin and podocyte injury. Thus, combining an Hsp90 inhibitor with abatacept in refractory nephrotic syndrome may achieve better therapeutic effects in patients. This deserves extensive further investigation. We also found β-catenin is both upstream and downstream effector of B7-1 signals. Hence, managing patients with β-catenin blocker should be also advantageous. Although more studies are needed, our study provides an important indication to help to resolve the ambiguous issues of B7-1 in podocyte injury and glomerular diseases.

## Methods

The detailed methods are presented in Supplementary Material.

### Human urine samples and kidney biopsies

Human urine samples and the fresh frozen kidney sections were collected from patients with newly diagnosis of primary glomerular disease. The control tissues were derived from paracancerous tissues of patients who were performed radical nephrectomy. The demographic and clinical data are presented in Supplementary Table S1.

### RNAscope

The in situ hybridization was performed using the RNAscope® Multiplex Fluorescent Reagent Kit v2 (Advanced Cell Diagnostic, Inc), and the detection probe against human B7-1 (421471, Advanced Cell Diagnositc Inc.), and negative control probe DapB (421471, Advanced Cell Diagnositc Inc.). According to the manufacturer’s, the fresh frozen kidney sections (6 μm) were fixed, dehydrated and pre-treated with hydrogen peroxide. For ISH staining, sections were then digested with protease III for 18 mins at room temperature, hybridized with target probes, amplified, and labeled with fluorophore Opal 570 at 40°C in the HybEZ^TM^ Oven. For co-staining with antibodies, we applied the RNA-protein Co-Detection Ancillary kit (323180, Advanced Cell Diagnostic, Inc) and pre-incubated with the primary antibodies before digestion. Detailed methods were shown in Supplementary Methods.

### Animal models

All mice were purchased from the Experimental Animal Center of Southern Medical University (Guangzhou, China) or Cyagen (Cyagen Biosciences Inc, China). All animal studies were approved by the Animal Experimentation Ethic Committee at the Nanfang Hospital, and were performed in compliance with the Guidelines for the Care and Use of Laboratory Animal.

### Generation of podocyte-specific B7-1 transgenic mice

The podocyte-specific B7-1 transgenic mice (Tg mice) were generated in C57BL/6 background by using PiggyBac transposon system. Genotyping was confirmed by PCR analysis in tail samples from mice at three weeks of age. The F0, F1 and F2 generations were all produced in Cyagen (Cyagen Biosciences Inc, China). In this study, the F1 generations were adopted. Tg and their control mice (WT) were sacrificed at indicated time. Tg mice at 6-month old were treated with adeno-associated virus 9 (AAV9) for interference of Hsp90ab1. Briefly, AAV9 carrying Hsp90ab1 interference sequences (AAV9-shHsp90ab1, 1 × 10^12^ copies/ml) or the negative control were established by HanBio company (Shanghai, China). Either AAV9-shHsp90ab1 or AAV9-NC was administered at 60 μl of volumes per mouse by in situ injections (six locations) to the cortex region. Mice were scarified and kidney tissues were harvested three months after AAV treatment.

### Generation of B7-1^flox/flox^ mice

The B7-1^flox/flox^ mice were generated in C57BL/6 background by CRISPR/Cas9 system and produced in Cyagen (Cyagen Biosciences Inc, China). Genotyping was then confirmed by PCR analysis in tail samples from mice at three weeks of age.

### Adriamycin (ADR)-induced nephropathy in mice

Male Balb/c mice (eight weeks of age, weighed 20–25 g) were administered ADR (11.5 mg/kg) by intravenous injection through tail vein. Urine samples were collected weekly to assess for albuminuria. Mice were euthanized at indicated time. Saline injection was applied to control mice. For gene interference of B7-1 or Hsp90ab1, hydrodynamic-based gene delivery approach was applied. Some mice were treated with 17-AAG at 5 or 10 mg/kg every other day. The detailed experimental designs are presented in Figures.

### 5/6 nephrectomy (5/6NX) model

For 5/6NX models, male CD-1 mice, weighing 23–25 g, were subjected to two surgical resections of two thirds of the left kidney and the whole right kidney, or sham operation, as previously described [42]. Two weeks after the first operation (week 2), the 5/6NX mice were randomly divided into indicated groups.

### Isolation of glomeruli and tubules

Glomeruli from mice were isolated as described [33]. Briefly, mice were sacrificed and perfused with Dynabead M-450 (00388551, Invitrogen). The kidneys were digested in collagenase IV (1 mg/ml, Invitrogen) and pressed through a 100-μm cell strainer (BD Falcon, Bedford, MA), and then glomeruli were gathered using a magnetic concentrator, and the remaining fluids were centrifuged for collecting the tubules.

For isolation of rat glomeruli [36], SD rats were sacrificed in a sterile environment. Kidneys were harvested, decapsulated, minced, and then smashed down with a plunger through three sieves sequentially (200-100-60 mesh opening sizes). After washing and centrifugation, the glomeruli were resuspended in RPMI1640 with 10% FBS medium and plated on noncoated six-well plates for treatment with lentivirus expressing B7-1 (Genechem, Shanghai, China).

### Cell culture and treatment

The conditionally immortalized mouse podocyte cell line (MPC5) was cultured and maintained as described previously [33]. MPC5 cells were synchronized into quiescence by growing in serum-free medium, and then treated with 0.25 μg/ml of ADR or 100 ng/ml of recombinant Wnt3a protein for 24 h. Some cells were pre-treated with ICG-001 (5 μmol/L) or 17-AAG (1 μmol/L) for 1 h before indicated treatment. The plasmid or siRNA transfection was carried out using Lipofectamine 2000 reagent (11668-019, Invitrogen). 293 T cells, human umbilical vein endothelial cell (HUVEC) and rat mesangial cell (RMC) were also cultured by routine procedures.

### Histology and immunohistochemical staining

Paraffin-embedded mouse kidney sections (3 μm) were prepared and performed immunohistochemical staining using routine protocols. The primary antibodies were indicated in Supplementary Table S2. Some sections were stained with periodic acid-Schiff (PAS) (BA4080A, BASO). Images were photographed by Olympus BX53 microscope with EMCCD camera. At least ten glomeruli per mice in one section were analyzed to quantify positive area through the Image Pro plus software V6.0 (Media Cybernetics, Inc., Rockville, USA).

### Immunofluorescence staining

The kidney cryo-sections and cover slips of cultured cells were fixed with 4% PFA for 15 min at room temperature, following incubating primary antibodies (Supplementary Table S2) overnight at 4 °C. The mouse isotype control IgG was also used to check the specificity of antibody. The glomeruli sediments were embedded in OCT and sectioned at 5 μm thickness, and then fixed in 4% PFA for 30 min at room temperature, following incubation with antibodies.

For immunofluorescence staining of B7-1, the kidney frozen sections were prepared from kidney tissues stored at −80 °C within one month. For F-actin staining, MPC5 cells were fixed with 4% PFA and performed staining according to manufacturer’s instructions (40734ES75; Yeasen, Shanghai, China). All images were taken by confocal microscopy (Leica TCS SP2 AOBS; Leica Microsystems, Buffalo Grove, IL) or Olympus DP80 microscope with EMCCD camera (Olympus, Tokyo, Japan).

### Western blot analysis and coimmunoprecipitation

Western blot analysis was performed by routine procedures. In brief, tissues and cell pellets were lysed in lysis buffer containing protease inhibitors, and thoroughly homogenized to lysate by Lu Ka Sample Grinder (LUKYM24). Proteins were separated by 8% or 10% SDS-PAGE and transferred onto PVDF membranes. The membranes were incubated with antibodies as indicated (Supplementary Table S2) overnight at 4 °C, and visualized with ECL. The coimmunoprecipitation procedure was as following: Protein lysates were immunoprecipitated overnight at 4 °C with indicated antibodies and protein A/G plus agarose (sc-2003; Santa Cruz Biotechnology). The precipitated complexes were washed for five times and boiled in SDS sample buffer followed by immunoblotting. All of the coimmunoprecipitation experiments were repeated at least three times. Original western blots for all relevant figures are shown in “Supplementary File 1—Full unedited gels”.

### Protein-binding sites prediction

The molecular structures of B7-1, Hsp90ab1 and integrin β1 were obtained from the RCSB PDB database, and the structure of LRP5 was established based on the homology modeling technique in Discovery Studio 2019. Sequence analysis showed a high conservation between human and mouse sequences of B7-1 and Hsp90ab1 protein. Molecular docking was performed using ZDOCK and RDOCK program (in Discovery studio 2019), and the optimal binding conformation was analyzed.

### Bioinformatic analyses

The gene ontology (GO) function annotation of genes from RNA-seq were based on the GO database (http://geneontology.org), and the functional enrichment analyses of DEGs were performed using Clusterprofiler in R through Fisher’s Exact Test (*P* < 0.05). Heatmaps of relative gene expression from RNA-seq were generated based on FPKM values using TBtool software [62]. For GSEA analysis, the involved gene sets were all derived from the Molecular Signatures Database of GSEA web interface. The GO-based pathway analysis and enrichment of the differential proteins was performed on Metascape (http://metascape.org) as described [63]. The protein-protein interaction network was established using STRING database (http://string-db.org).

### Statistical analyses

Statistical analyses were performed using SPSS 20.0 (SPSS Inc. Chicago, IL). All data were presented as means ± SEM. Two group comparisons were made using unpaired Student’s *t* test. Multiple group comparisons were assessed using one-way ANOVA. Correlation between urinary B7-1 and albumin/urine creatinine ratio (ACR), estimated glomerular filtration rate (eGFR), β-2-microglobulin (β2-MG) was determined using Spearman (nonparametric) analysis. *P* < 0.05 was considered statistically significant.

## Supplementary information

Checklist

Author contribution form

Supplementary detailed method&Supplementary Figure legends&Supplementary Tables

Supplementary Figure S1

Supplementary Figure S2

Supplementary Figure S3

Supplementary Figure S4

Supplementary Figure S5

Supplementary Figure S6

Supplementary Figure S7

Supplementary Figure S8

Supplementary Figure S9

Full unedited gels

## Data Availability

Transcriptomic data produced in this study are available at NCBI with accession number PRJNA765221 and PRJNA765122. The mass spectrometry proteomics data are available via ProteomeXchange with identifier PXD28856. All data needed to evaluate the conclusions in the paper are present in the paper and/or the Supplementary Materials.
