# Supplementary material for: B7-1 mediates podocyte injury and glomerulosclerosis through communication with Hsp90ab1-LRP5-β-catenin pathway
Source: Cell Death Differ. 2022 Jun 16;29(12):2399–416. doi: 10.1038/s41418-022-01026-8 (PMC9750974; doi:10.1038/s41418-022-01026-8)
Supplement: Supplementary file 2 — Author contribution form [file 41418_2022_1026_MOESM2_ESM.pdf]

DECLARATION OF CONTRIBUTIONS TO ARTICLE

ADMC

Manuscript Number: CDD-21-2784

Journal Name: *Cell Death & Differentiation* (the 'Journal')

Proposed Title of the Contribution:

B7-1 mediates podocyte injury and glomerulosclerosis through communication with Hsp90ab1-LRP5-β-catenin pathway (the 'Contribution')

Author(s):

Jiemei Li, Jing Niu, Wenjian Min, Jun Ai, Xu Lin, Jinhua Miao, Ye Liang, Shuangqin Chen, Qian Ren, Shan Zhou, Kunyu Shen, Qinyu Wu, Xiaolong Li, Weiwei Shen, Fan Fan Hou, Youhua Liu, Peng Yang , Lili Zhou. (the 'Authors')

For all *CDD* articles, each person named as an author in the published version must be able to show he or she has contributed substantially to the article.

Authorship credit should be based on 1) substantial contributions to conception and design, acquisition of data, or analysis and interpretation of data; 2) drafting the article or revising it critically for important intellectual content; and 3) final approval of the version to be published. Authors should meet conditions 1, 2 and 3.

Any person who cannot be shown to have made a substantial contribution to the article cannot be listed as an author in the final version. The name of any person who is deemed to have made a minor contribution can, however, appear in the Acknowledgments section of the article.

Please complete the table below to indicate the contributions of all named authors to the manuscript.

Author Full Name:	Specification of Contribution to the Manuscript:
Lili Zhou	Conception and design, wrote and reviewed the manuscript.
Jiemei Li, Jing Niu	Acquisition of data and analyzed data, creation of figures and wrote the manuscript
Jinhua, miao, Shan Zhou	Creation of animal models and cell culutre
Wenjian Min, Jiemei Li	Application of software, bioinformatics analysis and perform the protein binding relative experiments
Jun Ai, Xu Lin	Acquisition of human sample data and analyzed data, reviewed the manuscript
Ye Liang, Shuangqin Chen	Preparation of reagent and provied help for in vitro study
Qian Ren, Weiwei Shen	Preparation of reagent and provied help for animal experiments
Kunyu Shen,Qinyu Wu, Xiaolong Li	Collected data
Peng Yang,Fan Fan Hou, Youhua Liu	Reviewed and advised about the manuscript.
All authors	All authors discussed the resluts and commented on the manuscript.

Please complete the table below to indicate the contributions of all named authors to the figures.

Figure 1:

JA, LX, JN and JL perform the eperiments. JA and LX collected the human urine and kidney biopsies sample, and analyzed the data and assembled the figure.

Figure 2:

JN, JL and JM collected the data, JN analyzed the data and assembled the figure.

Figure 3:

JN, JM,QR and WS collected the data, JN analyzed the data and assembled the figure.

Figure 4:

SZ,JN,JL,YL collected the data. SZ analyzed the data and assembled the figure.

Figure 5:

WM, JL and SC collected the data, WM and JL analyzed the data and assembled the figure.

Figure 6:

WM, JL, SC and KS collected the data, WM and JL analyzed the data and assembled the figure.

Figure 7:

JL,JM,XL,QW collected the data, JL and JM analyzed the data and assembled the figure.

Figure 8:

JM, JL and SZ collected and analyzed the data, assembled the figure.

Figure 9:

JN,JL,SC,YL collected the data, JL analyzed the data and assembled the figure.

Signed for and on behalf of the Author(s):	Print Name:	Date:
Lili Zhou	Lili Zhou	May 21,2022
