# Supplementary material for: B7-1 mediates podocyte injury and glomerulosclerosis through communication with Hsp90ab1-LRP5-β-catenin pathway
Source: Cell Death Differ. 2022 Jun 16;29(12):2399–416. doi: 10.1038/s41418-022-01026-8 (PMC9750974; doi:10.1038/s41418-022-01026-8)
Supplement: Supplementary file 3 — Supplementary detailed method&Supplementary Figure legends&Supplementary Tables [file 41418_2022_1026_MOESM3_ESM.docx]

Supplementary Materials for

**B7-1 mediates podocyte injury and glomerulosclerosis through communication with Hsp90ab1-LRP5-β-catenin pathway**

**Supplementary Materials include:**

Supplementary detailed methods

Supplementary Figure legends

Supplementary Tables S1, S2

Supplementary Figures S1 to S9

Supplementary File 1 Full unedited gels

**Supplementary detailed methods**

**Experimental Design**

The purpose of this study was to address the controversial role of B7-1 in podocyte injury and discern the mechanism using various glomerular injury models and human biopsies. B7-1 RNA expression in human with a variety of glomerular diseases was assessed through RNA scope, and correlative analysis of urinary soluble B7-1 with disease severity was performed in new-onset patients without immunosuppressive therapy. For animal experiments, podocyte-specific B7-1 transgenic mice and B7-1^flox/flox^ mice were established. RNA-seq and biological assays were performed in 6-month-old wildtype (WT) and Tg mice. The effects of B7-1 knockdown or Hsp90ab1 were tested in ADR-treated mice and cultured podocytes. Multiple methods of RNAseq, LC-MS/MS, molecular modeling, amino acid mutation, ChIP-PCR, etc., were used to determine the role of HSP90ab1/β-catenin pathway in B7-1-induced podocyte injury. All *in vitro* experiments were repeated at least three times. Statistical analyses were performed as described. Detailed information of the involved reagents would be found in Supplementary Table S2.

**Human urine samples and kidney biopsies**

All human studies were performed with informed patient consent and were approved by the Institutional Ethics Committee at Nanfang Hospital (NFEC-2019-209). Human urine samples and fresh frozen kidney sections were collected from patients with newly diagnosis of primary glomerular disease. The control tissues were derived from para-cancerous kidney tissues of patients who had radical nephrectomy. The demographic and clinical data of patients involved are presented Supplemental Table S1.

**Urine B7-1 ELISA**

The urinary concentration of B7-1 was determined using a human sCD80 Instant ELISA kit (eBioscience, BMS291INST) according to the manufacturer’s protocol, respectively. The concentration of B7-1 was calculated according to standard curve and corrected by urinary creatinine.

**RNA scope**

The in situ hybridization was performed using the RNAscope® Multiplex Fluorescent Reagent Kit v2 (Advanced Cell Diagnostic, Inc) and detection probe against human B7-1 (421471, Advanced Cell Diagnositc,Inc.) and negative control probe DapB (421471, Advanced Cell Diagnositc,Inc.). According to the manufacturer’s standards, samples were obtained from kidney biopsies and stored at -80℃ within three months. The fresh frozen sections (6μm) were mounted on SuperFrost Plus slides and kept at -20℃ to dry for one hour. Subsequently, the slides were fixed in 4% paraformaldehyde (PFA) on ice for 15 minutes and immediately used for alcoholic dehydration procedure. The slides were kept drying at room temperature for 5 minutes and hydrogen peroxide was applied to each section for 10 minutes before proceeding to ISH staining or co-detection with immunofluorescence staining. For ISH staining, tissue sections were then digested with protease III for 18 minutes at room temperature, hybridized with target probes, amplified, and labeled with fluorophore Opal 570(Perkin Elmer) at 40℃ in the HybEZ^TM^ Oven.

For co-detection with α-actinin-4 or β-catenin, the primary antibodies or negative control isotype mouse IgG were applied to the sections and incubated overnight at 4℃ following performed as the Integrated Co-Detection Workflow (ICW) kit protocols with RNA-protein Co-Detection Ancillary kit (323180, Advanced Cell Diagnostic, Inc). Following primary antibody incubation, the slides were post-fixed in 10% Neutral Buffered Formalin (NBF) for 30 minutes at room temperature before ISH staining procedure as described above. After hybridized with amplifying signal reagents, tissue sections were washed in washing buffer, incubated with secondary antibodies for 30 minutes at room temperature. Slides were mounted with DAPI and sealed.

**Animal models**

Male C57BL/6J mice, Balb/c mice, CD1 mice, db/db mice (20-week-old), and db/m mice (20-week-old) were purchased from the Experimental Animal Center of Southern Medical University (Guangzhou, China). All animal studies were approved by the Animal Experimentation Ethic Committee at the Nanfang Hospital, and were performed in compliance with the Guidelines for the Care and Use of Laboratory Animal.

**Generation of podocyte-specific B7-1 transgenic mice**

The podocyte-specific B7-1 transgenic mice (Tg mice) were generated in C57BL/6 background by using piggybac transposon system. The gene of B7-1 was recombined with the NPHS2 promoter and 3×Flag tag. Genotyping was confirmed by PCR analysis using tail samples from mice at 3 weeks of age. The F0, F1 and F2 generations were all produced in Cyagen (Cyagen Biosciences Inc, China). In this study, the F1 generations were sacrificed at 2 months, 3 months or 6 months of age without intervention. Tg and their wild-type littermates (WT) were sacrificed at indicated time.

**AAV treatments of Hsp90ab1 RNA interference**

The 6-month-old age Tg and WT mice were treated with adeno-associated virus for interference of Hsp90ab1 gene. Adeno-associated virus 9 vectors (AAV9) carrying Hsp90ab1 interference sequences (AAV9-shHsp90ab1, 1 x 10^12^ copies/ml) or negative control (AAV9-NC) were established by HanBio company. According to the manufacturer’s instruction, either AAV9-shHsp90ab1 or AAV9-NC was administered at 60 *μl* volumes of vector per mouse. To deliver the vector, we performed multi-point injection around the renal cortex region by using 33G, 50μl Hamilton syringes (7803-05, 7655-01, Hamilton Company). Briefly, the Tg mice and wildtype (WT) mice were anesthetized and laid in prone position. An incision (approximately 1 cm) was made in the right posterior so that the right kidney was exposed. Injections were performed by carefully piercing into the kidney cortex, then slowly injecting with 10 *μl* of vector at one site. After injection and needle withdrawal, a dry cotton swab was used for 1 min for hemostasis and fluid leakage oppression. The six selected sites were shown in Figure 6. Urine samples were collected monthly to assess albuminuria. Mice were scarified and samples were harvested for analysis after 3 months after AAV treatment.

**Generation of B7-1^flox/flox^ mice**

The B7-1^flox/flox^ mice were generated in C57BL/6 background by CRISPR/Cas9 system and produced in Cyagen (Cyagen Biosciences Inc, China). Genotyping was then confirmed by PCR analysis in tail samples from mice at 3 weeks of age.

**Adriamycin (ADR)-induced nephropathy in mice**

Male mice (8 weeks of age) weighing 20-25g, were administered ADR (11.5 mg/kg) by intravenous injection through tail vein. Saline injection was applied to the control mice. Urine samples were collected weekly to assess albuminuria. Mice were euthanized and the kidney tissue samples were harvested for analysis at indicated time.

For gene interference of B7-1 or Hsp90ab1 in ADR models, shRNA vector encoding the interference sequence for B7-1 (pLVX-shB7-1), Hsp90ab1(Plvx-shB7-1) or negative control (Ctl-shR) was injected by hydrodynamic-based gene delivery approach. Briefly, 33 μg of shRNA vectors were diluted in 1.8 ml of saline and injected via the tail vein within 10s for each mouse. Plasmids were administered two days before and seven days after ADR injection. The control group of mice also received Ctl-shR plasmid injection. Mice were sacrificed two weeks after ADR injection.

For 17-AAG administration, drugs were dissolved to 1mg/ml by 5% Dimethyl sulfoxide (DMSO) and 95% corn oil and injected as 5mg/kg or 10mg/kg via intraperitoneal injection every other day. The control mice receive equal volume of solvent medium. Urine and kidney tissue samples were harvested for analysis one week after ADR injection.

**5/6 nephrectomy (5/6NX) model**

For 5/6NX models, male CD-1 mice, weighing 23-25 g, were subjected to two surgical resections of two thirds of the left kidney at the first week and the whole right kidney at the second week, or sham operation. Two weeks after the first operation (week 2), the 5/6NX mice were randomly divided into two groups and administrated with B7-shRNA or Ctl-shRNA every week for interference of B7-1 as described above, the sham group also received Ctl-shRNA. At week 6, all mice were sacrificed, and urine and kidney tissue were collected for various analyses.

**Isolation of glomeruli**

The isolation of glomeruli from mice was performed by magnetic beads. Briefly, mice were sacrificed and perfused with 10 ml of PBS, followed by 20 ml of diluted Dynabead M-450 (00388551, Invitrogen) perfusion. The kidneys were cut into 1 mm^3^ pieces and digested in collagenase IV (1 mg/ml, Invitrogen) at 37 °C for 15 min. The tissue was pressed through a 100-μm cell strainer (BD Falcon, Bedford, MA), and then glomeruli were gathered using a magnetic concentrator, and the remaining fluids were centrifuged at 600 ×g for 5min at 4 ℃ for collecting the tubules. After washing and observing under a microscope to ensure the absence of tubular fragments, the isolated glomeruli were then lysed in buffers or embedded for the subsequent experiments.

For the isolation of rat glomeruli, 8-week-old male SD rats weighing 250 g, were anesthetized and operated in a sterile environment. Both kidneys were harvested, decapsulated and minced in sterile PBS. Kidney tissues were then smashed down with a plunger through 3 sieves sequentially (200-100-60 mesh opening sizes). After washing and centrifugation, the glomeruli were resuspended in RPMI1640 with 10% FBS medium and plated on noncoated six-well plates for later transfection of lentivirus.

**Lentivirus transfection**

The primary isolated rat glomeruli were transfected with B7-1 overexpression lentivirus. The construction of B7-1 lentivirus (Flag Tag) and empty vector were done in Gene Chemical Technology Company (Genechem, Shanghai, China). According to the manufacturer’s instruction, the isolated glomeruli were plated approximately 20-30% confluent in a 6-well plate with a 2 ml of mixture of polybrene (REVG0001, 5 μg/ml) and lentiviral particles (2 μl) for 72 hours. Glomeruli were harvested for immunofluorescence, protein and mRNA analyses. In the second set of experiments, the isolated rat glomeruli were pre-treated with 5 μmol/L of ICG-001 or DMSO for 1 hour before lentiviral transduction.

**Adenovirus treatment**

To specifically knockdown the expression of podocyte B7-1, adenovirus vector carrying the Cre recombinase gene driven by NPHS2 promoter (AdV-NPHS2-Cre) and control adenovirus (AdV-NC) were made by Cyagen Biosciences. The isolated glomeruli obtained from B7-1 ^flox/flox^ mice by magnetic beads were plated approximately 50-60% confluent in a 6-well plate with a 2 ml mixture of complete medium and 10 μl adenovirus (MOI, 100). After adenovirus infection for 48 h, 0.125 μg/ml of ADR was added into the isolated glomeruli for another 24 h. Glomeruli were harvested for protein and mRNA analyses.

**Cell culture and treatment**

The conditionally immortalized mouse podocyte cell line MPC5 was cultured and maintained. In brief, MPC5 cells were cultured at 33℃ in RPMI1640 medium supplemented with 10% FBS and 10 units/ml of recombinant IFN-γ (IF005, Sigma) to propagate podocytes; and then podocytes were grown at 37℃ in the absence of IFN-γ for 7 days to induce cell differentiation before experiments. Podocytes were synchronized into quiescence by growing cells in serum-free medium before treatments with 0.25μg/ml of ADR or 100 ng/ml of recombinant Wnt3a protein for 24 hours. For some experiments, ADR were added 24 h after indicated treatments. In some experiments, ICG-001 (5 μmol/L) or 17-AAG (1 μmol/L) were pre-treated to cells for 1 hour before indicated treatments.

Other cells lines were also used in our studies: 293T cells were cultured using high-glucose DMEM medium and incubated at 37℃; Endothelial cells (Human umbilical vein endothelial cell, HUVEC) were cultured in high-glucose DMEM at 37℃; Rat mesangial cells (RMCs) were cultured in low-glucose DMEM medium at 37℃.

**SiRNA-mediated knockdown and DNA transfections**

For transient transfection, 50%-80% of confluent cells were transfected with siRNA for target genes or negative control. Plasmids of target genes or empty vector (pcDNA3.1, Invitrogen) were transfected using the lipofectamine 2000 (Life Technologies) according to the manufacture’s protocols. siRNA sequences are listed in Supplementary Table S2.

**Urinary albumin, creatine, and β2-MG assay**

Urinary albumin was measured using a mouse albumin ELISA kit, following the manufacturer’s instruction ((Bethyl Laboratories, Inc., Montgomery, TX). Urinary creatinine was determined using QuantichromTM Creatine Assay kit (DICT-500, BioAssay Systems, Hayward, USA). Urinary albumin was standardized to creatinine and expressed as mg/mg Ucr. Urinary β2-MG was assessed using a commercial kit (OQWU15, Siemens Healthcare Diagnostic Products, GmbH, Germany)

**Transmission electron microscopy (TEM)**

Kidney cortex was harvested and fixed in 1.25% glutaraldehyde/0.1 M phosphate buffer, and then embedded by resin and ultrafine section making. Slides were subjected to assess podocyte ultrastructure under an electron microscope (JEOL JEM-1010).

**Histology and immunohistochemical staining**

Paraffin-embedded mouse kidney sections (3 μm) were prepared by a routine procedure. Immunohistochemical staining were performed using a routine protocol. The primary antibodies were indicated in Supplementary Table S2. The isotype-matched IgG was used to check for antibody specificity. Sections were also stained with periodic acid-Schiff (PAS) staining (BA4080A, BASO) to identify glomerular injury. Images were photographed by Olympus BX53 microscope with EMCCD camera (Olympus, Tokyo, Japan). At least ten glomeruli per mice within one section were analyzed and quantified the positive area/glomerulus area using the Image Pro Plus software V6.0 (Media Cybernetics, Inc., Rockville, USA).

**Immunofluorescence staining**

To prepare fresh frozen tissue sections, the fresh mouse specimens were embedded with cryo-embedding (OCT) and cross-sectioned (4 *μm*) for immunofluorescence staining. Kidney cryo-sections and mouse podocyte cultured on cover slips were fixed with 4% PFA for 15 minutes at room temperature. Paraffin-embedded sections (1 *μm*) were dewaxed, hydrated and conducted antigen repair by microwave thermal incubation with primary antibodies. Different primary antibodies were incubated (Supplementary Table S2) overnight at 4 ℃ and washed with 1x PBS, and then stained with secondary antibodies (Jackson ImmunoResearch Laboratories). The mouse IgG antibody was used to check for antibody specificity.

For immunofluorescence staining in isolated glomeruli, the extracted or cultured glomeruli were collected in Eppendorf tubes and centrifugated at 4℃ for 10 min at 1,000 rpm. The glomeruli sediments were then frozen on dry ice within 5 minutes of tissue harvest, embedded with OCT in the cryomold and frozen into blocks at -80 ℃. The frozen blocks were then sectioned into slides with 5 *μm* thickness and fixed in 4% PFA for 30 minutes at room temperature, following the routine procedure of incubation with antibodies.

For B7-1 immunofluorescence staining, the fresh frozen sections were prepared from cryo-embedding tissues stored at -80℃ within one month. After fixation with 4% PFA, sections were pretreated with 70% ethanol for 20 minutes on ice and then incubated with 3% BSA for 3 hours before incubating with primary antibodies.

For F-actin staining, MPC5 cells were fixed with 4% paraformaldehyde andd performed according to manufacturer’s instruction by using their staining kits (40734ES75; Yeasen, Shanghai, China).

All images were taken by confocal microscopy (Leica TCS SP2 AOBS; Leica Microsystems, Buffalo Grove, IL) or Olympus DP80 microscope with EMCCD camera (Olympus, Tokyo, Japan).

**Western blot analysis and coimmunoprecipitation**

Western blot analysis was performed by a routine procedure. In brief, tissues and cell pellets from culture were resuspended in lysis buffer containing protease inhibitors, and thoroughly homogenized by Lu Ka Sample Grinder (LUKYM24). Tissue lysates and cells lysates were then centrifugated at 4℃ for 10 min at 12,000 rpm. Following Centrifugation, soluble supernatant was carefully transferred to an Eppendorf tube and protein concentration was quantified by BCA assay. Proteins were separated by 8% or 10% SDS-PAGE and transferred onto PVDF membranes. Then membranes were incubated with antibodies as indicated (Supplementary Table S2), and samples were visualized with ECL. Original western blots for all relevant figures are shown in “Supplementary File 1—Full unedited gels”.

The coimmunoprecipitation procedure was as following: Protein lysates were immunoprecipitated overnight at 4 °C with antibodies or IgG and protein A/G plus agarose (sc-2003; Santa Cruz Biotechnology). The precipitated complexes were washed with lysis buffer for 5 times and boiled for 10 min in SDS sample buffer followed by immunoblotting. All of the coimmunoprecipitation experiments were repeated at least 3 times. Primary antibodies used are described in the supplementary Table S2. Original western blots for all relevant figures are shown in “Supplementary File 1—Full unedited gels”.

**Chromatin immunoprecipitation (ChIP)**

ChIP was performed using the SimpleChIP® Plus (Magnetic Bead) Kit (9005, Cell Signaling Technology), according to the manufacturer’s instruction. Briefly, cross-linked chromatin was fragmented by partial digestion with micrococcal nuclease to obtain chromatin fragments of 1 to 5 nucleosomes. The chromatin was immunoprecipitated using various antibodies (described in supplementary Table S2) and magnetic beads. H3 antibody and normal rabbit IgG were served as positive and negative control. PCR was performed with GoTaq Green Master Mix (M7123, Promega) using the primers listed in Supplementary Table S2.

**Transcriptomic analysis**

Total RNA from kidney tissues and cultured podocytes were extracted using a TRIzol RNA isolation system (Life Technologies, Grand Island, NY). The procedure of establishing cDNA libraries and sequencing were completed at Illumina Hiseq platform by Shanghai Zhongke Biotech Company. The clean reads were mapped to the mouse genome using HISAT2 software, and normalized by converting the fragment counts to FPKM value. Differentially expressed genes (DEGs) were then calculated as the ratio of FPKM values and identified by the DESeq2 package (|log2foldchange|>1, padj<0.05).

**Co-IP-MS/MS**

Co-IP and LC-MS/MS analysis were performed by Guangzhou Fitgene Biotechnology Co. Briefly, Co-IP was performed using Flag M2 beads (M8823, Sigma) and the resultant pellet was lysed and digested into fragments of peptides. The peptides were suspended in 2% acetonitrile and 0.1% formic acid for Liquid Chromatogram (LC) run. Samples were loaded onto a 75 μm×150 mm Acclaim PepMap C18 (160321, Thermo) reversed-phased column packed with Acclaim PepMap RSLC C18 (160454, Thermo). Separated peptides were directly analyzed with a Mass spectrometer (Thermo Scientific Q Exactive) for detection. The resulting spectra were recorded and searched on MASCOT using the reviewed Uniport database.

**Bioinformatic analyses**

The GO function annotation of genes from RNA-seq were based on the GO database (http://geneontology.org), and the functional enrichment analyses of DEGs were performed using Clusterprofiler in R through Fisher’s Exact Test (P<0.05). Heatmaps of relative gene expression from RNA-seq were generated based on FPKM values using TBtool software. For the GSEA analysis, the involved gene sets were all derived from the Molecular Signatures Database of GSEA web interface. The gene ontology-based pathway analysis and enrichment of the differential proteins was performed on Metascape (http://metascape.org) as described elsewhere, and the chord graphic was drawn by http://www.bioinformatics.com.cn, an online platform for data analysis and visualization. The protein-protein interaction network was established using STRING database (http://string-db.org).

**Protein-binding sites prediction**

The molecular structures of B7-1, Hsp90ab1 and integrin β1 were obtained from the RCSB PDB database, and the structure of LRP5 was established based on the homology modeling technique in Discovery Studio 2019. Sequence analyses showed a high conservation between human and mouse sequences for B7-1 and Hsp90ab1 protein. Molecular docking was performed using ZDOCK and RDOCK program (in Discovery studio 2019), and the optimal binding conformation was analyzed.

**Statistical analyses**

Statistical analyses were performed using SPSS 20.0 (SPSS Inc. Chicago, IL). All data were presented as *means* *± SEM*. Two group comparisons were made using unpaired Student’s t test. Multiple group comparisons were assessed using one-way ANOVA. Correlation between urinary B7-1 and albumin/urine creatinine ratio (ACR), estimated glomerular filtration rate (eGFR), β-2-microglobulin (β2-MG) was determined using Spearman (nonparametric) analysis. *P*<0.05 was considered statistically significant.

**Supplementary Figure legends**

Fig. S1. Podocyte is the major source of B7-1 in glomerular diseases. (A) Representative micrographs of B7-1 RNA scope staining in human kidney cortical tissue from patients with minimal change disease (MCD), IgA nephropathy class IV (IgAN), primary focal segmental glomerulosclerosis (FSGS), nodular diabetic nephropathy (DN) and Membranous Nephropathy (MN). The tracing outline indicates the minimal polygon around the glomerular tuft area. Bar=50 *μm*. (B) Images showing co-staining of negative control probe and mouse IgG in Fresh frozen kidney section from LN patient. Bar=50 *μm*. (C) Images showing colocalization between B7-1 RNA scope staining and α-actinin-4 in fresh frozen kidney section from FSGS patient. Arrows indicate specific co-localization in podocyte. Bar=50 *μm*. (D) Images showing co-staning of β-catenin and α-actinin-4 in fresh frozen kidney section from healthy control. Bar=50 *μm*. (E) Representative images showing co-expression of β-catenin and α-actinin-4 in paraffin-embedded sections from healthy control and FSGS patient. Arrows indicate specific co-localization in podocyte. Bar=50 *μm*. (F) Representative images showing isotype matched mouse IgG staining in fresh frozen kidney section and paraffin-embedded kidney section to check for B7-1 and β-catenin antibodies specificity. Bar=25 or 20 *μm.* (G) Images showing co-staining of B7-1 and EMCN in frozen kidney sections from control and ADR-induced nephropathy mice. Bar=10 *μm.* (H) Images showing co-staining of B7-1 and β-catenin or B7-1 and Podocalyxin in isolated control glomeruli. Bar=25 *μm* or 10 *μm.*

Fig. S2. B7-1 is associated with podocyte injury and T cell immune response. (A) Representative images showing B7-1 and Flag-tag co-staining in 2-, 3-, or 6-month-old WT mice and Tg mice. Arrows indicate specific co-localization. Bar=25 *μm.* (B-D) Western blot and quantification of Podocalyxin, Synaptopodin, Nephrin, Active β-catenin, β-catenin and B7-1 in WT mice at different age are shown. *n*=5. (E) Representative images showing podocyte ultrastructure by TEM micrograph (Bar=1 *μm*), immunofluorescence staining of Nephrin (Bar=10 *μm*), or glomerular structure assessed by PAS staining (Bar=10 *μm*) in Tg and WT mice at indicated age. (F-H) Graphs showing the comparison of blood urea nitrogen (BUN), serum creatinine (SCR), or weight between Tg mice and WT mice at different age. ^*^*P*<0.05 versus WT mice at same age group. *n*=5. (I) GO enrichment analysis reveals the related signaling pathways as indicated were enriched. The right panel represents enrichment of upregulated differentially expressed genes (DEGs). The left panel represents enrichment of downregulated DEGs. (J) Graphic presentation showing mRNA levels of T cell-related cytokines and chemokines. ^*^*P*<0.05, ^**^*P*<0.01 versus WT mice. *n*=5. (K, L) Western blot and quantification of WT1 in indicated groups. ^***^*P*<0.001 versus WT mice. (M) Graphs showing the mRNA level of WT1 in indicated groups. ^**^*P*<0.01 versus WT mice. (N) Representative images showing WT1 and Flag-tag co-staining in WT and Tg mice at 6 months of age. Bar=25 *μm*. (O) Images showing Flag-tag and β-catenin co-staining in 6-month-old WT mice. Bar=25 *μm*. (P) Images showing Hsp90ab1 staining in 6-month-old WT mice. Bar=25 *μm*. Full length original western blots for these results are provided in Supplementary File 1.

Fig. S3. Graph shows the relative mRNA level of B7-1 in different organs after injection of an shRNA vector encoding the interference sequence for B7-1 (B7-shR). ^*^*P*<0.05 versus control group. *n*=5. Male (8-weeks old) Balb/c mice were administrated with pLVX-shB7-1 or negative control vector by hydrodynamic-based gene delivery approach. Kidney, Liver, Heart and Lung were harvested from mice at 48 hours after injection.

Fig. S4. B7-1 knockdown retards glomerular damage in 5/6 nephrectomy models. (A) Experimental design. Arrows indicate injections of interference plasmids of B7-1 (shB7-1) or negative control (Ctl-shR) at indicated time points. Mice were first subjected to the two surgical resections of two thirds of the left kidney and the whole right kidney to establish 5/6 nephrectomy model. (B) Images showing podocyte ultrastructure by TEM analysis (Arrow indicates foot process fusion. Bar=1 *μm*), glomerular structure changes by PAS staining analysis (Arrow indicates mesangial expansion. Bar=20 *μm*). (C) Graphic presentation showing Ualb in different groups. ^**^*P*<0.01 versus Sham group; ^#^*P*<0.05 versus 5/6 nephrectomy mice. *n*=5. (D) Quantitative data showing the fraction of mesangial area quantified by PAS staining in indicated groups. ^**^*P*<0.01 versus sham group; ^##^*P*<0.01 versus 5/6 nephrectomy mice, *n*=5. (E) Representative images showing B7-1 immunofluorescence (IF) staining, Nephrin IF staining and α-SMA immunohistochemistry (IHC) staining in indicated groups. Bar=20 *μm*. (F-I) Western blot and qualification of B7-1, Synaptopodin and Nephrin in indicated groups. ^**^*P*<0.01 versus sham group; ^#^*P*<0.05 versus 5/6 nephrectomy mice, *n*=5. (J-M) Western blot and qualification of Fibronectin, Active β-catenin and PAI-1 in indicated groups. ^**^*P*<0.01 versus sham group; ^#^*P*<0.05 versus 5/6 nephrectomy mice. *n*=5. Full length original western blots for these results are provided in Supplementary File 1.

Fig. S5. B7-1 mediates podocyte injury through β-catenin signaling in vitro and glomerular mini-organ culture. (A) Quantitative data of B7-1, Active β-catenin, Zo-1, Synaptopodin, Nephrin, Fibronectin and Desmin are shown. MPC5 cells were transfected with B7-1 overexpression plasmid (pFlag-B7-1) or empty vector (pcDNA) for 24 hours. ^*^*P*<0.05, ^**^*P*<0.01 versus pcDNA group. *n*=3. (B) Quantitative data of Active β-catenin, Zo-1, Nephrin, Fibronectin, Desmin and WT1 in indicated groups are shown. MPC5 cells were pre-treated with ICG-001 (5 μmol/L) for one hour, following transfected with pFlag-B7-1 plasmid (or pcDNA) for another 24 hours. ^*^*P*<0.05, ^**^*P*<0.01 versus pcDNA group, ^#^*P*<0.05, ^##^*P*<0.01 versus B7-1 overexpression group. *n*=3. (C-E) Western blot and qualification of Zo-1, Podocalyxin, Nephrin and Desmin in isolated rat glomerulus are shown. Rat glomeruli were transduced with B7-1 overexpression lentivirus (Lenti-B7-1) or negative control (Lenti-NC). ^*^*P*<0.05 versus negative controls. *n*=3. (F-H) Graphs showing the mRNA levels of Zo-1, Synaptopodin and MMP7 in isolated rat glomeruli. ^*^*P*<0.05, ^**^*P*<0.05 versus negative controls. *n*=3. (I-N) Quantitative data of Flag, Zo-1, Podocalyxin, WT1, Fibronectin and Active β-catenin in isolated rat glomeruli are shown. The glomeruli were firstly pre-treated with ICG-001 (5 μmol/L) or DMSO, following transduced with B7-1 expressing lentivirus or negative control for another 72 hours. ^*^*P*<0.05, ^**^*P*<0.01 versus Lenti-NC+DMSO group, *ns*=no significance, ^#^*P*<0.05 versus Lenti-B7-1+DMSO group. *n*=3. (O, P) Establishment diagram of B7-1^flox/flox^ mice is shown. Genotyping was confirmed by PCR analysis. (Q, R) Quantitative data of glomerular Nephrin and Active β-catenin in isolated B7-1^flox/flox^ mouse glomeruli with different treatment are shown. The isolated B7-1^flox/flox^ mouse glomeruli were treated with adenovirus carrying NPHS2-driven Cre recombinase (AdV-NPHS2-Cre) or negative control for 48 hours and followed administered with ADR (0.125 μg/ml) for another 24 hours. ^*^*P*<0.05, ^**^*P*<0.01 versus AdV-NC group, ^#^*P*<0.05 versus ADR+AdV-NC group. *n*=3. Full length original western blots for these results are provided in Supplementary File 1.

Fig. S6. Hsp90ab1 mediates podocyte injury and glomerular damage in ADR mice. (A) Representative images showing co-expression of B7-1 and α-actinin-4 in mice treated with ADR for 7 days. Arrows indicate specific co-localization. Bar=25 *μm.* (B-E) Quantitative data of LRP5, Hsp90ab1, Active β-catenin and B7-1 in indicated group are shown. ^*^*P*<0.05, ^**^*P*<0.01 versus controls. ns=no significance, ^##^*P*<0.01 versus ADR group. *n*=5. (F, G) Quantitative data of β-catenin and B7-1 positive staining in glomerulus are shown. ^**^*P*<0.01 versus controls. ns=no significance, ^##^*P*<0.01 versus ADR group. *n*=5. (H) Quantitative data of Podocalyxin in indicated group are shown. ^**^*P*<0.01 versus controls. ns=no significance, ^##^*P*<0.01 versus ADR group. *n*=5. (I-M) Quantitative data of Hsp90ab1, LRP5, Active β-catenin, β-catenin and B7-1 in ADR models and ADR mice treated with Hsp90ab1-shR or its negative control (Ctl-shR) are shown. ^**^*P*<0.01 versus controls, ^##^*P*<0.01 versus ADR+Ctl-shR group. *n*=5. (N-P) Quantitative data of Podocalyxin, Nephrin and Fibronectin, in indicated group are shown. ^*^*P*<0.05, ^**^*P*<0.01 versus controls, ^#^*P*<0.01, ^##^*P*<0.01 versus ADR+Ctl-shR group. *n*=5.

Fig. S7. LRP5 is increased in podocytes in clinical glomerular nephropathy. (A) Graphs showing co-staining of LRP5 and podocalyxin in frozen kidney sections from LN patient. Bar=25 *μm*. (B) Representative micrographs showing the immunochemistry staining of LRP5 in paraffin-embedded sections from indicated patients. Bar=50 *μm*.

Fig. S8. B7-1 is also involved in podocyte cell apoptosis and B7-1-mediates integrin signaling through Hsp90ab1.

**(A)** GSEA enrichment analyses show apoptosis-related signaling were upregulated in ADR-treated MPC5 cells but this was downregulated by B7-1 interference. MPC5 cells were transfected with siRNA to B7-1 (siB7-1) or negative control (si-NC) for 24 hours, following treatment with 0.25μg/ml of ADR for another 24 hours. **(B-D)** Western blot and quantitative data of PARP-1 and Cleaved caspase 3 in indicated groups are shown. ^*^*P*<0.05 ^**^*P*<0.01 versus MPC5 cells treated with siNC, ^#^*P*<0.05 versus MPC5 cells treated with ADR and siNC. *n=3.* **(E)** GO enrichment bar plot for differential genes related with integrin in indicated group. The result reveals that integrin-related pathways were enriched by B7-1, and integrin-mediated cell adhesion was significantly engaged. **(F)** Gene expression profiling of RNA-seq in cultured MPC5 cells showing integrin-related genes in indicated groups. **(G)** GSEA enrichment analysis shows that integrin β1 signaling was downregulated in kidney tissue from Tg mice compared to WT mice. NES, normalized enrichment. **(H)** Protein interaction analysis showing intimate connection among integrin β1 (ITGB1), β-catenin (CTNNB1), Hsp90ab1 (HSP90AB1), and podocyte specific markers such as podocalyxin (PODX), synaptopodin (SYNPO) and others. **(I)** Representative immunoblotting show overexpression of B7-1 in podocytes promoted the binding of B7-1 with integrin β1 and inhibited the integrin β1 signaling, while interference of Hsp90ab1 largely interrupted their interaction and restored integrin β1 signaling. MPC5 cells were transfected with empty vector, B7-1 expression plasmid (pFlag- B7-1) alone or co-transfected with siRNA to Hsp90ab1 (siHsp90ab1) for 24 hours. The cell lysates were immunoprecipitated with antibody against B7-1 or integrin β1. Total diluted lysates were used as input. IP, immunoprecipitation. Experiments were performed in duplicate and repeated three times. **(J)** Graphic presentations showing the molecular structure of integrin β1 from homology modeling and the putative binding model of B7-1, integrin β1 and Hsp90ab1 dimer. **(K)** Schematic presentation depicts the underlying mechanisms of B7-1 in mediating glomerulosclerosis and podocyte injury. On one hand, the overexpression of B7-1 may recruit and interact with Hsp90ab1, leading to the activation of β-catenin signaling; On the other hand, B7-1 could bind to integrin β1 and disrupt its function, resulting in cell migration and cytoskeleton reorganization. Full length original western blots for these results are provided in Supplementary File 1.

Fig. S9. (A, B) Graphs show mRNA levels of β-catenin and B7-1 in two groups. HUVEC cells were transfected with pDel-β-catenin plasmid or pcDNA plasmid (24 h). B7-1 mRNA level was not significantly induced by β-catenin overexpression.^***^*P*<0.001 versus pcDNA group, *n*=3. (C, D) Graphs show mRNA levels of β-catenin and B7-1 in two groups. RMC cells were transfected with pDel-β-catenin plasmid or pcDNA plasmid (24 h). B7-1 mRNA level was not significantly induced by β-catenin overexpression. ^*^*P*<0.05 versus pcDNA group, *n*=3.

**Supplementary Tables**

Table S1. Demographic and clinical data of human urinary.

|  | **No.** | **Gender*** | **Age** | **pathological diagnosis ^#^** | **Urinary B7-1(ng/g Ucr.)** | **eGFR(ml/min/1.73m^2)** | **ACR(mg/g)** | **Urinary β2-MG(mg/L)** |
| --- | --- | --- | --- | --- | --- | --- | --- | --- |
| Healthy Adults | 1 | M | 52 | No | 27.5602 | 106 | 9.9822 | 0.183 |
|  | 2 | F | 38 | No | 24.6923 | 116 | 4.4601 | 0.183 |
|  | 3 | M | 42 | No | 32.5896 | 117 | 0.5980 | 0.183 |
|  | 4 | F | 32 | No | 32.5908 | 113 | 2.9940 | 0.183 |
|  | 5 | M | 30 | No | 45.4306 | 108 | 1.1076 | 0.183 |
|  | 6 | M | 45 | No | 25.5355 | 105 | 6.3788 | 0.183 |
|  | 7 | M | 42 | No | 25.9993 | 103 | 0.8860 | 0.183 |
|  | 8 | M | 39 | No | 39.1895 | 113 | 3.1530 | 0.183 |
|  | 9 | F | 24 | No | 33.0430 | 123 | 0.3465 | 0.183 |
|  | 10 | F | 39 | No | 25.9073 | 111 | 0.7822 | 0.183 |
| Patients | 1 | F | 46 | MN | 96.7127 | 8 | 1309.1414 | 33.600 |
|  | 2 | M | 43 | IgAN | 70.3788 | 13 | 376.1200 | 0.183 |
|  | 3 | F | 33 | DN | 107.1579 | 22 | 3260.3830 | 0.183 |
|  | 4 | M | 42 | MN | 69.7507 | 7 | 290.5830 | 12.600 |
|  | 5 | F | 59 | LN | 192.4479 | 5 | 766.3527 | 18.700 |
|  | 6 | F | 41 | IgAN | 141.6038 | 15 | 3414.1962 | 1.5100 |
|  | 7 | M | 53 | DN | 32.8351 | 16 | 199.4628 | 0.183 |
|  | 8 | M | 64 | ANCA | 162.2288 | 23 | 1.9776 | 0.216 |
|  | 9 | F | 33 | IgAN | 60.2319 | 62.21 | 7110.2330 | 0.456 |
|  | 10 | F | 32 | LN | 68.4190 | 32.4 | 1332.7255 | 0.183 |
|  | 11 | F | 35 | IgAN | 11.2041 | 116 | 143.2051 | 0.215 |
|  | 12 | M | 43 | FSGS | 88.3334 | 35.93 | 81.2436 | 0.183 |
|  | 13 | M | 47 | MN | 25.9173 | 77.81 | 4820.2276 | 0.183 |
|  | 14 | M | 50 | MN | 56.2856 | 71.78 | 4423.2052 | 0.272 |
|  | 15 | F | 24 | LN | 54.4993 | 22.2 | 1507.3678 | 0.183 |
|  | 16 | M | 45 | IgAN | 46.4130 | 44.17 | 3844.5362 | 41.700 |
|  | 17 | M | 54 | MN | 59.9061 | 95.85 | 5934.3384 | 0.296 |
|  | 18 | M | 21 | IgAN | 26.3488 | 99.44 | 478.7207 | 0.819 |
|  | 19 | F | 50 | IgAN | 73.6277 | 55.87 | 780.3655 | 0.183 |
|  | 20 | F | 54 | IgAN | 53.5345 | 77.85 | 555.1544 | 3.860 |
|  | 21 | M | 28 | IgAN | 9.0860 | 94.67 | 267.4327 | 0.367 |
|  | 22 | F | 34 | IgAN | 34.4674 | 112.78 | 1240.2563 | 0.183 |
|  | 23 | F | 31 | LN | 76.0874 | 22.77 | 9029.8341 | 0.183 |
|  | 24 | F | 60 | MN | 79.5307 | 95.66 | 2749.1206 | 4.730 |
|  | 25 | M | 17 | MCD | 40.5161 | 127.51 | 4799.8329 | 0.183 |
|  | 26 | M | 29 | IgAN | 207.8399 | 90.51 | 5250.4658 | 0.183 |
|  | 27 | F | 54 | MN | 74.3121 | 70.93 | 2022.1265 | 0.639 |
|  | 28 | F | 31 | IgAN | 27.9462 | 110.84 | 302.0413 | 0.183 |
|  | 29 | M | 28 | IgAN | 15.9400 | 101.14 | 195.1435 | 0.183 |
|  | 30 | F | 47 | IgAN | 41.0958 | 49.82 | 3807.5342 | 1.380 |
|  | 31 | F | 55 | MN | 64.0422 | 95.45 | 2679.5563 | 1.540 |
|  | 32 | F | 37 | IgAN | 46.5172 | 111.81 | 122.0002 | 0.183 |
|  | 33 | F | 28 | IgAN | 53.6618 | 83.89 | 2635.1302 | 0.183 |
|  | 34 | M | 32 | IgAN | 19.6212 | 94.47 | 7.4928 | 0.183 |
|  | 35 | M | 32 | IgAN | 17.1039 | 42.25 | 119.4793 | 0.183 |
|  | 36 | F | 30 | IgAN | 29.3412 | 120.86 | 248.9620 | 0.183 |
|  | 37 | F | 37 | IgAN | 56.3169 | 134.79 | 179.9477 | 0.183 |
|  | 38 | M | 25 | MN | 113.5452 | 118.63 | 1107.3273 | 0.214 |
|  | 39 | F | 23 | IgAN | 56.0217 | 34.28 | 3847.3504 | 0.589 |
|  | 40 | M | 14 | FSGS | 62.2437 | 158.66 | 443.0634 | 0.183 |
|  | 41 | F | 47 | MN | 78.0583 | 79.21 | 10439.0293 | 0.183 |
|  | 42 | M | 32 | IgAN | 65.3804 | 110.2 | 454.2418 | 0.304 |
|  | 43 | M | 29 | MCD | 22.5867 | 61.24 | 559.7561 | 0.212 |
|  | 44 | M | 64 | IgAN | 39.4041 | 34.21 | 6113.8322 | 1.400 |
|  | 45 | F | 35 | MN | 64.5994 | 124.65 | 293.7082 | 0.183 |
|  | 46 | M | 48 | MN | 70.5882 | 55.07 | 6237.5235 | 0.375 |
|  | 47 | M | 21 | MCD | 82.6160 | 133.05 | 1471.1216 | 3.500 |
|  | 48 | M | 48 | IgAN | 35.7005 | 54.57 | 133.9394 | 0.183 |
|  | 49 | F | 30 | IgAN | 68.8907 | 46.52 | 2508.7899 | 1.550 |
|  | 50 | M | 53 | IgAN | 43.9252 | 80.46 | 1645.2641 | 0.183 |
|  | 51 | F | 23 | MN | 28.5001 | 140.97 | 7006.2965 | 0.183 |
|  | 52 | F | 59 | MN | 61.8456 | 76.39 | 689.7452 | 0.295 |
|  | 53 | F | 79 | MN | 90.8910 | 37 | 23465.4462 | 0.371 |
|  | 54 | F | 22 | MN | 23.3408 | 127.09 | 240.2431 | 1.230 |
|  | 55 | M | 32 | MsGN | 22.5833 | 84.39 | 115.0568 | 1.270 |
|  | 56 | M | 38 | IgAN | 37.1855 | 73.79 | 66.0998 | 0.183 |
|  | 57 | M | 32 | IgAN | 49.8105 | 105.48 | 632.9231 | 0.183 |
|  | 58 | M | 54 | MCD | 50.7799 | 38.53 | 8754.1748 | 0.183 |
|  | 59 | M | 47 | MN | 128.2610 | 101.65 | 7543.6157 | 4.560 |
|  | 60 | M | 54 | MN | 27.1749 | 102.31 | 427.9863 | 2.270 |
|  | 61 | M | 24 | MN | 34.9288 | 102.63 | 1657.2139 | 0.185 |
|  | 62 | M | 21 | MCD | 45.1850 | 106.24 | 14626.6805 | 0.183 |
|  | 63 | F | 45 | IgAN | 36.5084 | 96.79 | 92.6081 | 0.183 |
|  | 64 | F | 27 | IgAN | 55.8924 | 107.86 | 204.9568 | 0.183 |
|  | 65 | F | 46 | FSGS | 41.9929 | 83.7 | 684.7722 | 0.183 |
|  | 66 | M | 74 | FSGS | 71.8672 | 7.58 | 15227.7657 | 0.257 |
|  | 67 | M | 56 | MN | 39.5599 | 93.1 | 2795.5650 | 8.120 |
|  | 68 | M | 28 | IgAN | 35.0188 | 115.05 | 568.7215 | 0.183 |
|  | 69 | M | 44 | MN | 130.0016 | 72.34 | 9841.7495 | 0.247 |
|  | 70 | M | 55 | MN | 32.6654 | 78.32 | 1532.7005 | 0.183 |
|  | 71 | M | 27 | MN | 30.5219 | 120.84 | 200.1240 | 0.773 |
|  | 72 | M | 59 | DN | 85.1098 | 80.26 | 4143.2722 | 0.183 |
|  | 73 | M | 26 | IgAN | 34.9817 | 80.28 | 483.8759 | 0.183 |
|  | 74 | F | 42 | C3GN | 39.8176 | 77.17 | 160.2201 | 20.600 |
|  | 75 | F | 35 | IgAN | 44.5473 | 77.57 | 1297.1739 | 0.183 |
|  | 76 | F | 34 | IgAN | 44.5980 | 91.06 | 1017.7417 | 0.183 |

*M, male; F, female. ^#^ MN, Membrane nephropathy; IgAN, IgA nephropathy; LN, Lupus nephritis; MCD, Minimal change disease; DN, Diabetes nephropathy; FSGS, Focal segmental glomurular sclerosis; C3GN, C3 glomerulonephritis. 0.183 mg/L is the minimum value of β2-MG assessed by the reagent kit.

Table S2. Primary antibodies, chemical reagents and primer sequences.

| **Primary Antibodies** | | | |
| --- | --- | --- | --- |
|  |  |  |  |
| **Target** | **Host** | **Application** | **Supplier** |
| B7-1 | Rb | WB | abcam, ab215116 |
| B7-1 | Ms | IF | Genetex, GTX84700 |
| B7-1 | Rb | IF | Abclonal, A16039 |
| B7-1 | Amernican Hamster | IP | ebioscience,2119696 |
| α-actinin-4 | Rb | IF | Enzo life sciences, ALX-210-356-C050 |
| Flag | Ms | WB/IF/IP | MBL, M185-3 |
| Flag | Rb | IF | Proteintech, 20543-1-AP |
| HA | Ms | IP | MBL, M180-3 |
| Nephrin | Rb | WB | Abcam, ab58968 |
| Nephrin | Guinea pig | IF | Fitzgerald,20R-NP002 |
| Podocalyxin | Goat | WB/IF | R&D, AF1556 |
| Synaptopodin | Ms | WB | Santa Cruz, sc515842 |
| WT1 | MS | WB | Santa Cruz, sc393498 |
| WT1 | Rb | IHC | Abcam, ab89901 |
| Zo-1 | Rb | WB/ICC | Invitrogen,40-2200 |
| Collagen IV | Rb | WB/IHC | Abcam, ab6586 |
| Fibronectin | Rb | WB/IHC/IF | Sigma, F3648 |
| Active β-catenin | Rb | WB/ICC | CST,19807S |
| PAI-1 | Goat | WB | R&d, AF3828 |
| β-catenin | Ms | WB/IHC/IF | BD,610154 |
| β-catenin | Rb | IF | Abclonal, A19657 |
| Desmin | Rb | WB | Boster, PB9105 |
| Hsp90ab1 | Rb | WB/IP | Abcam, ab203085 |
| Hsp90ab1 | Rb | IF | Proteintech, 11405-1-AP |
| LRP5 | Rb | WB | CST,5731 |
| LRP5 | Rb | IIHC/F | Abclonal,A0130 |
| LEF1 | Rb | ChIP | CST,2230 |
| TCF1 | Rb | ChIP | CST,2203 |
| TCF4 | Rb | ChIP | CST,2569 |
| α-SMA | Ms | IHC | Sigma, A2547 |
| PARP-1 | Rb | WB | CST,9542S |
| Cleave caspase 3 | Rb | WB | CST, 9664S |
| Integrin β1 | Rb | WB | Abcam, Ab183666 |
| Integrin β1 | Rb | IP | Proteintech, 12594-1-AP |
| **Chemical reagents, Recombinant proteins and siRNA sequences** | | | |
|  |  |  |  |
| Name | | Supplier | |
| Adrimycin | | Mym Biological Technology Company, MD0071 | |
| ICG-001 | | ChemBest Research Laboratories Ltd. 847591-62-2 | |
| Tanespimycin (17-AAG) | | Selleck, S1141 | |
| rhWnt3a | | R&D, RSK8920041 | |
| B7-1-siRNA | | CCGUUACAACUCUCCUCAUTT  AUGAGGAGAGUUGUAACGGTT | |
| LRP5 siRNA | | CCAUCCAUGCCUGCAACAATT  UUGUUGCAGGCAUGGAUGGTT | |
| Hsp90ab1-siRNA | | GGAGAAGCCUAAGAUUGAATT  UUCAAUCUUAGGCUUCUCCTT | |
| **Primers** | | | |
|  |  |  |  |
| Transgene PCR primer1 | | F | TATCTCCGTCAGCCCTCTAGC |
|  |  | R | GTCCGGTTCTTATACTCGGGC |
| Transgene PCR primer2 | | F | CCTTGACTACAAAGACCATGACGG |
|  |  | R | CAGATGCTCAAGGGGCTTCAT |
| B7-1^flox/flox^ mice Primer 1 | | F | GTATTGAGCAAATGATGGGGAGCA |
|  |  | R | CACTTCTGCTACCAATTCCTATCCT |
| B7-1^flox/flox^ mice Primer 1 | | F | GCGCTGCTGCTCCAG |
|  |  | R | CGGTTATTCAACTTGCACCA |
| Mouse B7-1 | | F | TGCCTTGCCGTTACAACTCT |
|  |  | R | GTATGTGCCCCGGTCTGAAA |
| Mouse Fibronectin | | F | ATGTGGACCCCTCCTGATAGT |
|  |  | R | GCCCAGTGATTTCAGCAAAGG |
| Mouse Desmin | | F | GTTTCAGACTTGACTCAGGCAG |
|  |  | R | TCTCGCAGGTGTAGGACTGG |
| ChIP(TCFs/LEF) Binding site 1 | | F | ACTGTGACGGCTAGGAGCAT |
|  |  | R | AAGACCTCGGAGGAAGCTCC |
| ChIP(TCFs/LEF) Binding site 2 | | F | TCCGAGGTCTTAGTAAAGGG |
|  |  | R | CTGAAAGTTTGAGGCCAGCC |
| ChIP(TCFs/LEF) Binding site3 | | F | CAGGCTGGCCTCAAACTTTCA |
|  |  | R | TGCCGCAGCCTTGAGAATAAG |
