# Supplementary figures and images for: B7-1 mediates podocyte injury and glomerulosclerosis through communication with Hsp90ab1-LRP5-β-catenin pathway
Source: Cell Death Differ. 2022 Jun 16;29(12):2399–416. doi: 10.1038/s41418-022-01026-8 (PMC9750974; doi:10.1038/s41418-022-01026-8)

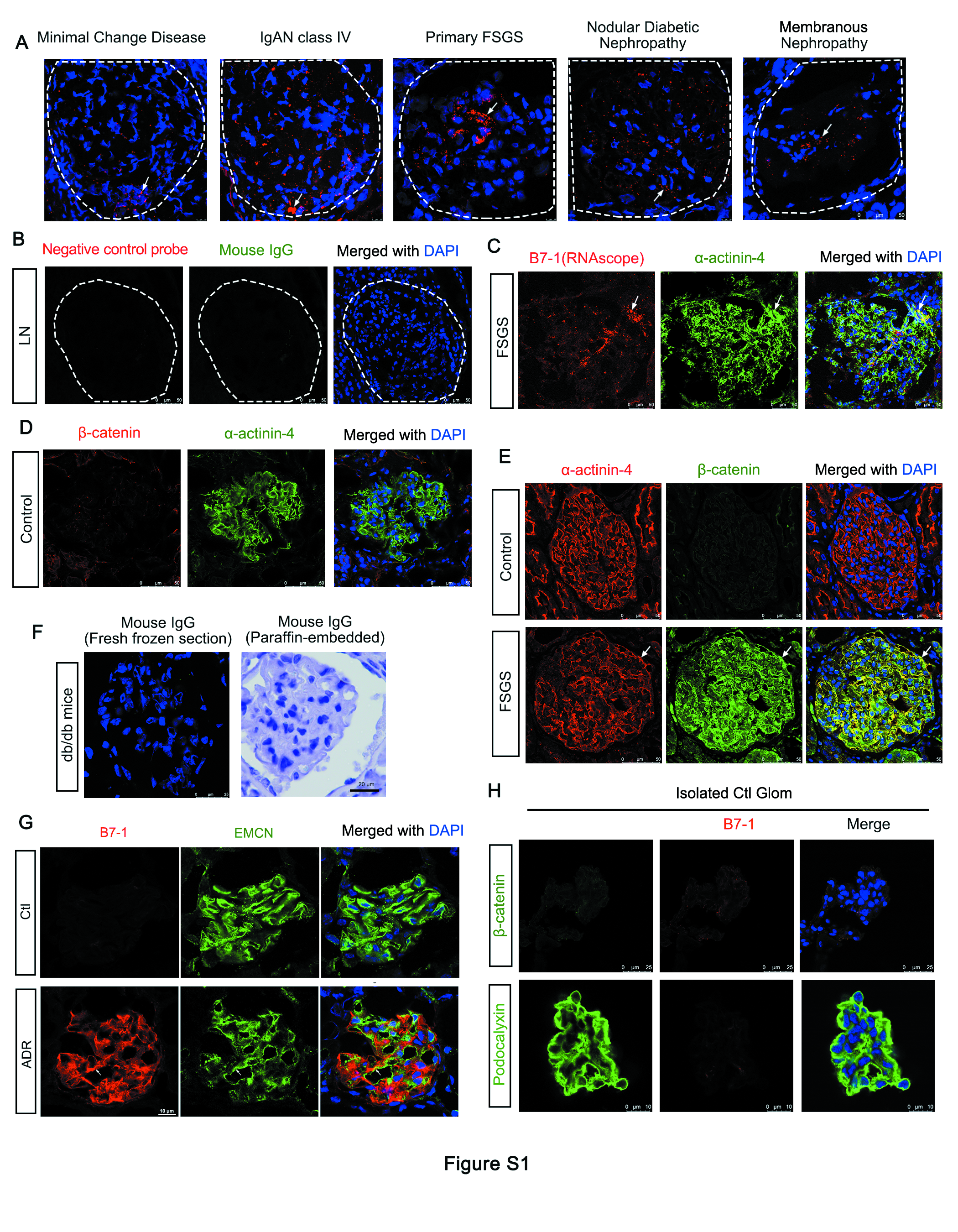

Supplement: Supplementary file 4 — Supplementary Figure S1 [file 41418_2022_1026_MOESM4_ESM.tif]

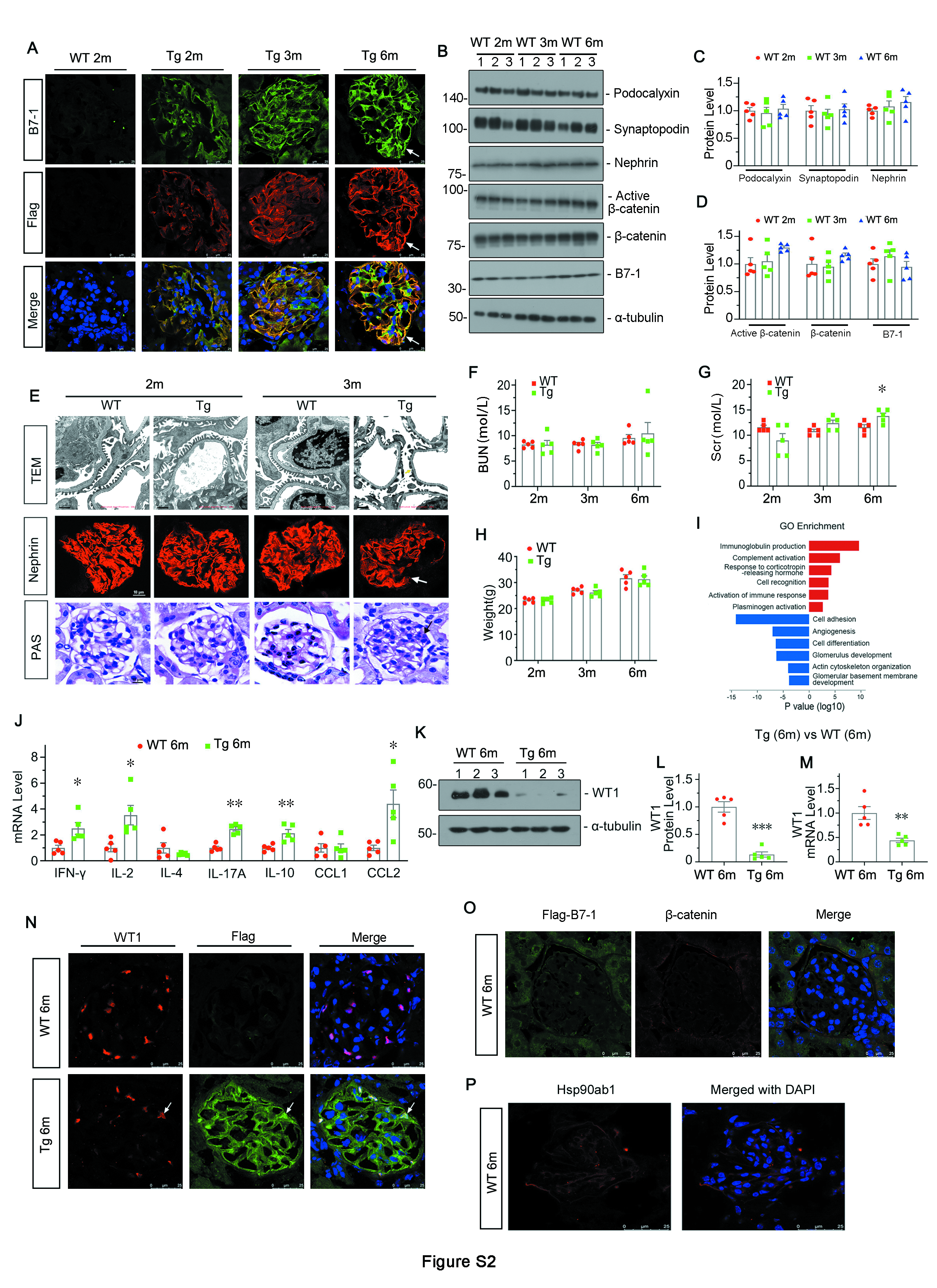

Supplement: Supplementary file 5 — Supplementary Figure S2 [file 41418_2022_1026_MOESM5_ESM.tif]

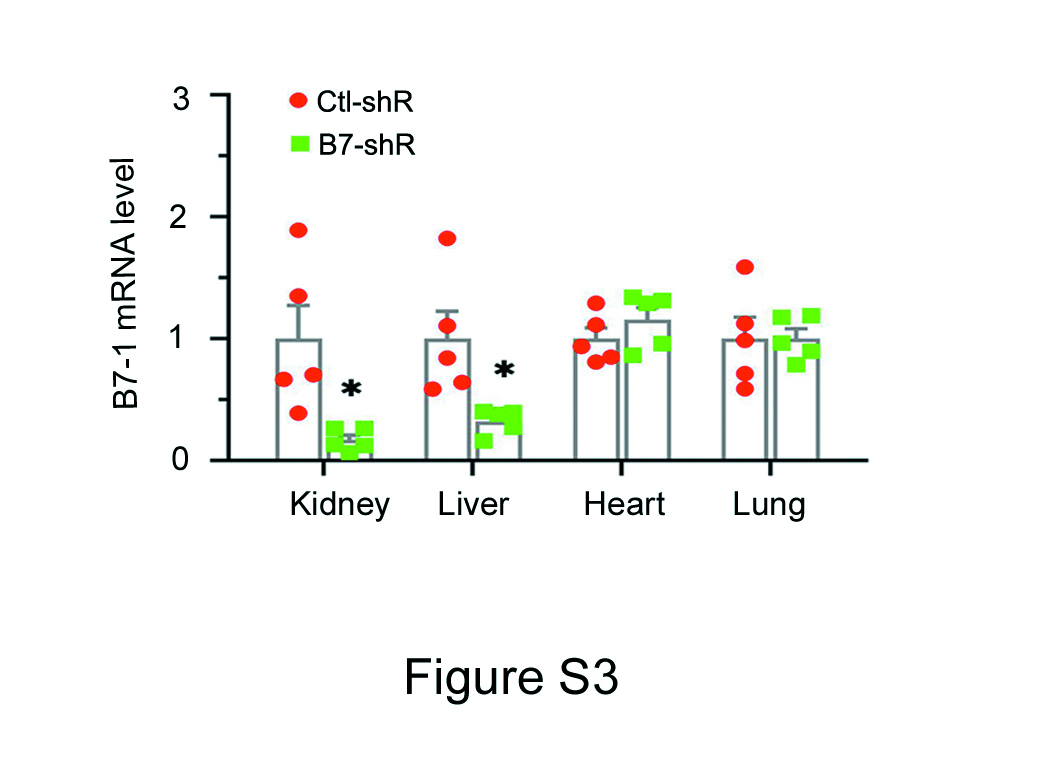

Supplement: Supplementary file 6 — Supplementary Figure S3 [file 41418_2022_1026_MOESM6_ESM.tif]

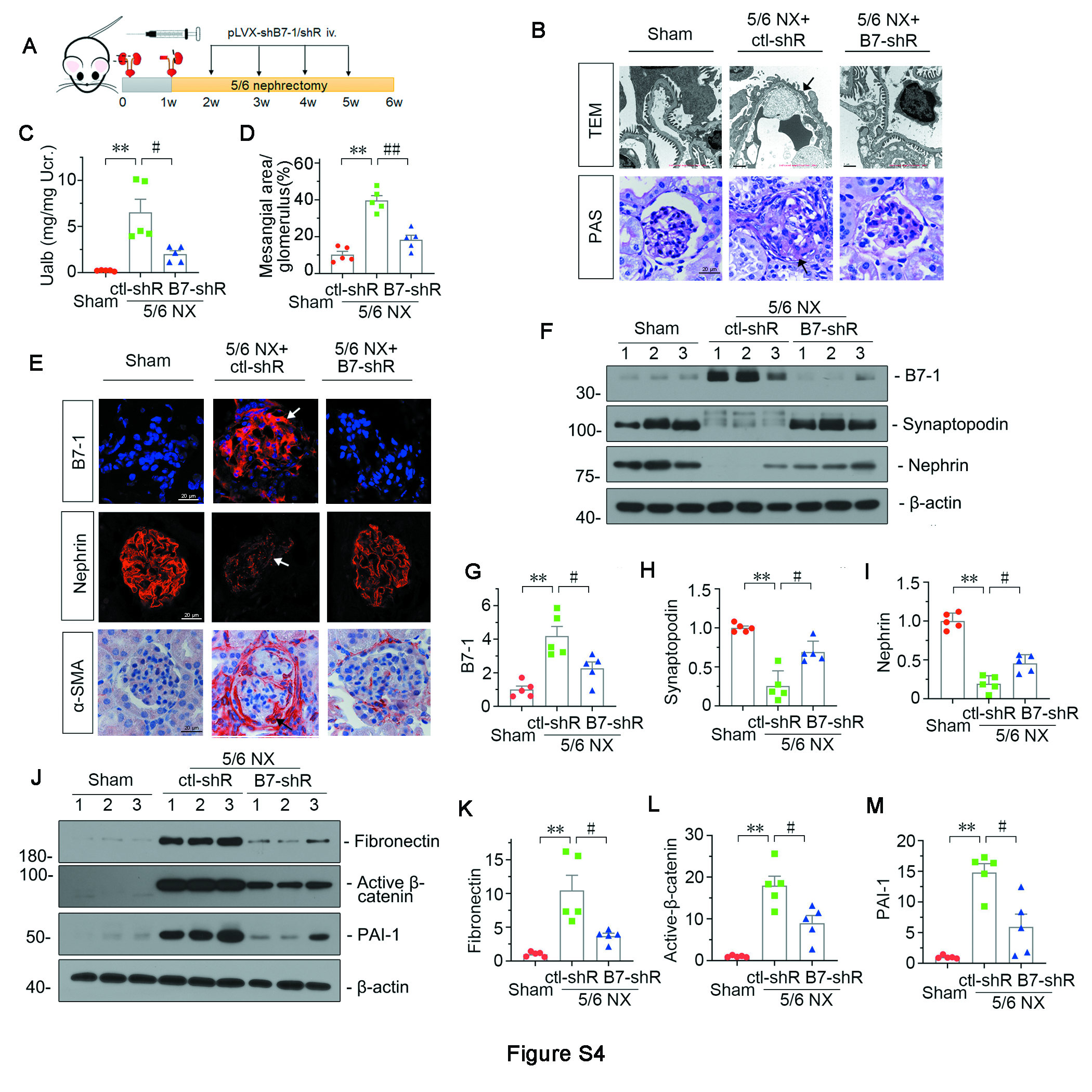

Supplement: Supplementary file 7 — Supplementary Figure S4 [file 41418_2022_1026_MOESM7_ESM.tif]

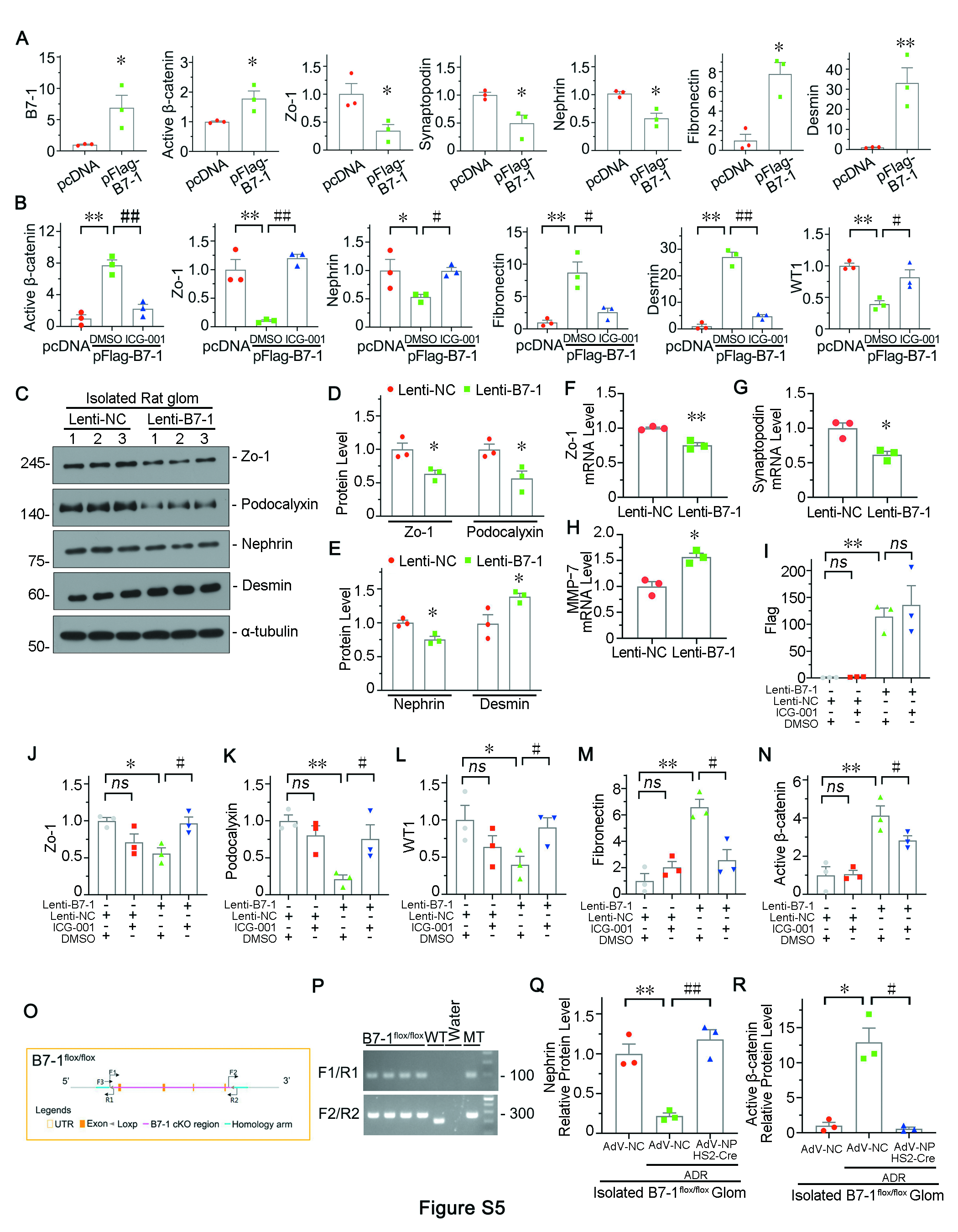

Supplement: Supplementary file 8 — Supplementary Figure S5 [file 41418_2022_1026_MOESM8_ESM.tif]

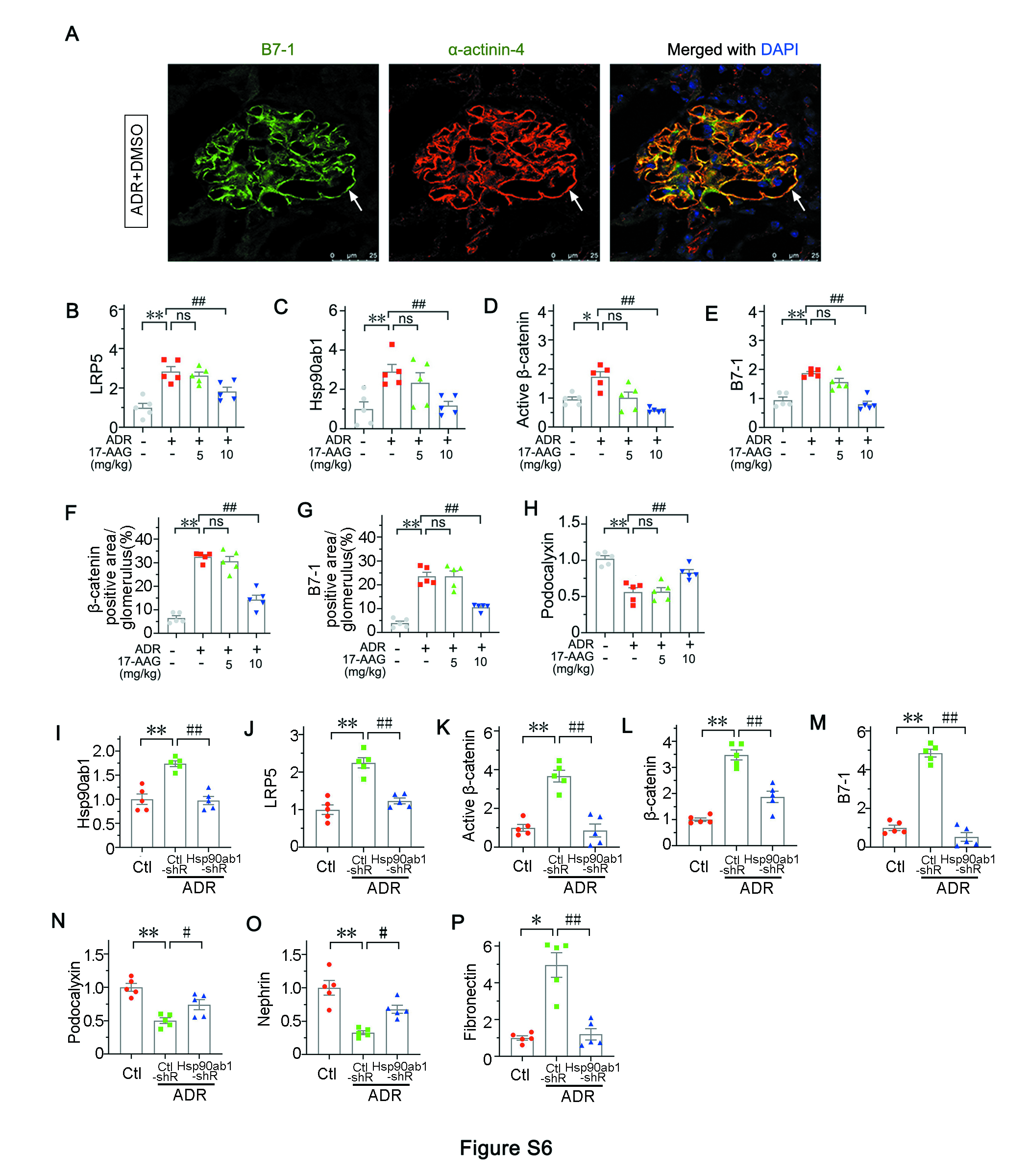

Supplement: Supplementary file 9 — Supplementary Figure S6 [file 41418_2022_1026_MOESM9_ESM.tif]

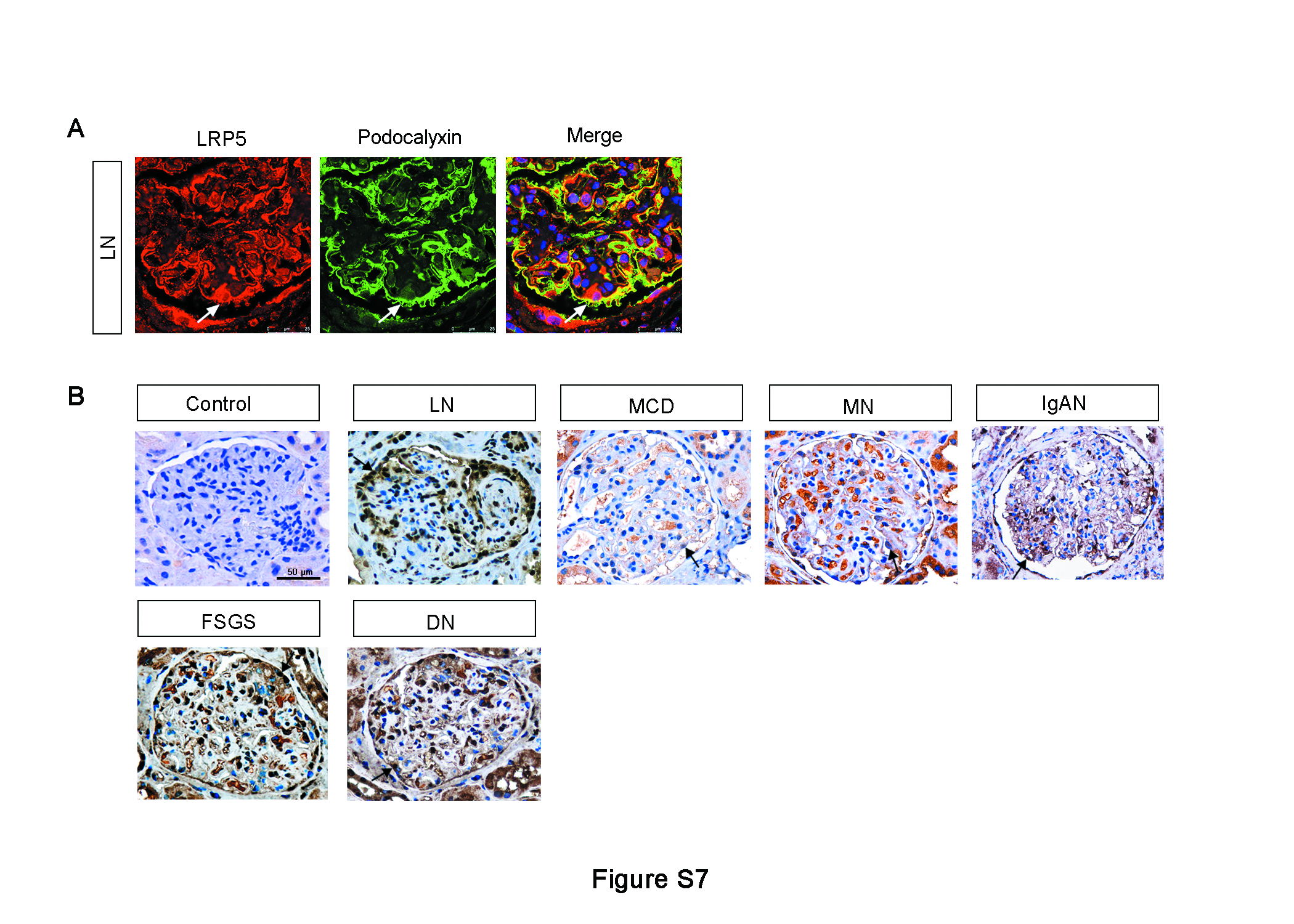

Supplement: Supplementary file 10 — Supplementary Figure S7 [file 41418_2022_1026_MOESM10_ESM.tif]

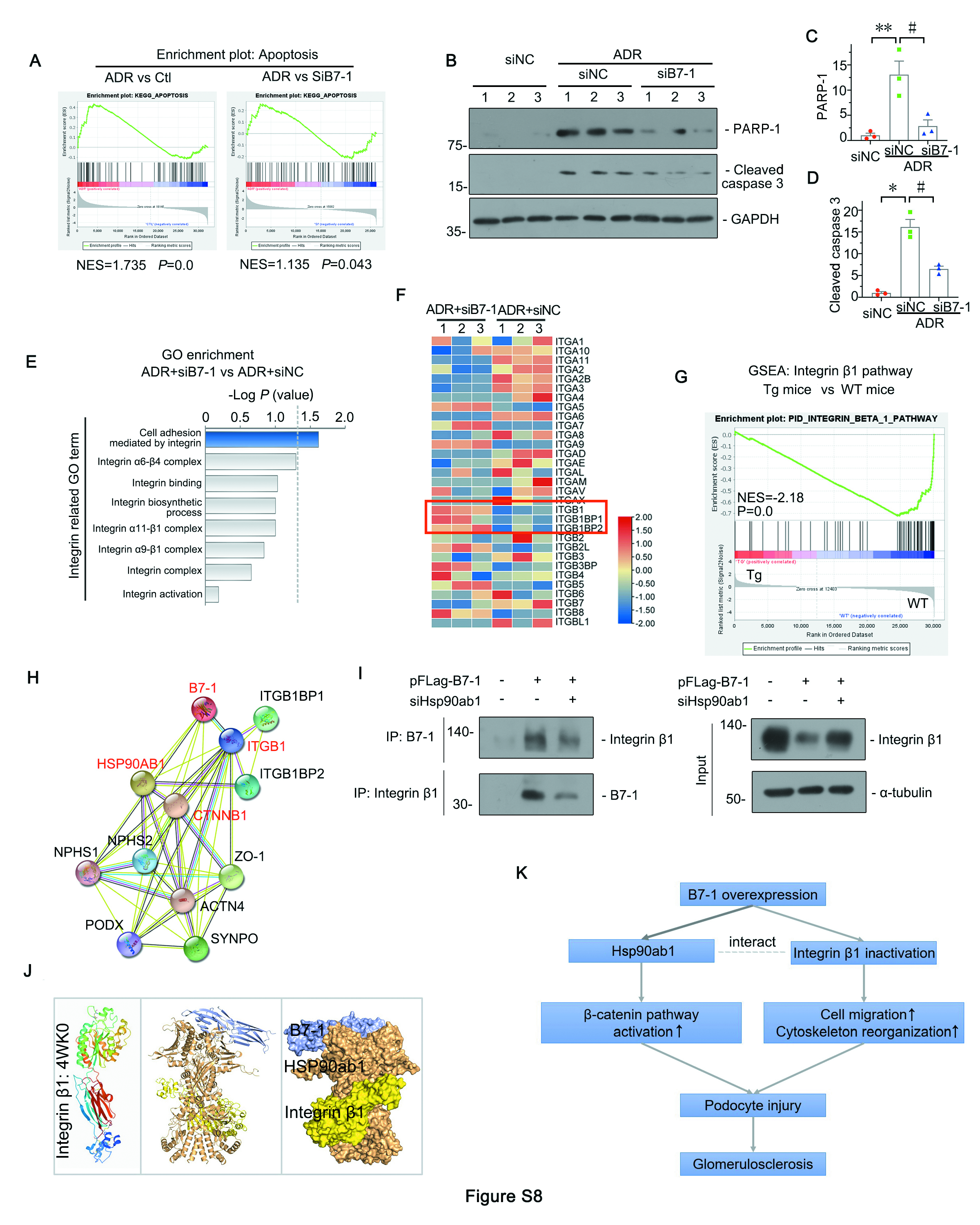

Supplement: Supplementary file 11 — Supplementary Figure S8 [file 41418_2022_1026_MOESM11_ESM.tif]

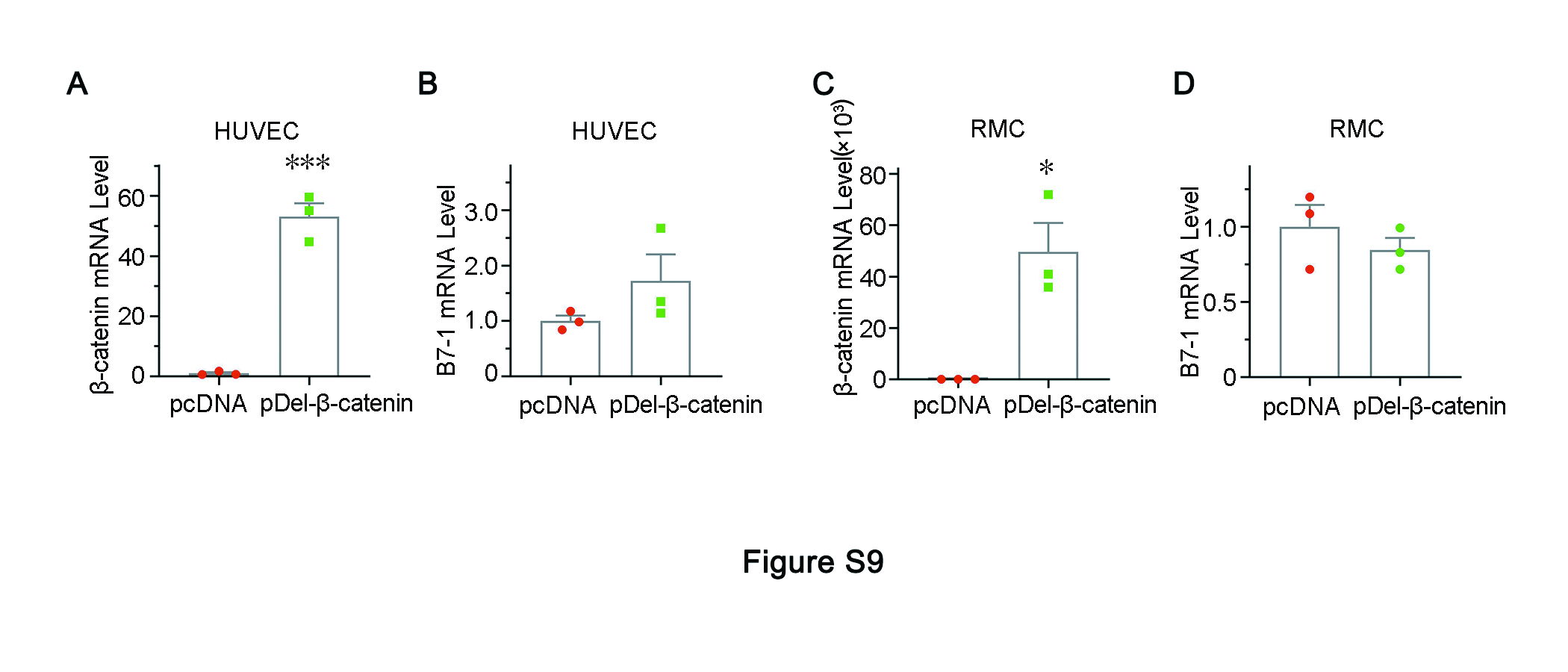

Supplement: Supplementary file 12 — Supplementary Figure S9 [file 41418_2022_1026_MOESM12_ESM.tif]
