# Supplementary material for: B7-1 mediates podocyte injury and glomerulosclerosis through communication with Hsp90ab1-LRP5-β-catenin pathway
Source: Cell Death Differ. 2022 Jun 16;29(12):2399–416. doi: 10.1038/s41418-022-01026-8 (PMC9750974; doi:10.1038/s41418-022-01026-8)

Full unedited gels for Figure 1

Fig. 1J

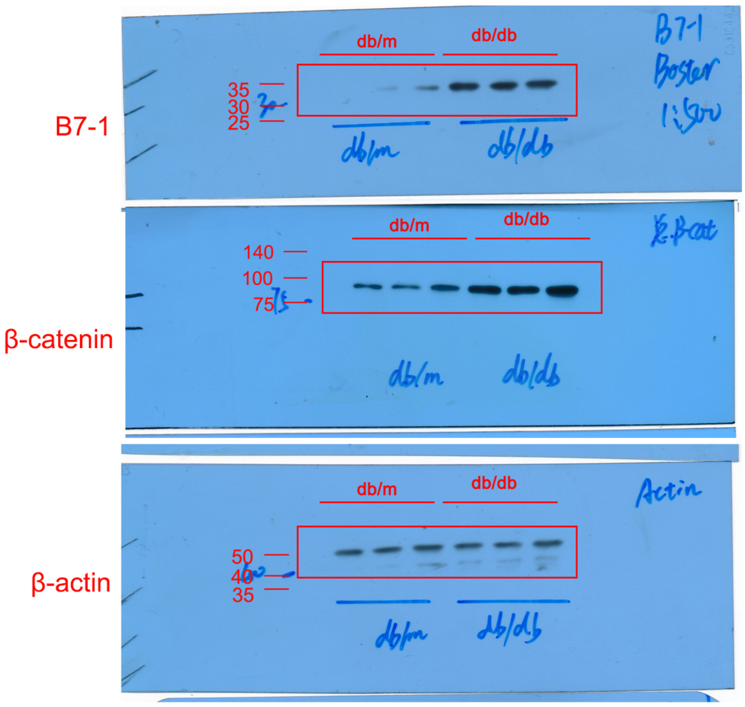

Fig. 1M

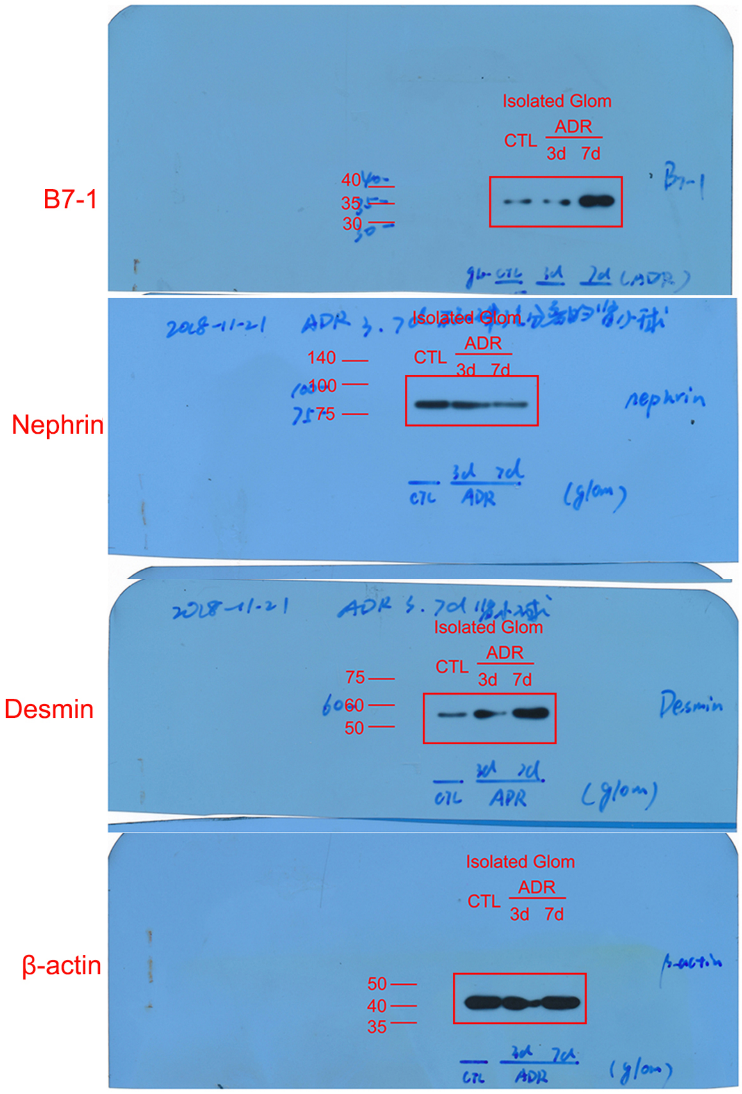

Full unedited gels for Figure 2

Fig. 2B

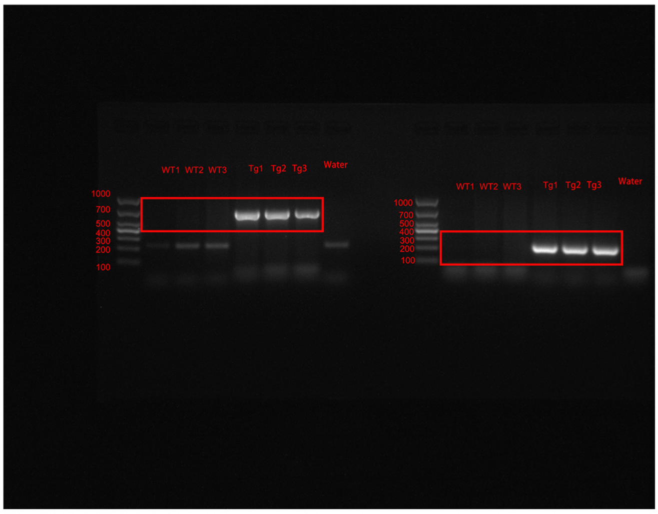

Fig. 2E

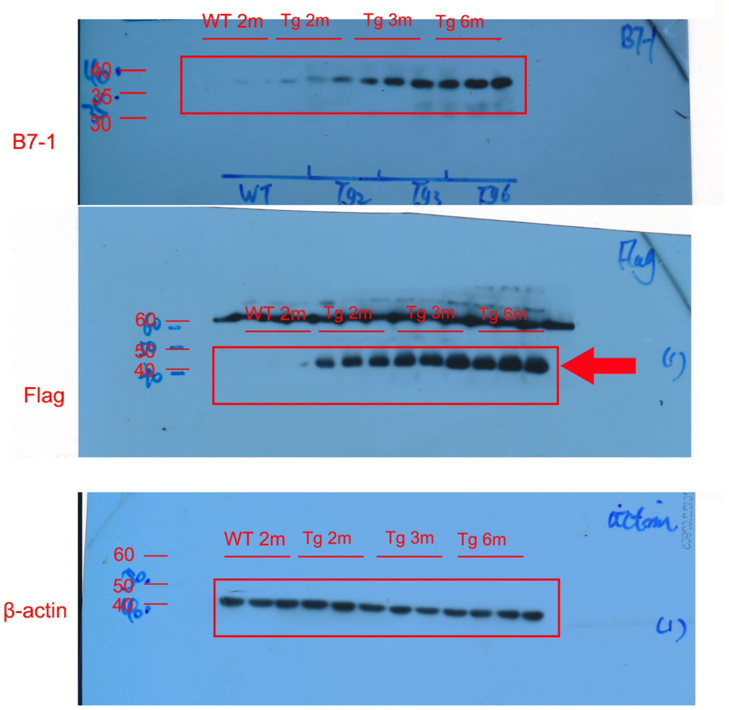

Fig. 2K

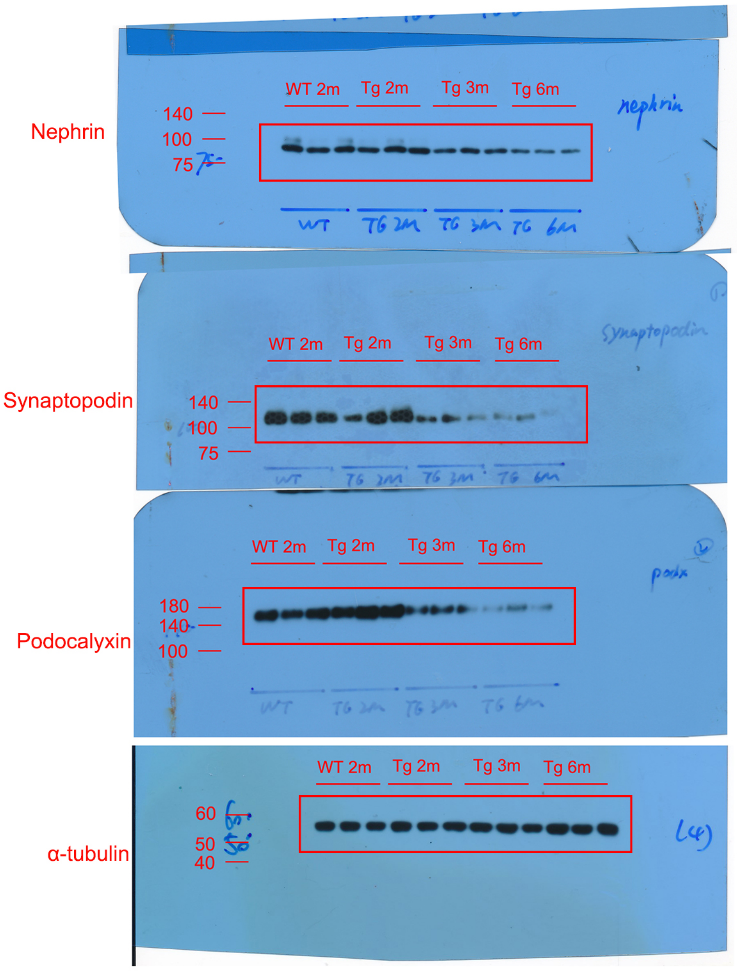

Fig. 2P

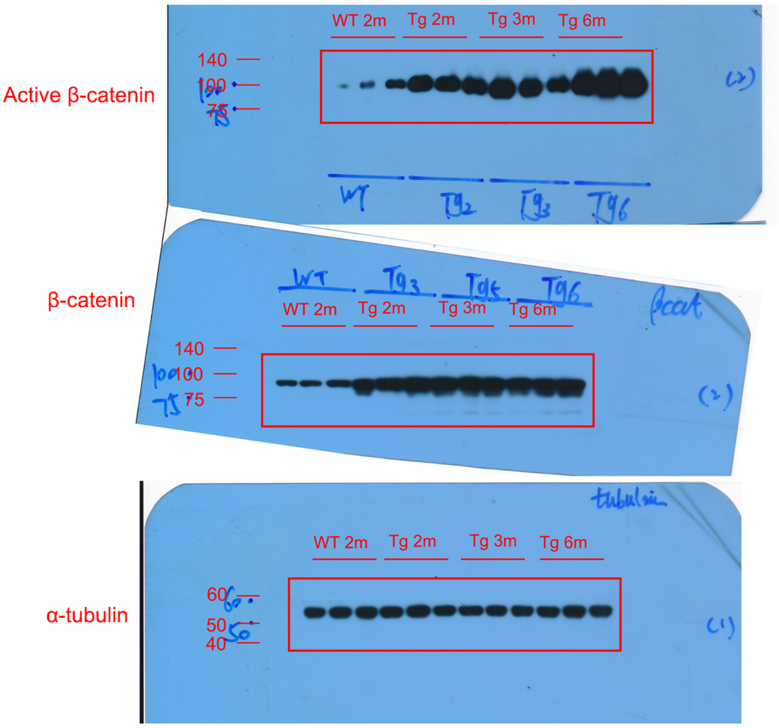

Full unedited gels for Figure 3

Fig. 3G

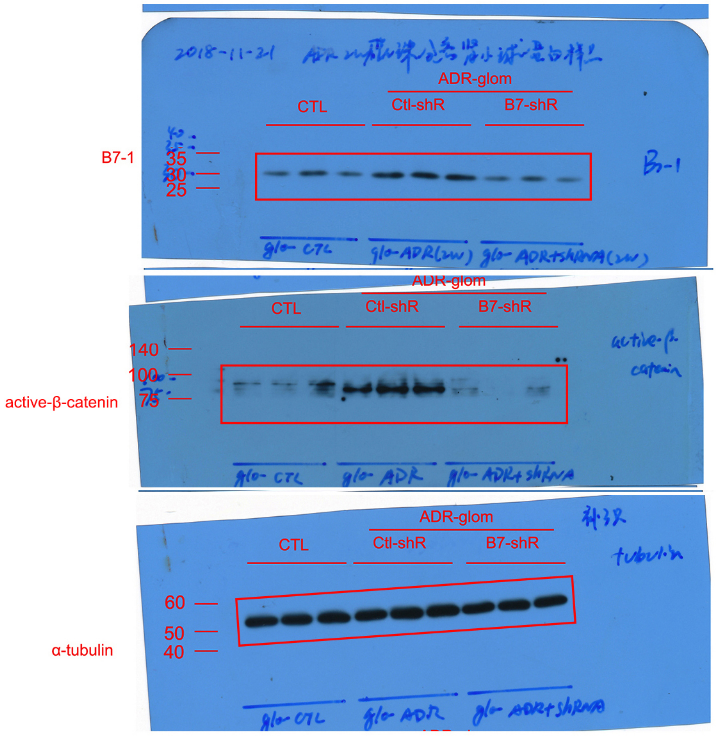

Fig. 3J

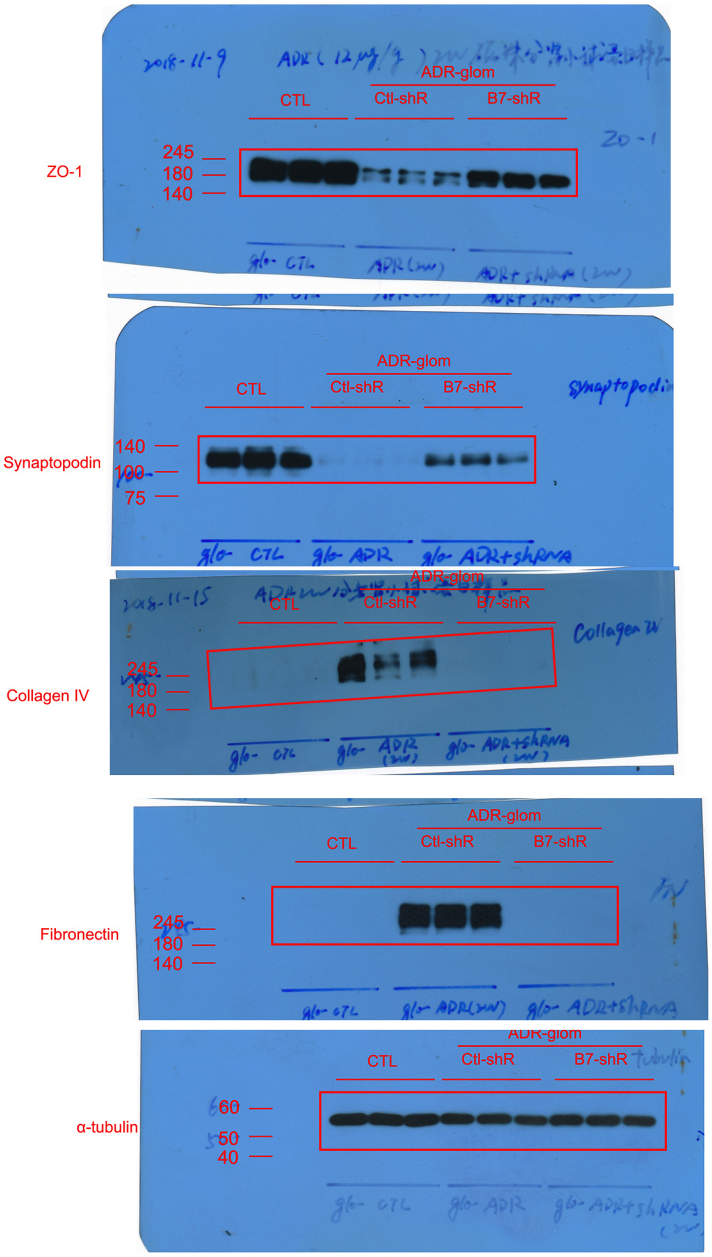

Full unedited gels for Figure 4

Fig. 4C

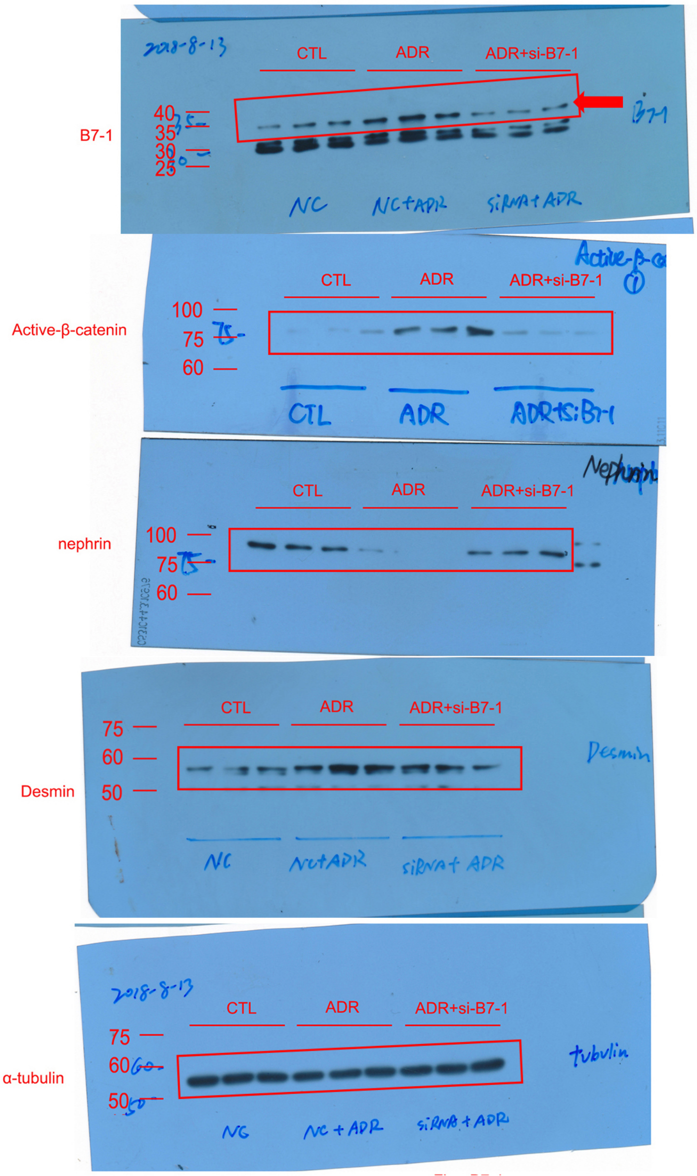

Fig. 4G

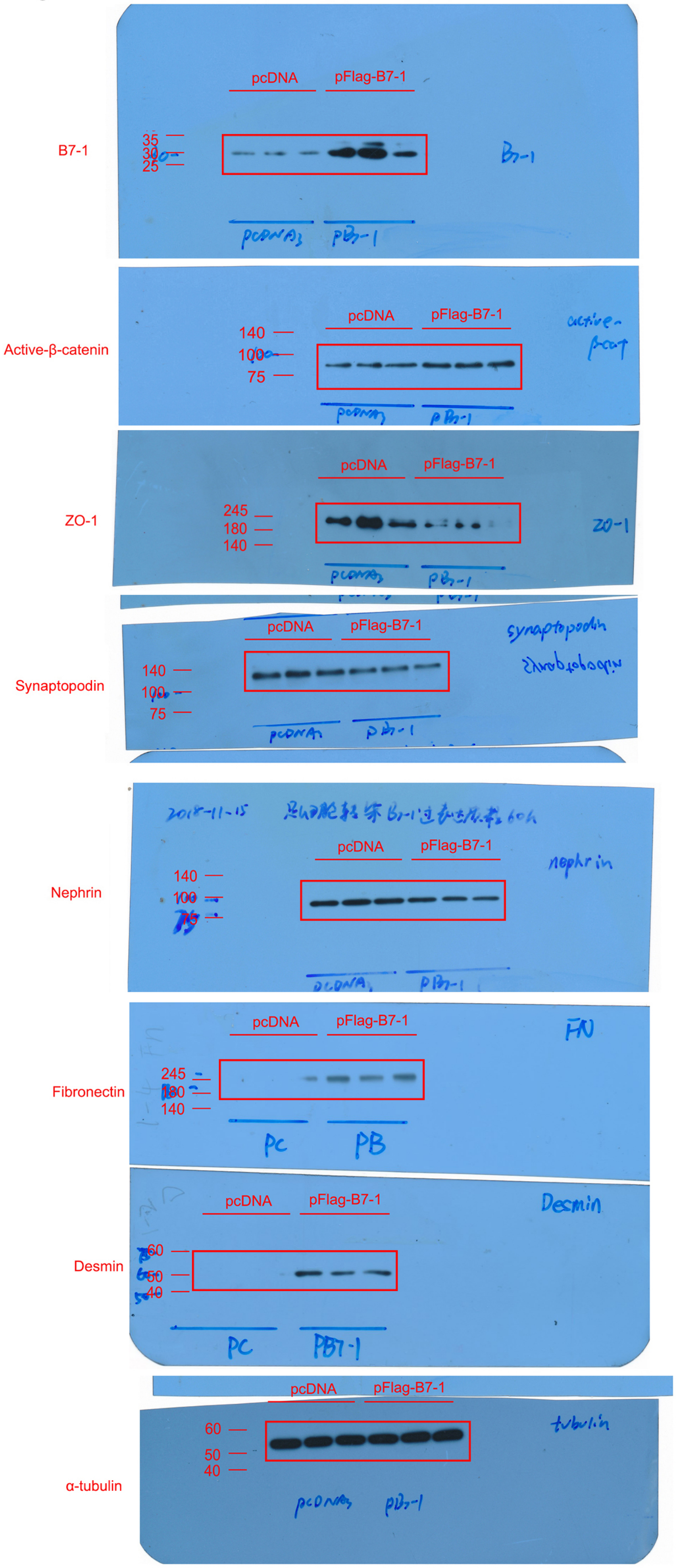

Full unedited gels for Figure 4

Fig. 4H

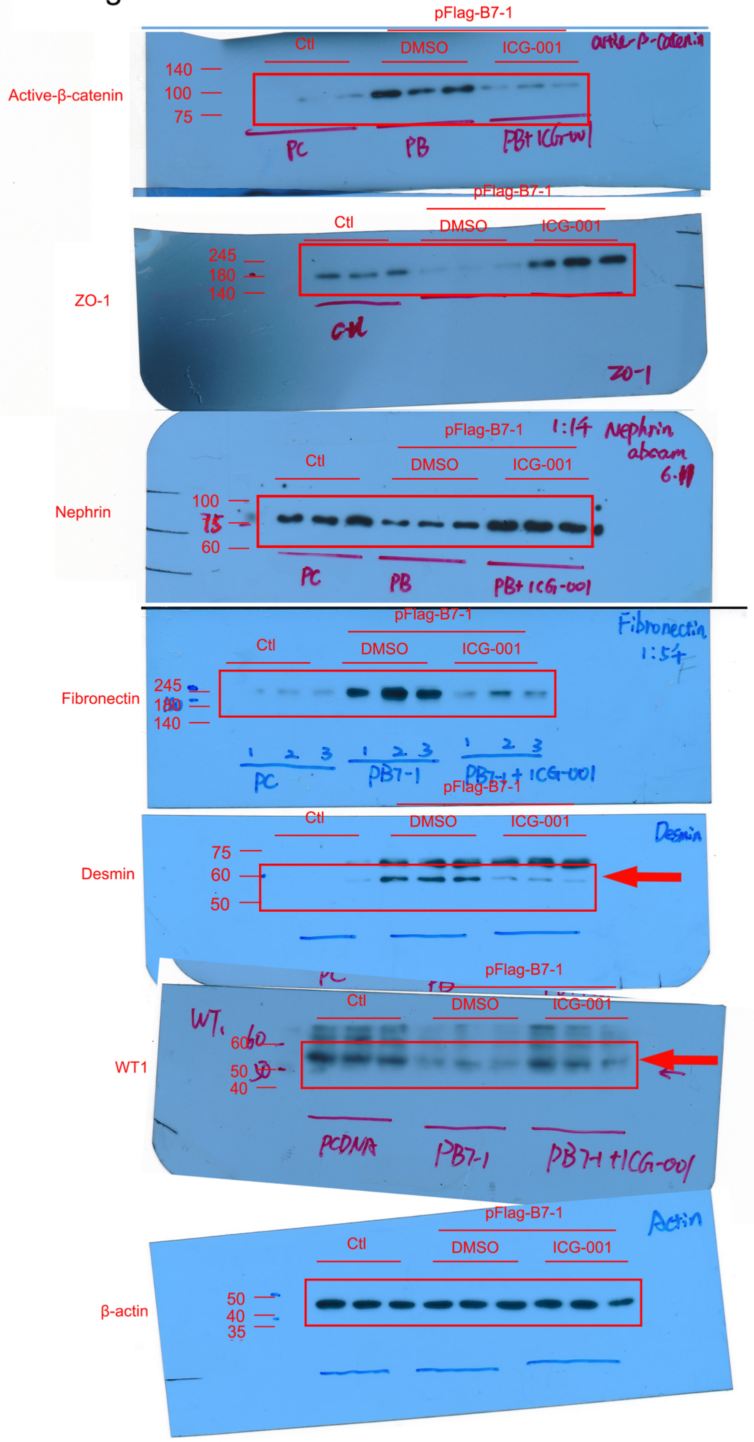

Fig. 4K

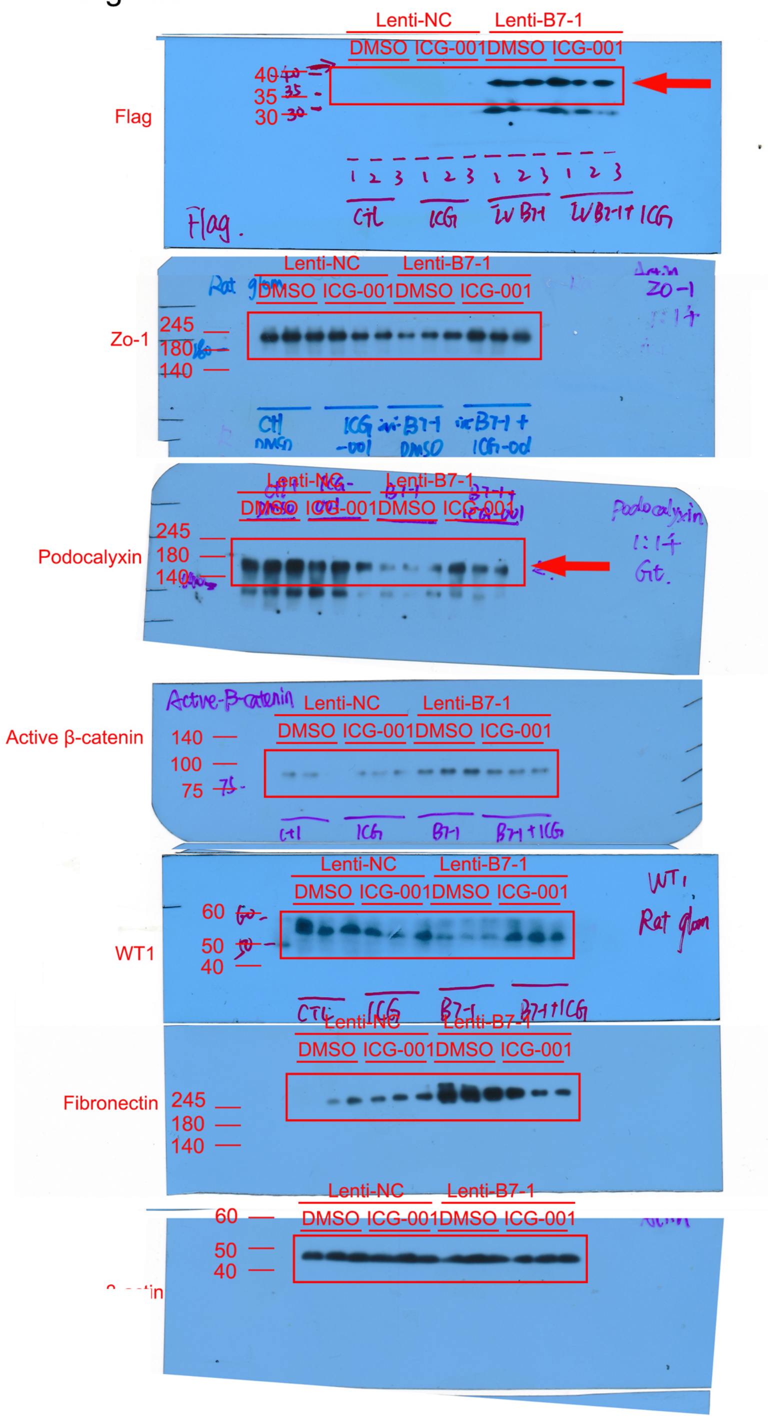

Fig. 4N

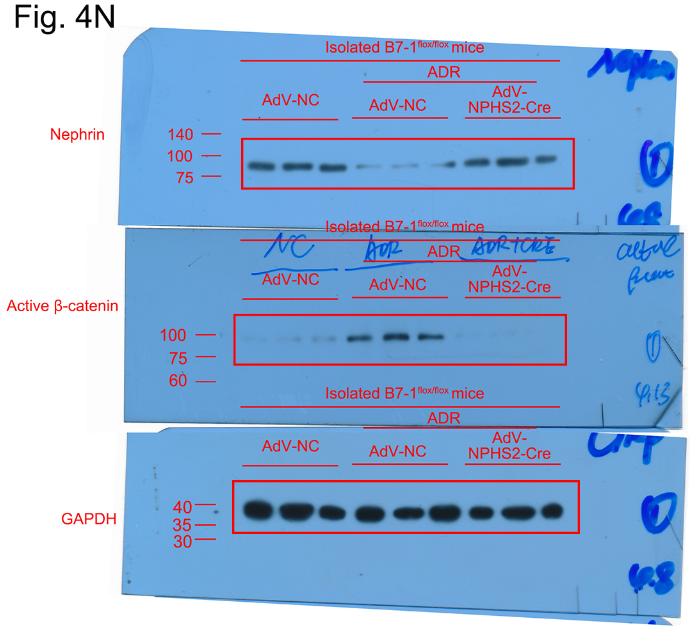

Full unedited gels for Figure 5

Fig. 5D

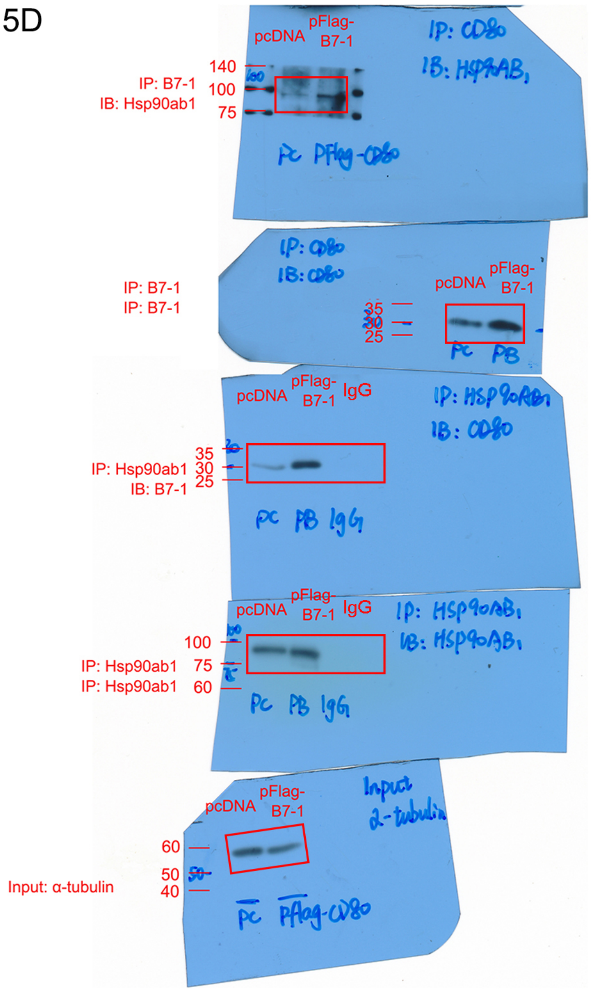

Fig. 5J

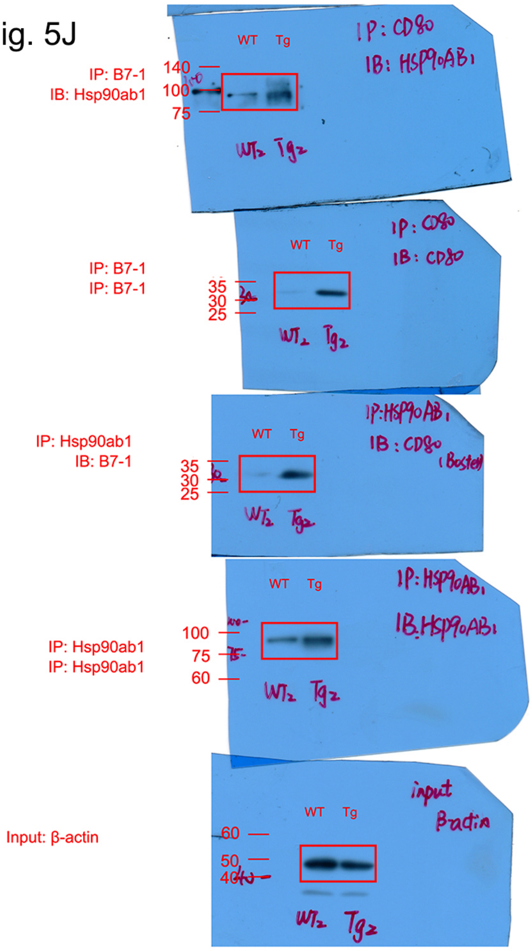

Fig. 5F

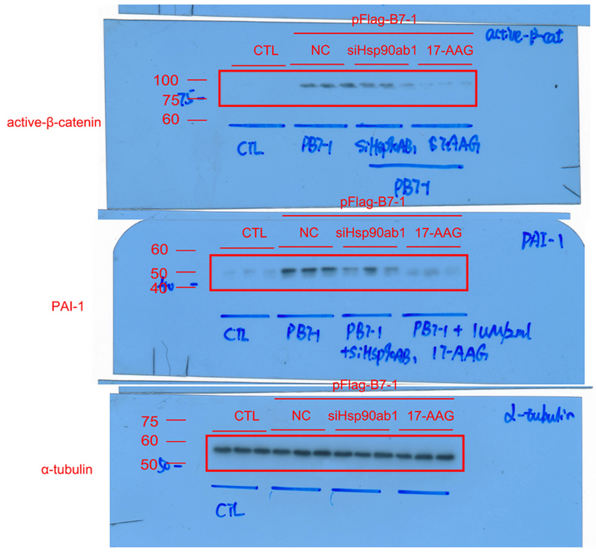

Fig. 5H

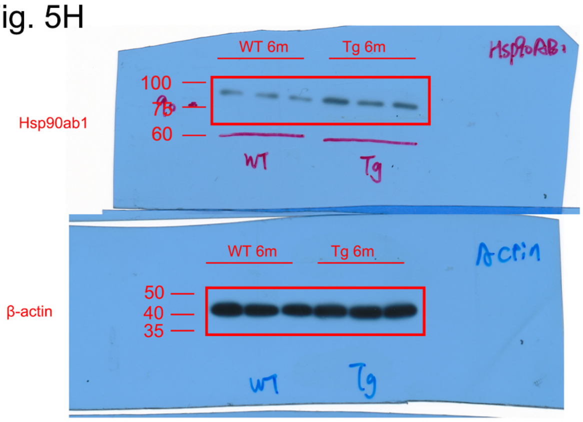

Full unedited gels for Figure 6

Fig. 6C

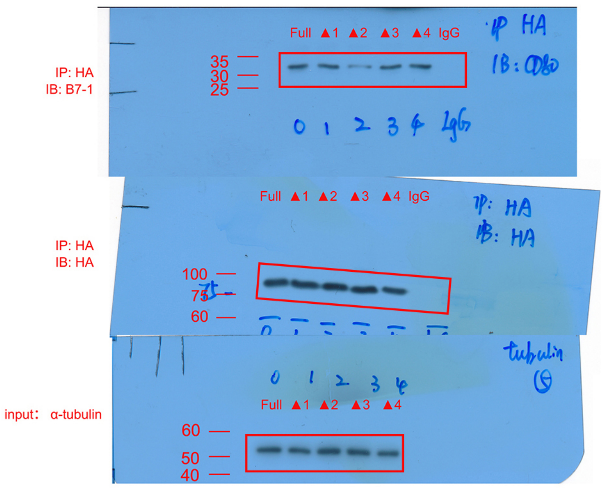

Fig. 6I

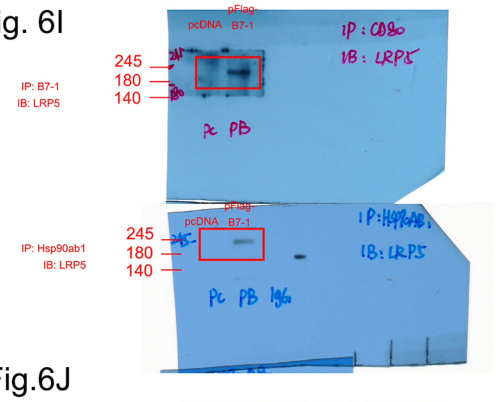

Fig.6J

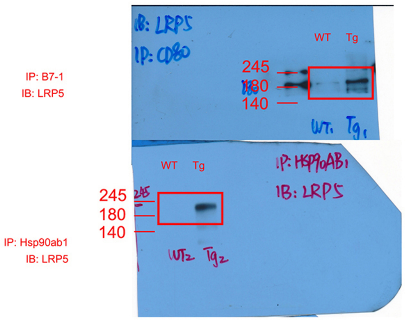

Fig.6K

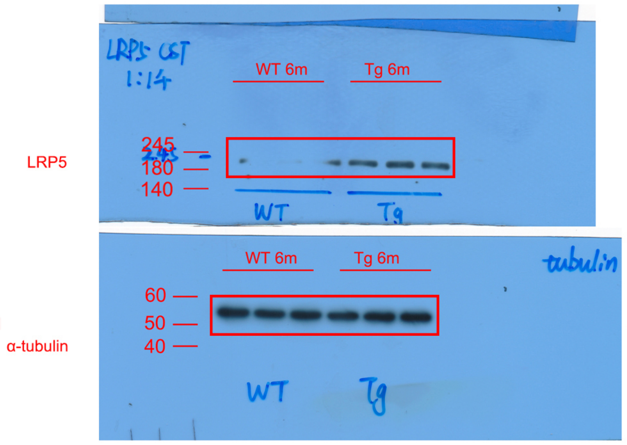

Fig. 6E

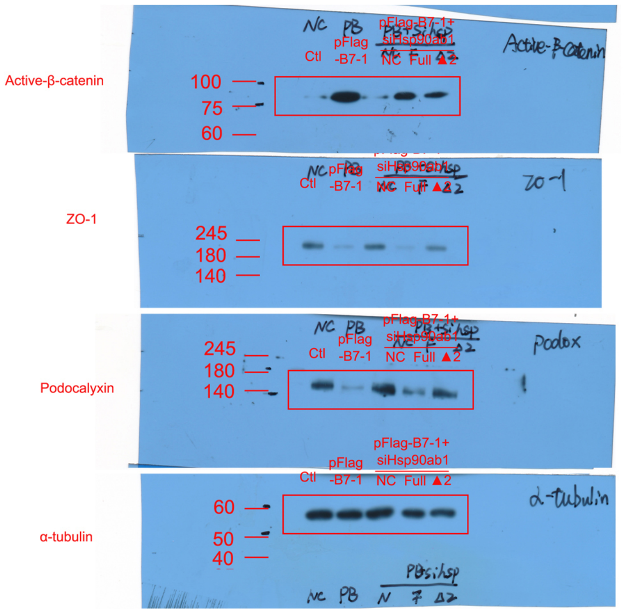

Fig. 6N

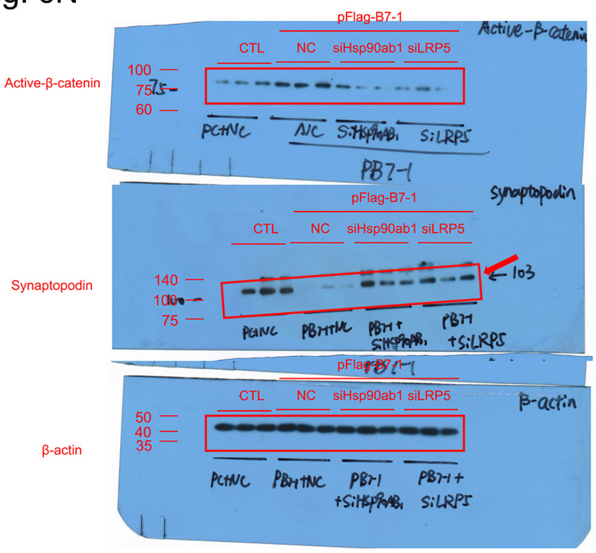

Full unedited gels for Figure 7

Fig. 7B

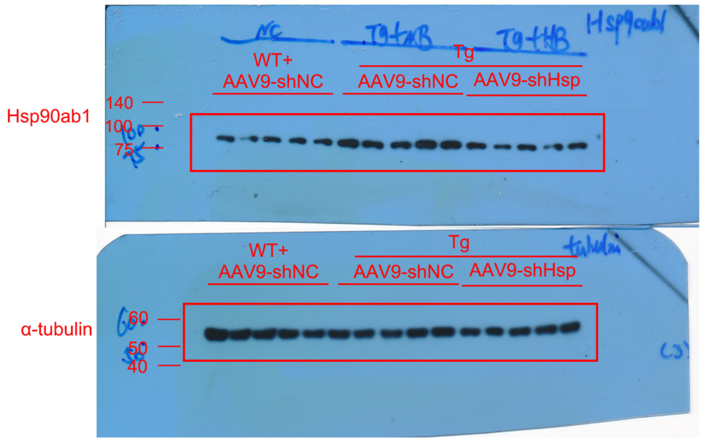

Fig. 7E

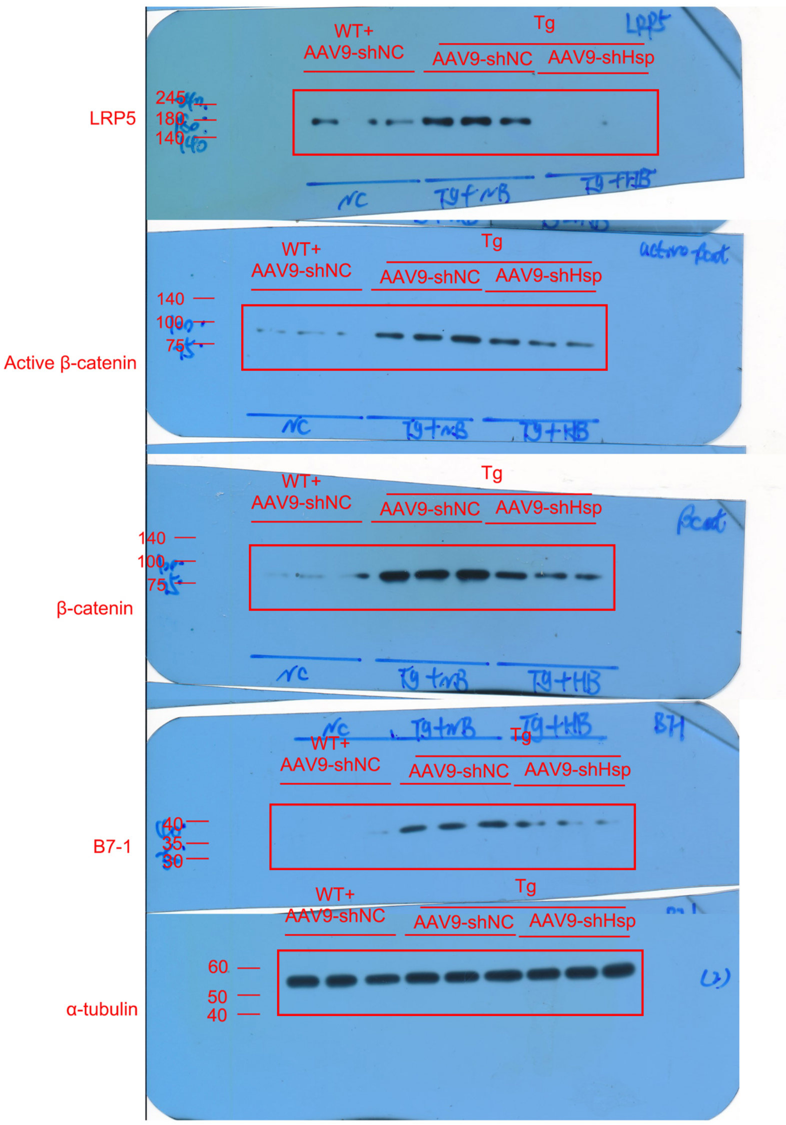

Fig. 7M

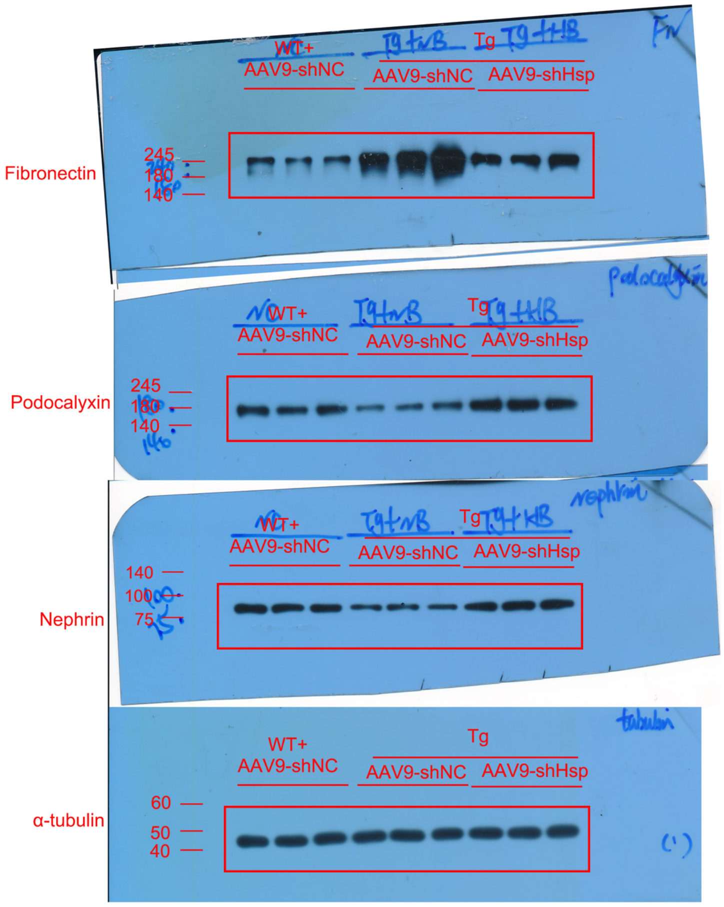

Full unedited gels for Figure 8

Fig. 8C

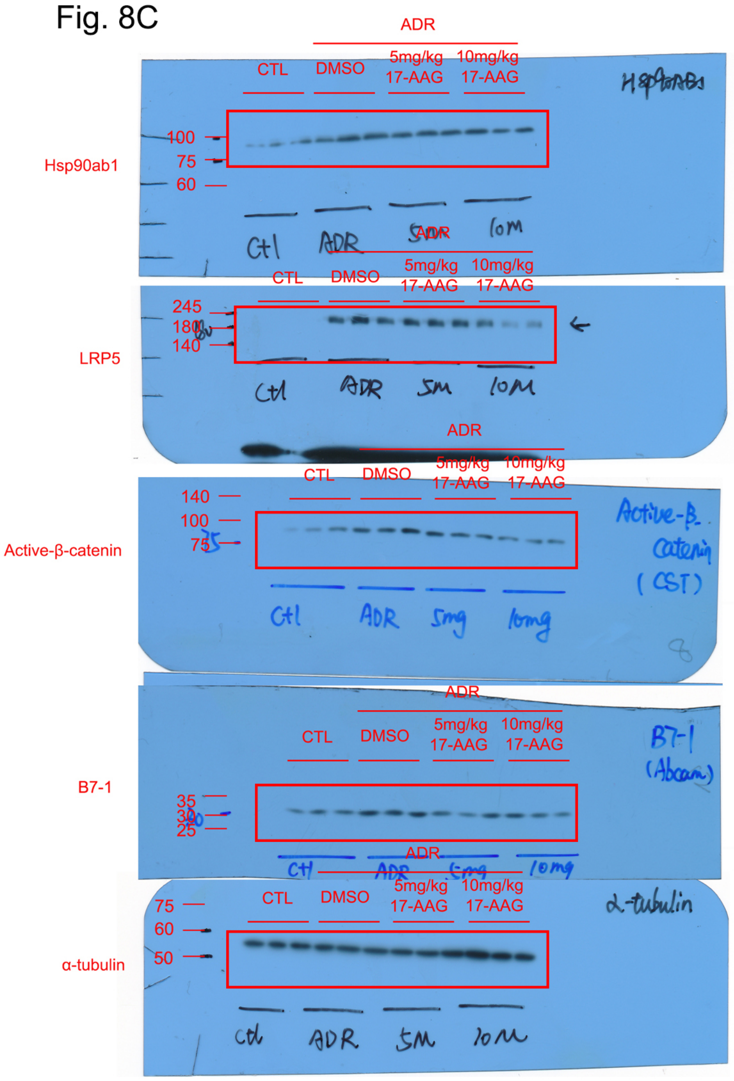

Fig. 8K

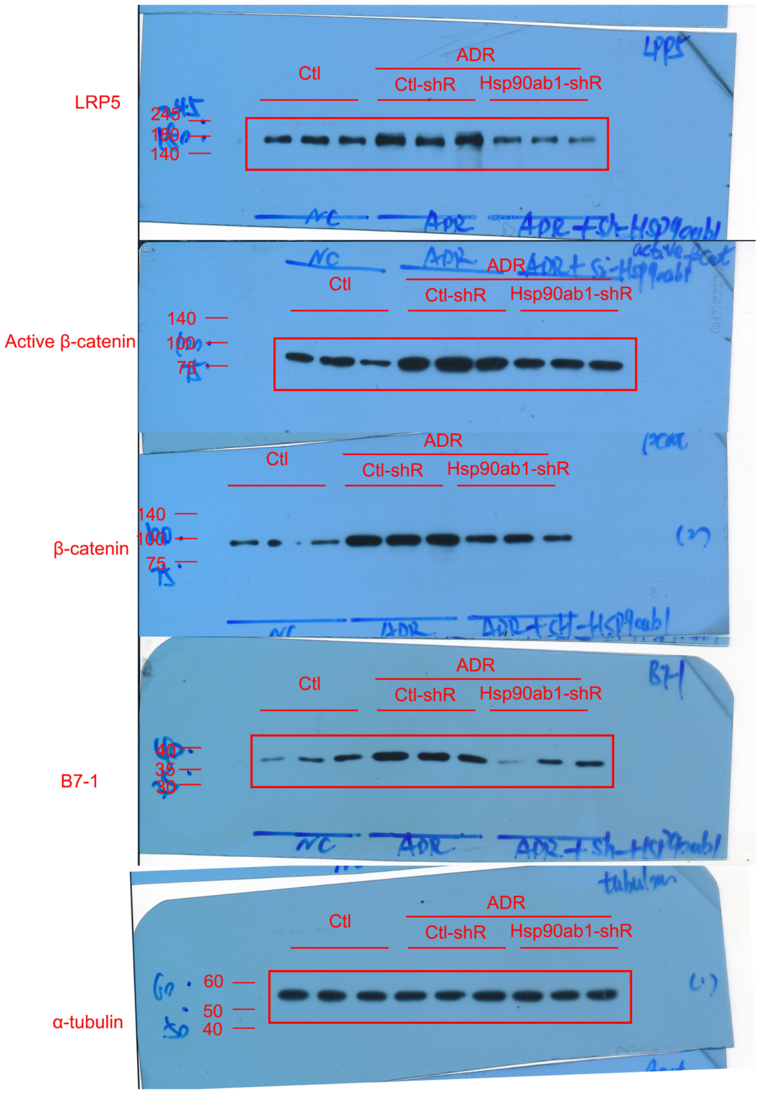

Fig. 8F

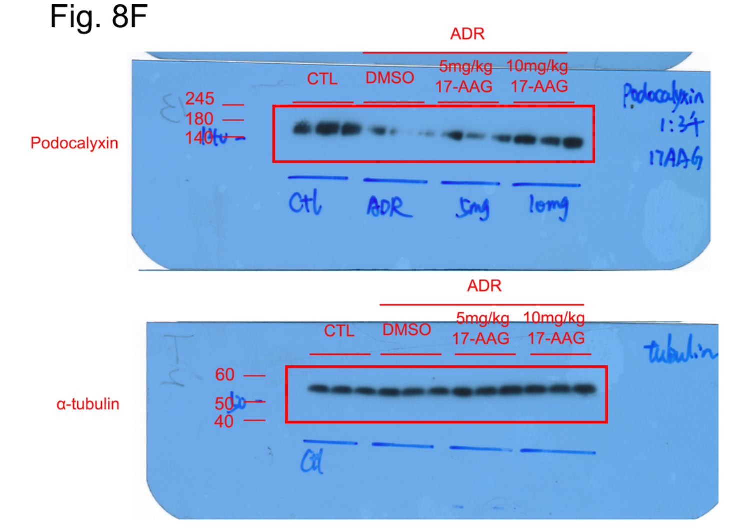

Fig. 8I

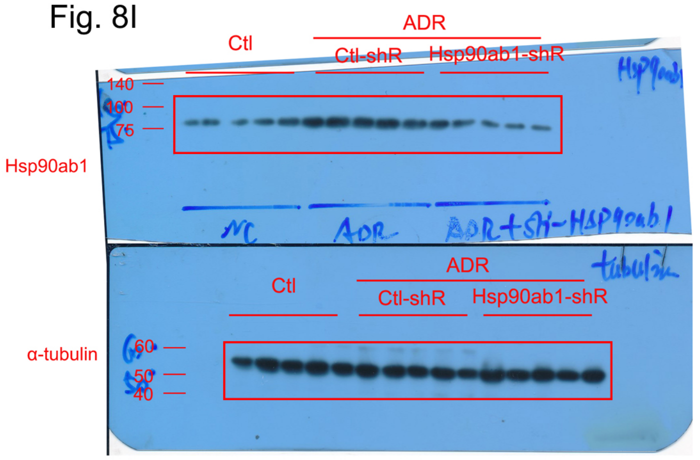

Fig. 8M

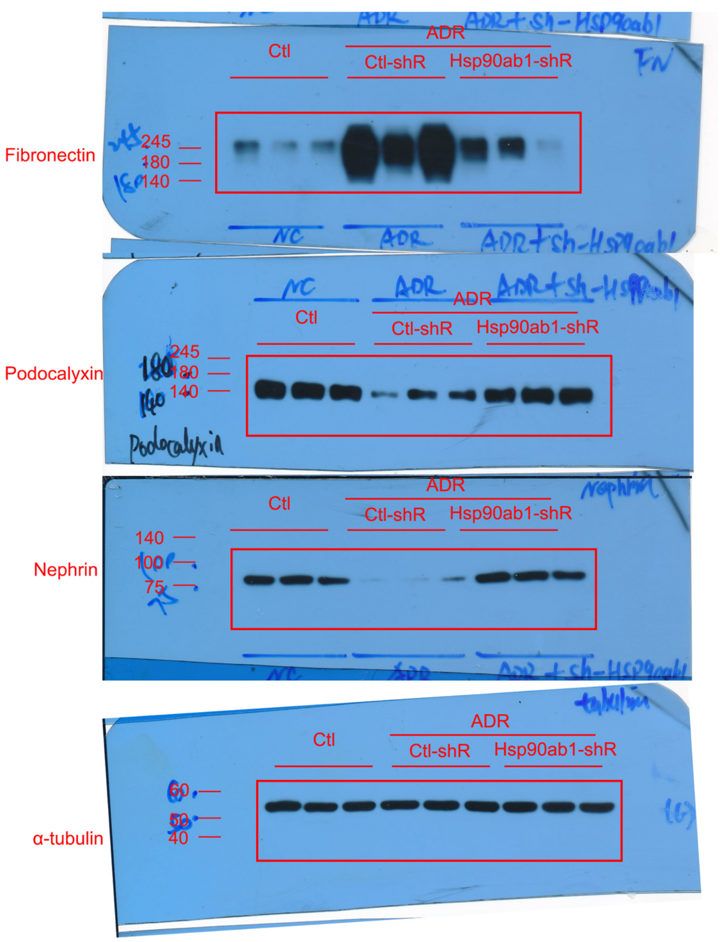

Full unedited gels for Figure 9

Fig. 9A

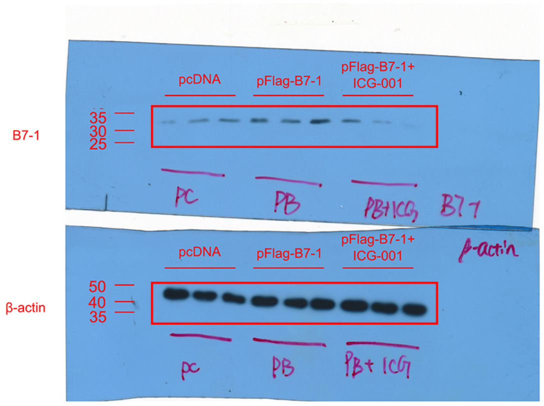

Fig. 9D

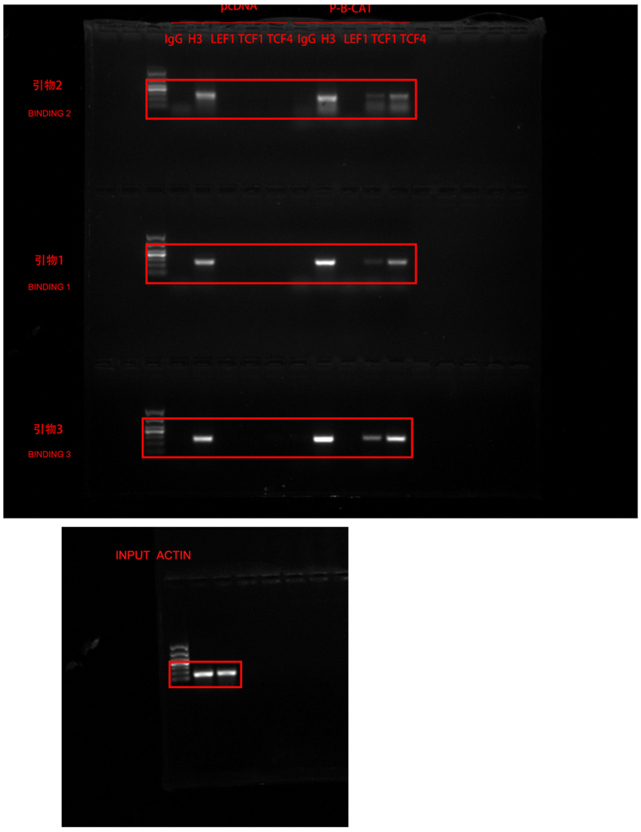

Fig. 9F

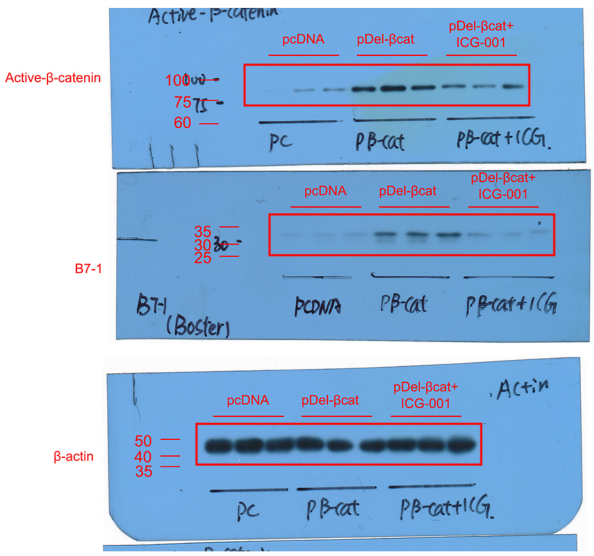

Fig. 9H

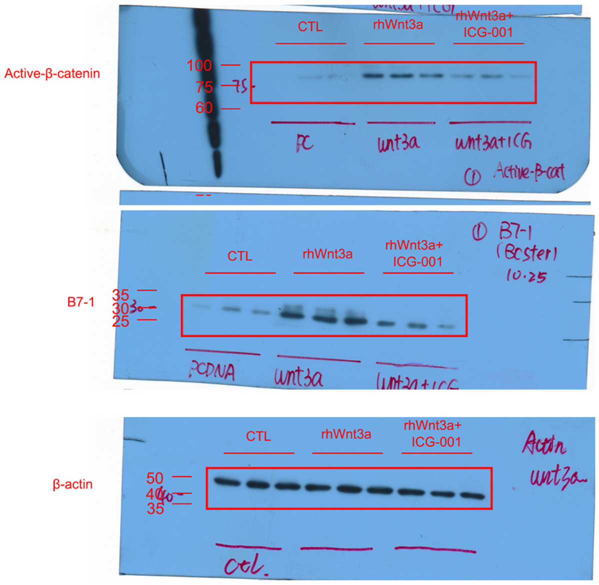

Fig. 9J

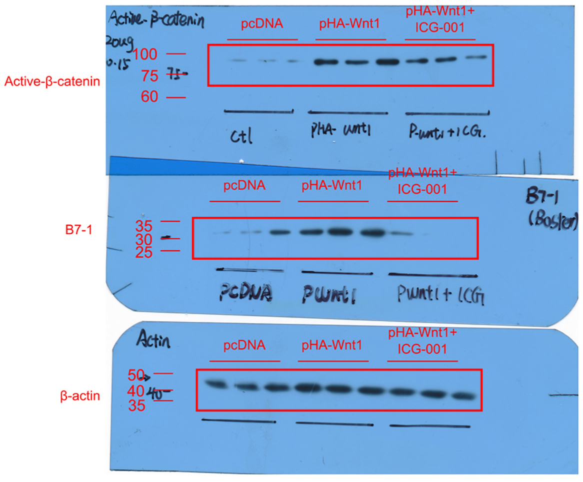

Full unedited gels for Supplementary Figure S2

Fig. S2B

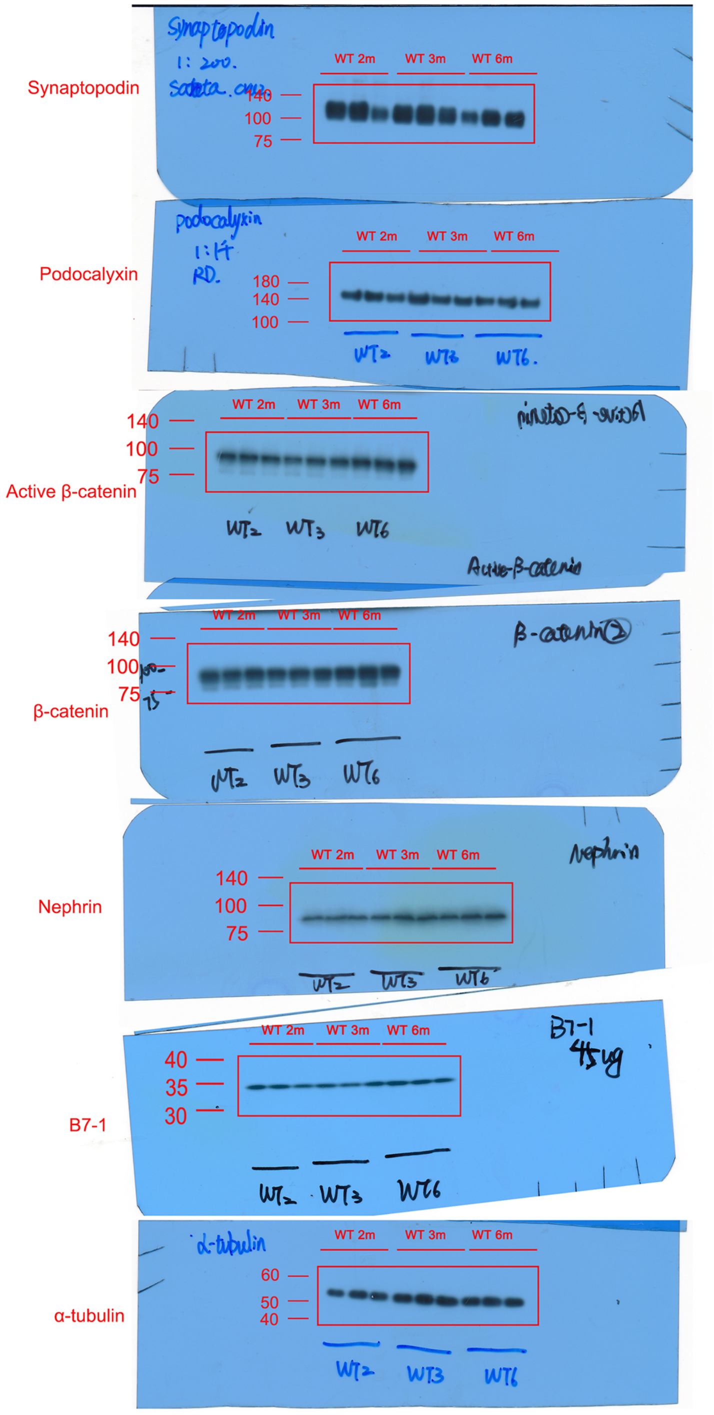

Fig. S2K

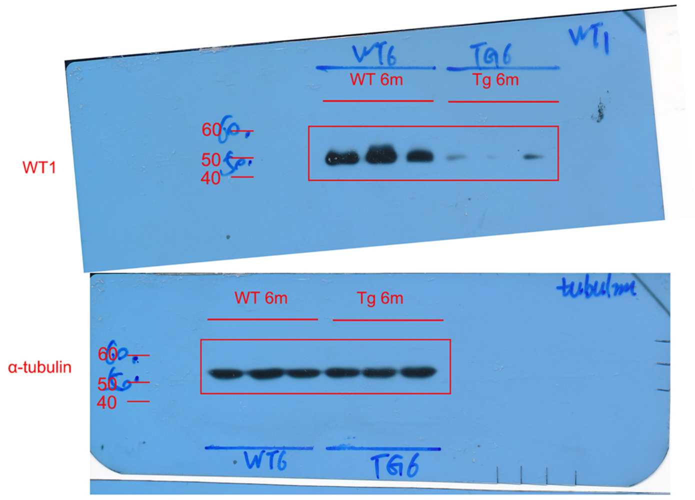

Full unedited gels for Supplementary Figure S4

Fig. S4F

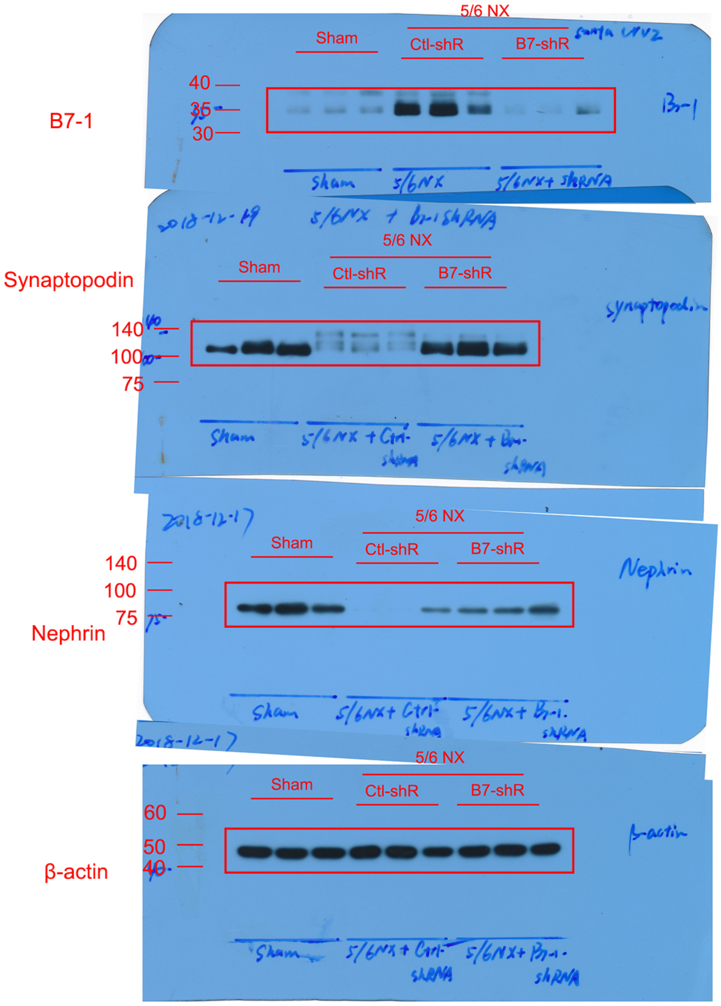

Fig. S4J

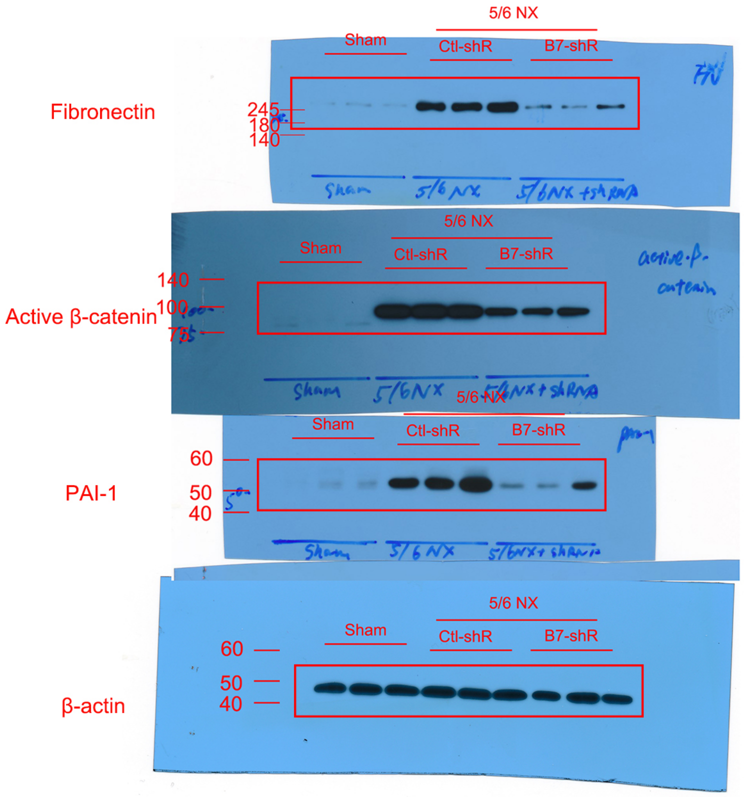

Full unedited gels for Supplementary Figure S5

Fig. S5C

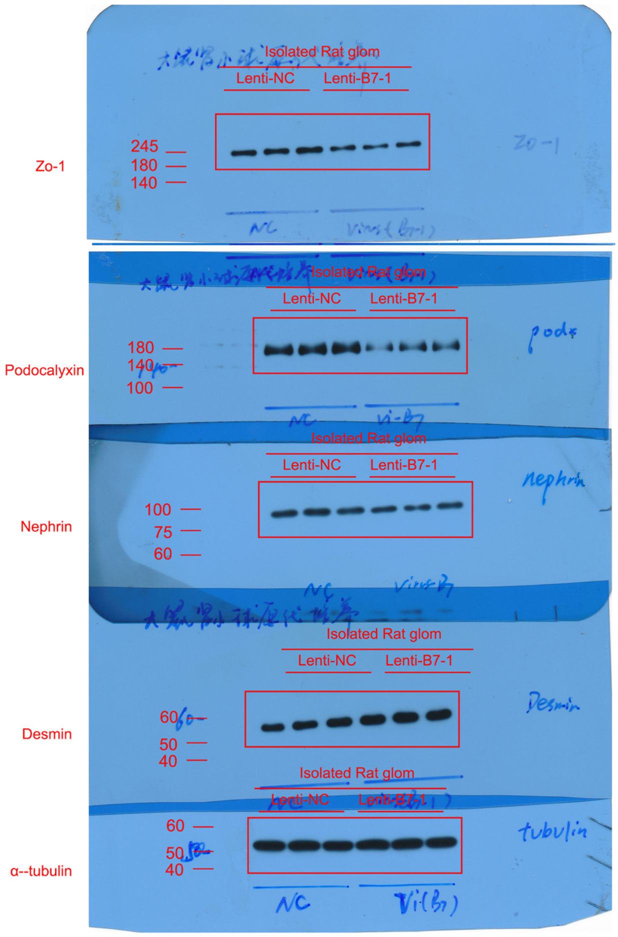

Fig. S5P

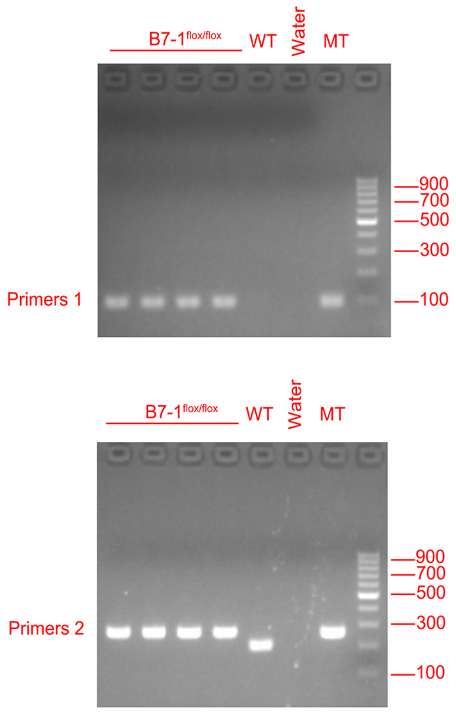

Full unedited gels for Supplementary Figure S8

Fig. S8B

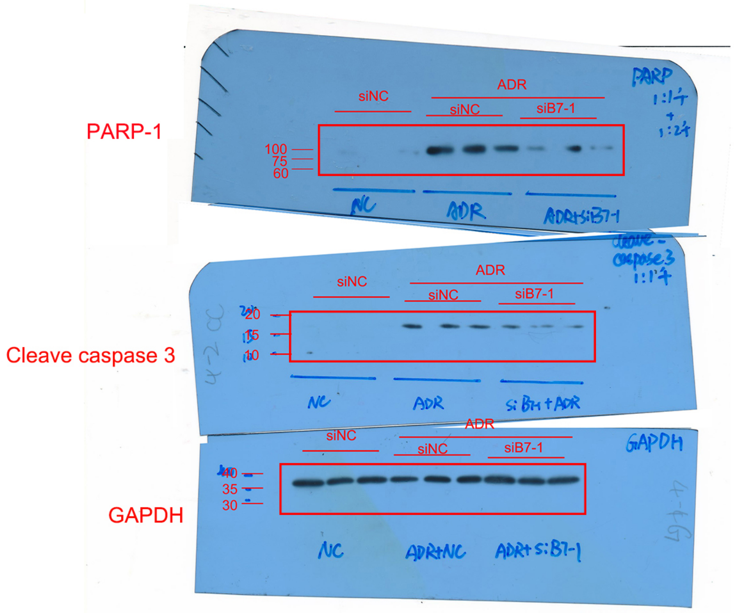

Fig. S8I

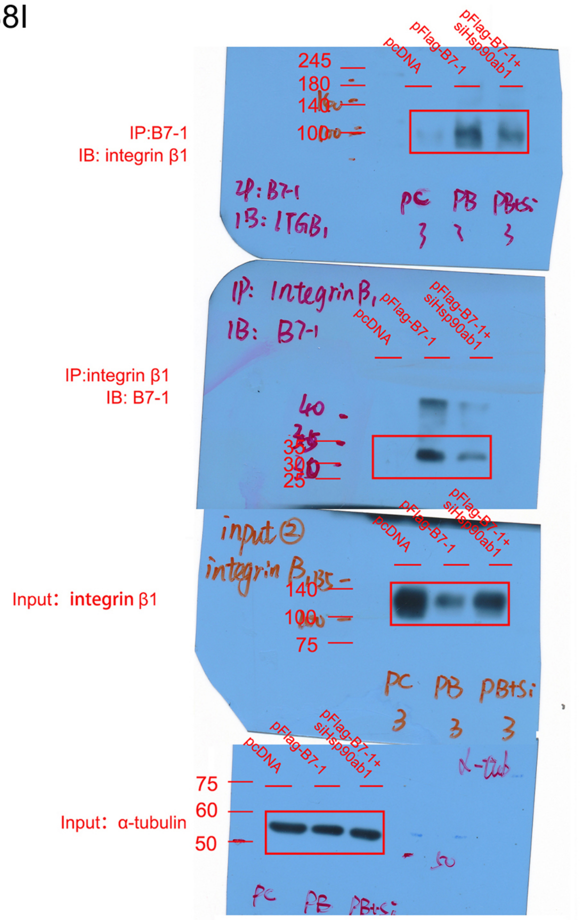

Supplement: Supplementary file 13 — Full unedited gels [file 41418_2022_1026_MOESM13_ESM.pdf]
